# Genome-wide identification and expression analysis of salt-responsive bHLH transcription factors in the wheat (*Triticum aestivum*) genome

**DOI:** 10.3389/fpls.2026.1770759

**Published:** 2026-04-23

**Authors:** Esteri Viitanen, Sameer Hassan, Alexander Vergara, Henrik Aronsson

**Affiliations:** 1 Department of Biological and Environmental Sciences, University of Gothenburg, Gothenburg, Sweden; 2 OlsAro Crop Biotech AB, Gothenburg, Sweden; 3 Instituto de Ciencias de la Ingeniería, Universidad de O’Higgins, Rancagua, Chile

**Keywords:** bHLH transcription factors, bHLH (MYC), salt stress, wheat, co-expression analysis, transcription factor enrichment analysis, crop improvement

## Abstract

Soil salinity is an increasing environmental constraint affecting agriculture worldwide, with major impacts on wheat (*Triticum aestivum*) productivity. Understanding the transcriptional regulatory mechanisms underlying salt tolerance is therefore essential for crop improvement. The basic helix–loop–helix (bHLH) transcription factor family plays important roles in plant stress responses. In this study, a genome-wide analysis of the Chinese Spring wheat reference genome identified five salt-responsive bHLH transcription factors: *TaMYC2-B, TaMYC2-D, FIT1*, and two *ORG2*/bHLH38-like paralogs. Using *in silico* promoter analysis, transcription factor enrichment analysis, and co-expression network analysis, we identified twelve predicted downstream target genes regulated by these transcription factors, including *RLK, CLC*, and *TaAPY*. RT-qPCR-based expression profiling of shoots and roots from the moderately salt-tolerant cultivar BARI Gom-25 exposed to 100 mM NaCl revealed tissue-specific regulation of several transcription factors and their putative target genes. *TaMYC2-B* and *TaMYC2-D*, along with their putative downstream targets *FOMT* and *CCoAOMT*, were downregulated in both tissues under salt stress. In contrast, a predicted *FIT1*-associated *CLC* gene was upregulated in roots despite reduced expression of the *FIT1* transcription factor. The apyrase gene *TaAPY* showed strong induction, particularly in roots, suggesting a role in extracellular ATP (eATP) turnover during stress. These expression patterns suggest transcriptional regulation potentially influencing ion homeostasis and stress signalling during salinity responses. Although functional validation is required to confirm these regulatory relationships, this study identifies candidate regulators and target genes that provide a foundation for future mechanistic studies and potential wheat improvement strategies.

## 1 Introduction

Large and rapidly increasing areas of salt-affected soils pose significant challenges to agriculture. Climate change and global warming directly affect both the crop yield and quality by intensifying the frequency and extent of abiotic stresses. Soil salinity affects more than 6% of land worldwide and it is one of the most severe abiotic stresses for crop plant productivity (Munns and Tester, 2008). Wheat (*Triticum aestivum*) is one of the most important staple crops globally and contribute a significant portion of many human populations’ daily calories and protein intake (Tadesse et al., 2017). While wheat consumption is increasing on a global level, over 50% of agricultural lands are expected to experience severe salinization by 2050 (Bhat et al., 2024; FAO, 2024). Salt stress induces a complex series of deleterious effects on plants, causing growth inhibition through alterations in membrane permeability and cellular osmotic balance, the generation of oxidative stress via reactive oxygen species (ROS), and the inhibition of enzymatic activity (Fujita et al., 2006; Shankar et al., 2015; El Sabagh et al., 2021). To address these challenges and maintain sustainable agriculture, understanding the molecular mechanisms involved in salt stress responses and tolerance is essential.

Many genes related to salt tolerance are regulated by transcription factors. One such family is the basic helix-loop-helix (bHLH) transcription factor family. bHLH transcription factors, which contain a conserved bHLH domain, form a large superfamily widely distributed in plants and play crucial roles in plant stress tolerance (Wang et al., 2019; Radani et al., 2023). The bHLH transcription factor superfamily consists of two highly conserved and functionally distinct domains, with a region of ca 60 amino-acid residues (Jones, 2004). The bHLH motif was first observed by Murre et al. in 1989, who formulated a classification for different bHLH proteins based on their tissue distributions, DNA-binding specificities, and dimerization potential (Murre et al., 1989; Murre et al., 1994). However, the classification has since then been extended into six major groups (A-F) which consider E-box binding, the conservation of amino acid residues in the motif, and the presence or absence of additional domains (Jones, 2004).

The first domain, located on the amino terminal, is the basic domain which functions by binding the transcription factor to DNA, also known as the E-box. The E-box is typically a consensus hexanucleotide sequence (CANNTG), and different bHLH proteins recognize these different E-box sequence motifs upon binding (De Martin et al., 2021; Thoben and Pucker, 2023). There are different types of E-boxes, the most common being the palindromic G-box (5′-CACGTG-3′), which allows the group of E-box binders to be further categorized into G-box binders and non-G-box binders (Gabriela et al., 2003; Carretero-Paulet et al., 2010; Zhang et al., 2020; Thoben and Pucker, 2023).

The second domain, located at the carboxy-terminal, allows interactions with other protein subunits to form homo- and hetero-dimeric complexes (Ellenberger et al., 1994; Jones, 2004; De Martin et al., 2021; Thoben and Pucker, 2023). Thus, it is possible to form several different combinations of dimeric structures, which in turn can bind monomers.

The heterogenous E-box, which can be recognized by different bHLH proteins in various forms, is the key for determining how diverse developmental functions are controlled through transcriptional regulation (De Martin et al., 2021). Some of the most common additional domains are the Per-Arnt-Sim (PAS) domain, located in the carboxy-terminal of the bHLH region, leucine-zipper domains (bZIP), and orange domain (Jones, 2004; Radani et al., 2023).

To date, several plant bHLH proteins have been characterized in many species. For example, there are 162 and 167 bHLHs in Arabidopsis (*Arabidopsis thaliana*) and rice (*Oryza sativa*), respectively (Bailey et al., 2003; Gabriela et al., 2003; Li et al., 2006). However, less is known about the bHLH family in wheat, which is one of the most important crops worldwide (Filip et al., 2023). Progress in our understanding of wheat gene families has been proceeding slower compared to that of other species due to the complexity of the allohexaploid wheat genome (2n = 6x = 42, AABBDD). Given the importance of wheat as a staple crop and the critical roles plant bHLH transcription factors have in abiotic stresses, particularly under salt stress, it is imperative to investigate the roles of bHLH transcription factors in conferring salt stress tolerance. To elucidate the role of bHLH transcription factors in wheat salt tolerance, we investigated bHLH mediated regulation of gene expression and metabolic pathways in wheat with respect to salt stress.

In this study, we investigate the potential role of MYC2-type bHLH transcription factors in wheat salt stress responses, focusing on two paralogs, *TaMYC2-B* and *TaMYC2-D*, as candidate regulators of downstream stress-associated genes. We also identify a predicted *FIT1*-associated chloride channel (CLC) that may contribute to ion homeostasis under salinity, alongside several additional bHLH transcription factors and their putative targets. Particular attention is given to a wheat apyrase, *TaAPY*, whose expression pattern suggests a possible contribution to enhanced salt tolerance. Together, these analyses provide new insights into the regulatory landscape of bHLH transcription factors in wheat and highlight candidate genes and pathways that may inform future strategies for improving salt resilience in wheat breeding.

## 2 Materials and methods

### 2.1 Plant growth conditions and treatments

BARI Gom-25, a moderately salt tolerant wheat variety developed by the Bangladesh Agriculture Research Institute (BARI) and previously used for salt tolerance assays, was used for this study (Lethin et al., 2020; Lethin et al., 2022). Seeds were germinated on wet filter paper (Munktell-filter paper – A1-100-80™) for four days in the dark at room temperature. Seedlings were transferred to a hydroponic cultivation system on the morning of the fourth day. The hydroponic cultivation system contained deionized water supplemented with Nelson Garden Hydroponic Nutrition™ (2 ml/L), and the pH was adjusted to 6.0 using HCl. After seven days of hydroponic growth, the seedlings were transferred to a new hydroponic solution with or without 100 mM NaCl. The 7-day salt exposure period was selected to assess transcriptional responses under prolonged salinity conditions rather than transient early stress responses. At day 14, shoots and roots were collected separately, immediately snap-frozen in liquid nitrogen, and stored at -80 °C until further use. Each biological replicate of the root and shoot samples consisted of a pool of four individual plants from both the control and the 100 mM NaCl treated groups.

### 2.2 Reverse transcription-quantitative PCR and expression analysis

Frozen plant shoot and root materials were pulverized using a MM 301 Mixer Mill (Retsch GmBH). Total RNA extraction from the tissues was conducted as per manual of the kit NucleoSpin RNA PlantTM (Macherey-Nagel). The qScript Ultra SuperMix (Quantabio) was used for synthesis of cDNA from plant root and shoot tissues using 1000 ng total RNA.

Reverse transcription-quantitative PCR (RT-qPCR) assay was conducted to confirm and analyze expression levels of the selected candidate genes. Gene-specific primers were designed using NCBI Primer blast website. The primers used for RT-qPCR are listed in the **Supplementary Table 1**. RT-qPCR analyses were carried out using a BioRad CFX96 Real TimeTM system by following the manufacturer’s instructions for SsoAdvanced Universal SYBR Green Supermix™ (Bio-Rad). Each reaction had three independent biological replicates and four technical replicates. Actin and Glyceraldehyde-3-phosphate dehydrogenase (GAPDH) were used as reference genes for normalization of candidate gene expression. The expression stability of Actin and GAPDH was evaluated across control and 100 mM NaCl-treated samples in both shoot and root tissues, and Cq values showed minimal variation between conditions, supporting their suitability for normalization. Relative expression levels were calculated using the 2^^−ΔΔCT^ method, with NaCl-treated samples normalized to the corresponding untreated control samples within the same tissue type. Statistical analyses were performed separately for each tissue, and treatment effects were assessed by comparing NaCl-treated samples with their respective controls using a two-tailed Student’s t-test. No statistical comparisons were performed between different tissue types. The error bars in the results represent the standard error of the mean (SEM) calculated from three independent biological replicates and quadruplicate PCR reactions for each sample.

### 2.3 Identification of bHLH sequences

The Hidden Markov Model (HMM) file of the bHLH domain (PF00010) was downloaded from the PFAM database to identify bHLH sequences in the wheat genome (Pfam version 32.0). To match the bHLH-sequences with the wheat genome, the proteome of the wheat cultivar Chinese Spring genome was downloaded from the Ensemble plants database (http://ftp.ensemblgenomes.org/pub/release-61/plants/fasta/triticum_aestivum/pep/Triticum_aestivum.IWGSC.pep.all.fa.gz) and used as a reference. The HMM profile of the bHLH domain was used as a query to scan across the wheat proteome using HMMER software (version 3.1) with a default E value. The extracted protein sequences were obtained from the proteome file using an in-house Python script and the sequences were used for a batch search in PFAM to determine the corresponding bHLH domains. This resulted in a total of 377 bHLH transcription factors with a bHLH domain sequence. The redundant protein sequences were identified using CD-HIT (https://github.com/weizhongli/cdhit) with a sequence identity cut-off of 100%, and the representative sequences were selected for further analysis.

The bHLH sequences were further analyzed to find how prevalent and distributed they were across the public RNAseq datasets involved in wheat salt tolerance available at NCBI by using a foldchange threshold of 1.0. The most prevalent bHLH sequences were selected for further analysis. In addition, published literature was consulted to verify previously reported roles or associations of the selected genes with salinity or abiotic stress responses, thereby supporting their biological relevance. This resulted in the selection of five bHLH transcription factors: *TaMYC2-B* (TraesCS1B02G208000), *TaMYC2-D* (TraesCS1D02G196900), FER-like iron deficiency-induced transcription factor (*FIT1*, bHLH29, TraesCS2D02G280100), *ORG2*-like (bHLH38-like, TraesCS3A02G489500) and ORG2*-like (bHLH38-like, TraesCS3D02G495400).

### 2.4 Transcription factor binding site prediction

To determine putative target genes for the five bHLH transcription factors, the upstream regions (2000 bp) of all the genes were extracted from the International Wheat Genome Sequencing Consortium (IWGSC) Chinese spring wheat genome accessed via the Ensembl plants database. The Bedtools get fasta option was used to extract upstream sequences of the individual genes. The bHLH sequences were also subjected to profile inference tool (https://jaspar.elixir.no/inference) analysis to identify their JASPAR transcription factor-binding profiles. An in-house Python script (https://github.com/Sameerpython/Transcription-Factors, accessed on 2^nd^ of April 2024) was written to perform batch search against the JASPAR database. The bHLH transcription factors were divided into two categories: downregulated and upregulated, respectively. The foldchange threshold used for each category was set to 1.0.

Finally, the Position Weight Matrices (PWMs) for each binding motif (MYC2, MYC3, MYC4, bHLH3, bHLH13, AIB and bHLH18) corresponding to the five bHLH transcription factors were obtained in MEME format from the JASPAR CORE database. The upstream region of every gene was then scanned against the PWMs for predicting the bHLH transcription binding site with a p-value < 1e-5 using the Find Individual Motif Occurrences (FIMO) tool from the MEME Suite (http://meme-suite.org/index.html, accessed on 3rd of April 2024) (Bailey et al., 2015). The FIMO tool scans sequences matching each of the bHLH motifs collected from the JASPAR database.

### 2.5 Identification and extraction of target genes

Target genes were extracted based on gene IDs from the FIMO analysis using R, and potential functional salt-stress-related genes were identified. Furthermore, the target genes were analyzed against the *in silico* upregulated bHLH transcription factors, and against the *in silico* downregulated bHLH transcription factors, respectively. The target genes were identified and ranked according to the best p-value and q-values from the FIMO analysis. These target genes were compared against the target genes identified from the public RNAseq datasets involved in wheat salt tolerance available at NCBI (https://www.ncbi.nlm.nih.gov/bioproject/).

This resulted in selecting the most prominent salt related target genes: six target genes regulated by the *in silico* upregulated bHLH transcription factors, and six target genes regulated by the *in silico* downregulated bHLH transcription factors, respectively.

### 2.6 Mapping of DNA-binding residues in bHLH transcription factors

The protein sequence of bHLH was searched against the Protein Data Bank (PDB) using the BLAST tool. The experimentally determined structure of the bHLH domain in interaction with DNA (PDB ID: 5GNJ) was identified and aligned with the bHLH transcription factor domain sequences (see section 2.3 above) from this study. The amino acids binding to the E-box DNA sequence were identified using the PDBSum database. The identified binding residues were mapped to the multiple sequence alignment of the identified bHLH domain sequences and a WebLogo (https://weblogo.threeplusone.com/) was generated.

### 2.7 Chromosomal location of bHLH transcription factors

The chromosomal positions of the bHLH transcription factors in wheat were obtained from the Ensembl Plants Gramene database. The distribution of the bHLH transcription factors on the chromosomes was generated using the *Triticum aestivum* karyotype in Ensembl Plants (https://ensembl.gramene.org/Triticum_aestivum/Location/Genome).

### 2.8 RNA-Seq analysis

The raw data for 71 RNA-Seq samples from seven Bioprojects were downloaded from NCBI SRA database. The reads were then mapped against the wheat reference genome (IWGSC, https://plants.ensembl.org/Triticum_aestivum/Info/Annotation/#assembly) using STAR (v 2.7.11). The gene-level count data were then generated using featureCounts (Subread v 2.1.1), by assigning the reads to the annotated genes based on wheat genome annotation file. The raw count matrix was then normalized using PyDESeq2 (v0.5.4) which implements the DESeq2 median of ratios method for correcting the sequencing differences in library size and RNA composition across samples. The normalized counts were used for the downstream analysis, including differential expression and gene co-expression network construction. Differentially expressed genes were identified by filtering Log2FoldChange greater than 2 and a p-value < 0.05.

### 2.9 Transcription factor enrichment analysis

TF binding site motifs were obtained from the CIS-BP database (v3.0, updated 2024) (Weirauch et al., 2014) for *Triticum aestivum*. Position weight matrices (PWMs) were converted to IUPAC consensus strings using standard degeneracy codes, yielding 1,195 unique motifs. These motifs were searched against the 2 kb upstream promoter sequences of all genes in the genome using Patmatch (v1.2) with no mismatches allowed, on both strands. To assess whether specific transcription factor families were overrepresented among the members of the co-expression communities detected by Infomap, a one-sided Fisher’s exact test was performed using the Patmatch results and the TF–family mappings from CIS-BP. The enrichment analysis was interpreted at the family level. Results were considered significant at p < 0.05. Gene IDs were converted using the ID conversion resource from the wheat integrative regulatory network (wGRN) (Chen et al., 2023).

### 2.10 Co-expression network construction and visualization

A genome-wide gene co-expression network was constructed using the RNA-seq expression data from *Triticum aestivum*. 71 samples were used as described in section 2.8 above. Pairwise Spearman rank correlation coefficients were computed across all expressed genes. Gene pairs were retained as edges in the network if they met both of the following criteria: a Spearman correlation coefficient ≥ 0.7 and a Bonferroni-corrected p-value < 0.01. The resulting network comprised 87,908 nodes and approximately 52 million edges. Community detection was subsequently performed using the Infomap algorithm (v2.9.2) (Rosvall and Bergstrom, 2008). Network visualization was performed in two complementary formats using the *ggraph* (v2.2.1) and *visNetwork* (v2.1.2) packages in R.

## 3 Results

### 3.1 RNAseq and TF enrichment analysis

To investigate the transcriptional response under salt stress, a total of 71 samples derived from seven bioprojects were analyzed. Only samples corresponding to root and leaves were selected to ensure consistency across the datasets. After preprocessing and normalization, differential gene expression analysis was performed to identify genes that are significantly up and down regulated. Using a threshold of log2 foldchange >=2 and a p-value <=0.05, significantly differentially expressed genes (DEGs) were filtered and used for downstream analysis (**Supplementary Figure 4**).

To gain further insight into the regulatory mechanism underlying under salt stress, transcription factor enrichment analysis was performed using the DEG filtered set. This analysis revealed an overrepresentation of the bHLH transcription factor family, suggesting that this family of transcription factors plays an important role under salt stress. Based on this result, we focused on wheat genome-wide analysis of this family to identify potential targets and to investigate their co-expression patterns.

### 3.2 Domain architecture of bHLH transcription factors

**Figure 1:**
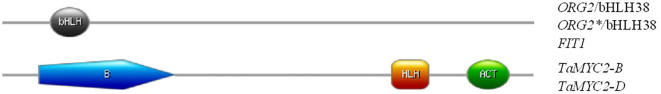
Domain organization of the five bHLH transcription factors associated with salt stress in this study. Two different domain architectures in which the bHLH domain appears were observed in the Chinese Spring wheat. The ORG2-like/bHLH38, ORG2*-like/bHLH38 and FIT1 transcription factors had one bHLH domain. TaMYC2-B and TaMYC2-D both had an ACT domain, a bHLH-MYC N-terminal domain (B) and a helix-loop-helix DNA-binding domain (HLH). ACT, ACT-like domain (PF22754); bHLH, bHLH domain (PF00010); B, bHLH-MYC N terminal domain (PF14215); HLH, Helix-loop-helix DNA-binding domain (PF00010). Source: Created using MyDomains Image Creator.

bHLH transcription factors are identified to have two distinct domains, the basic domain which binds the transcription factor to the DNA, and the HLH domain, which facilitates interactions with other protein subunits to form homo- and hetero-dimeric complexes (**Figure 1**). Among the five bHLH transcription factors selected for further analysis, *TaMYC2-B* and *TaMYC2-D*, had a helix-loop-helix (HLH) domain that mediates protein dimerization, an extra basic region (B) adjacent to the HLH domain that specifically binds to DNA, and an additional ACT-like domain that mediates protein-protein interactions (**Figure 1**). The three other bHLH transcription factors, *FIT1* and the two paralogous *ORG2-like/bHLH38* bHLH transcription factors had one integrated bHLH domain (**Figure 1**).

### 3.3 Mapping of DNA-binding amino acids in bHLH transcription factors

**Figure 2:**
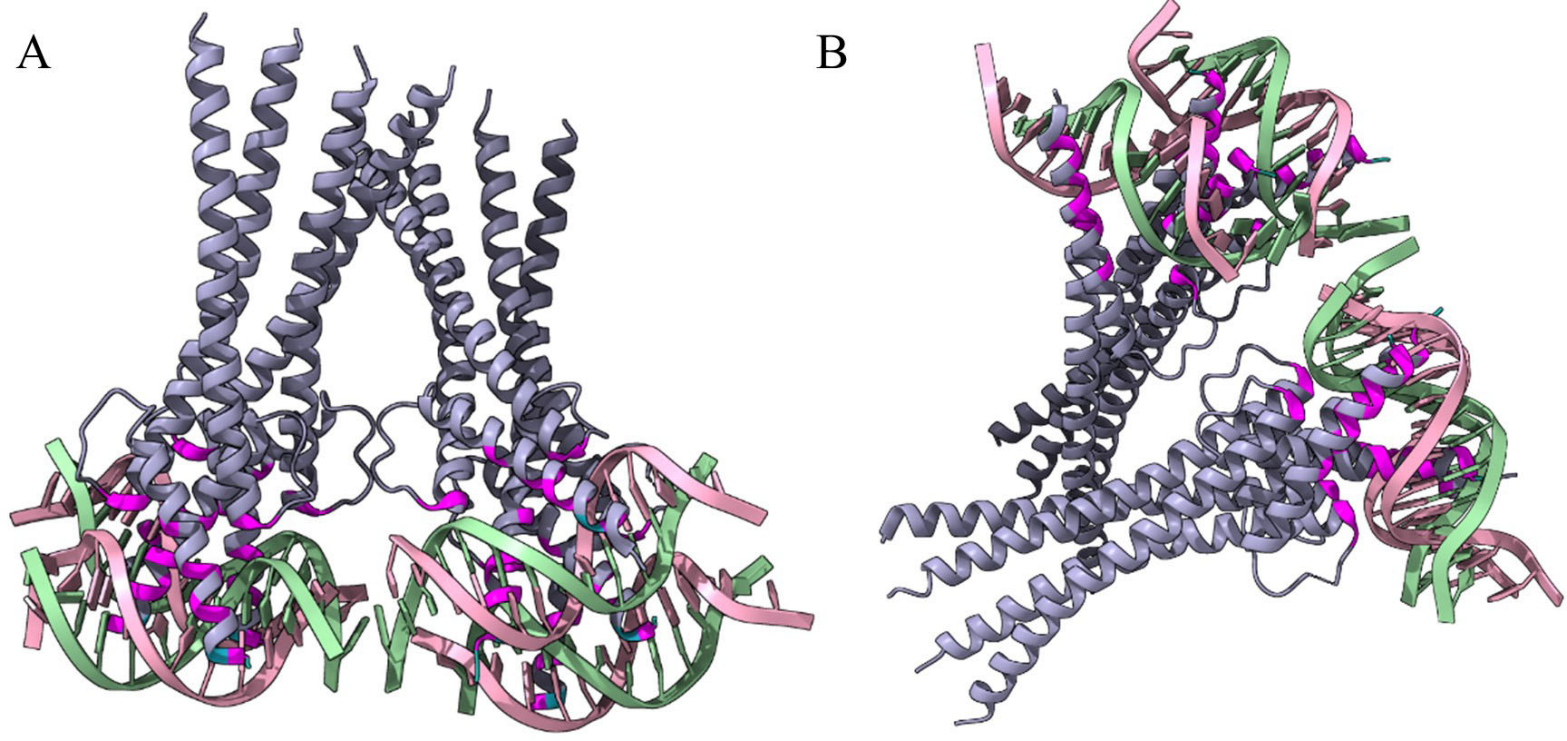
The binding mode of the Arabidopsis MYC2 bHLH (PDB ID: 5GNJ) to the E-box DNA crystal structure. **(A, B)** show the MYC2 bHLH crystal structure around its axis in two different orientations. The magenta-colored residues depict the bHLH-interacting residues with the green- and pink-colored DNA structure. The purple color represents the backbone structure of the MYC2 bHLH crystal structure.

**Figure 3:**
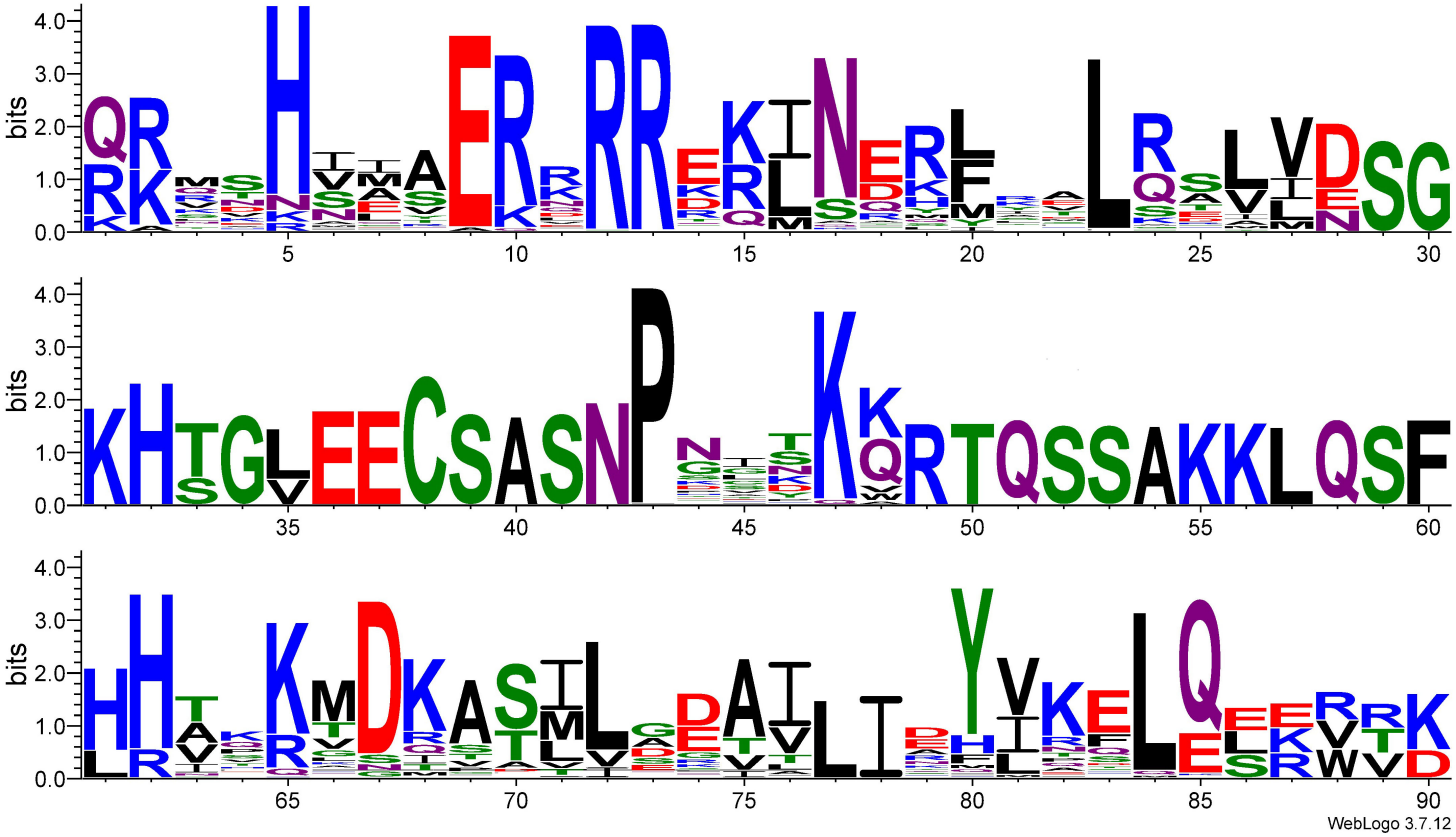
WebLogo analysis of the 377 bHLH domain sequences in the wheat genome. Graphical representation of the multiple sequence alignments of bHLH transcription factor domains identified in wheat. Black, hydrophobic amino acid; green, polar amino acid; blue, positively charged amino acid; red, negatively charged amino acid; purple, neutral amino acid. Bits, conservation at that amino acid position.

The approximately 60 amino acids long bHLH domain contains a highly conserved (bHLH) motif that recognizes the E-box element in the target gene, thus binding the transcription factor to DNA. A search in the PDB database using the protein sequence of bHLH (PF00010) was performed, which resulted in 85 structures for the bHLH domain. An X-ray diffraction of the Arabidopsis (*Arabidopsis thaliana*) bHLH domain, MYC2 bHLH, in complex with DNA, was identified among the structures (PDB ID: 5GNJ, **Figures 2A, B**). The DNA-binding site was identified for 5GNJ from the PDBsum database which was mapped to the identified bHLH domain sequences from this study. Nine residues (histidine, valine, glutamic acid, arginine at three positions, asparagine, aspartic acid and lysine) were identified as interacting with the DNA sequence. Two of the arginine residues at positions 12 and 13 (**Figure 3**) were conserved in all the bHLH sequences. The WebLogo for the identified bHLH sequences shows a conserved histidine residue (H, position five), a highly conserved glutamic acid (E, position nine), arginine (R) at three positions of which two are highly conserved (**Figure 3**). In addition, a proline (P) residue and leucine (L) residues at two positions are highly conserved (**Figure 3**). The full multiple sequence alignment file with the bHLH sequences is available in **Supplementary Figure 1**.

### 3.4 Chromosomal location of bHLH transcription factors

**Figure 4:**
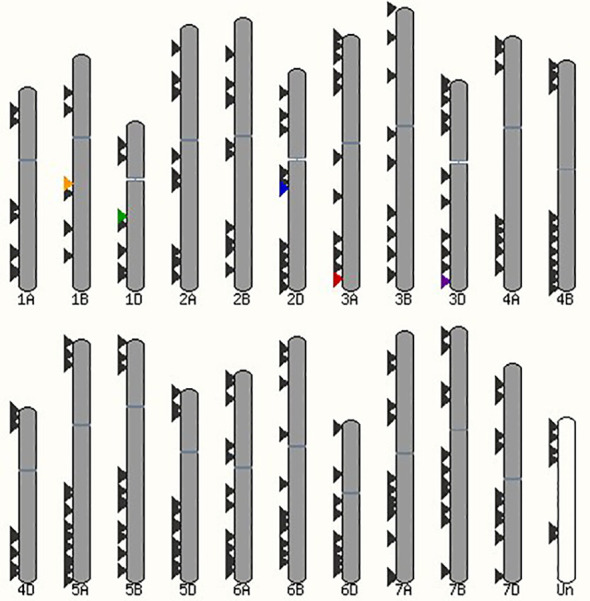
Distribution of all the identified bHLH transcription factors in the wheat genome. The five bHLH transcription factors associated with salt tolerance are highlighted across the seven chromosomes of the A, B and D genomes in the Chinese Spring wheat. Orange, TaMYC2-B (TraesCS1B02G208000); green, TaMYC2-D (TraesCS1D02G196900); blue, FIT1 (TraesCS2D02G280100); red, ORG2-like (TraesCS3A02G489500); purple, ORG2*-like (TraesCS3D02G495400).

All the identified bHLH transcription factors, as well as the five selected bHLH transcription factors, *TaMYC2-B*, *TaMYC2-D*, *FIT1*, *ORG2*-like and ORG2*-like, were mapped on the wheat chromosomes to find their distribution across the wheat genome. *TaMYC2-B* and *TaMYC2-D* were located on chromosomes 1B and 1D, respectively (**Figure 4**). *FIT1*, *ORG2*-like and ORG2*-like were located on chromosomes 2D, 3A and 3D, respectively (Figure. 4). The allocation of the rest of the bHLH transcription factors was evenly distributed across the seven chromosomes (**Figure 4**).

### 3.5 Expression patterns of wheat bHLH transcription factors under NaCl stress

The *in silico* analyzed bHLH transcription factors based on available online public bioproject datasets showed that certain bHLH genes are expressed during salt stress, namely, *TaMYC2-B*, *TaMYC2-D*, *FIT1*, *ORG2*-like and ORG2*-like. To investigate the expression pattern of these five identified bHLH transcription factors in wheat, the gene expression in shoots and roots, and their responses to 100 mM NaCl stress were further analyzed using reverse transcription-quantitative PCR (RT-qPCR). Moreover, the six target genes regulated by the *in silico* upregulated bHLH transcription factors, as well as the six target genes regulated by the *in silico* downregulated bHLH transcription factors involved in wheat salt tolerance, were also scrutinized for their responsiveness to salt stress. These genes were selected based on the best p-values and q-values from the FIMO analysis (Section 2.5).

**Figure 5:**
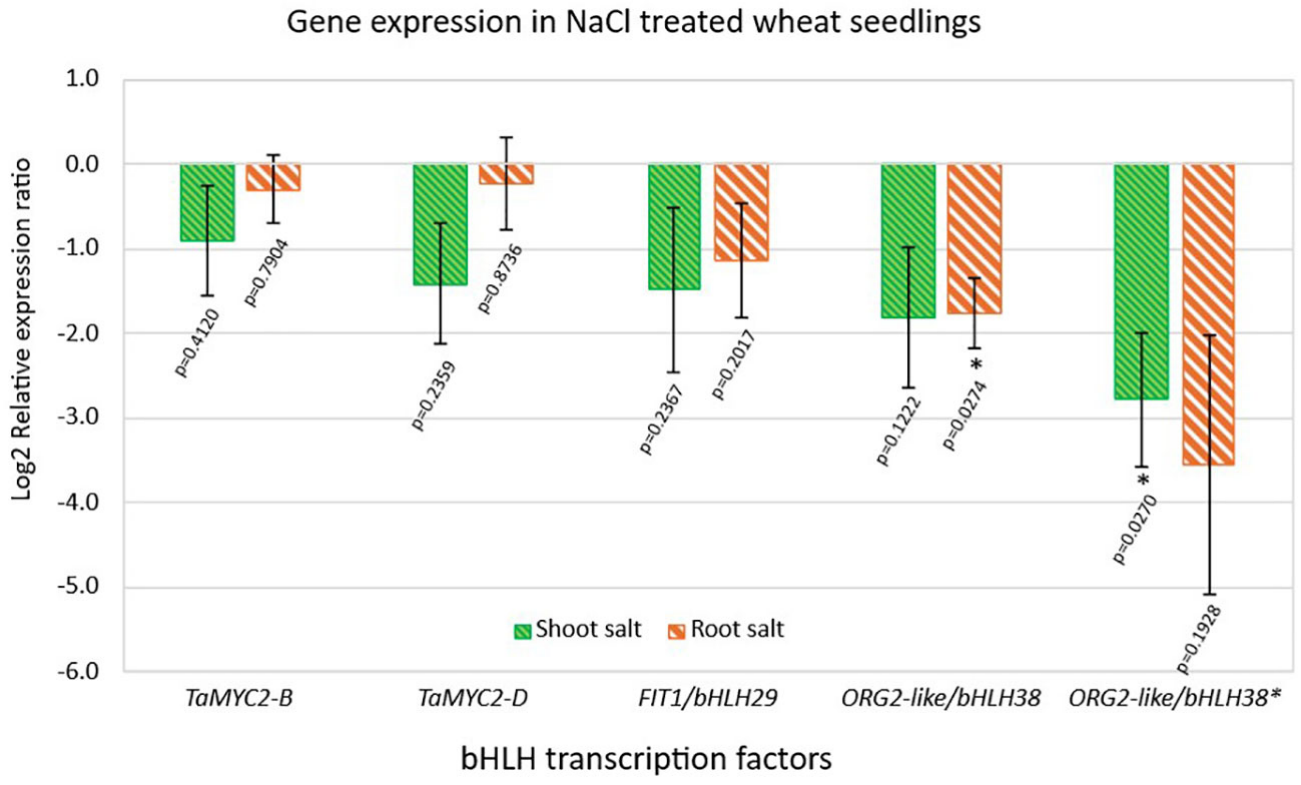
Expression pattern of the five bHLH transcription factors in roots and shoots of the 14-d-old BARI Gom-25 wheat seedlings under 100 mM NaCl treatment. Actin and Glyceraldehyde-3-phosphate dehydrogenase (GAPDH) were used as internal controls. The expression levels of the NaCl treated samples were normalized against the untreated (control) samples. Data are presented as means ± SEM of three biological replicates (n=3). Two-tailed Student’s T-test was performed, and p values are indicated. (*) represents a significant difference between the control plants and the 100 mM NaCl treated plants of p < 0.05*.

The genes encoding the bHLH transcription factors *TaMYC2-B* and *TaMYC2-D*, *FIT1*, *ORG2*-like, and ORG2*-like were all downregulated in NaCl-treated root and shoot tissues of BARI Gom-25 compared with the corresponding untreated control samples (**Figure 5**). Specifically, ORG2*-like exhibited the lowest relative expression levels among the five bHLH transcription factors in NaCl-treated shoot and root tissues (**Figure 5**), with expression values normalized to the corresponding untreated controls. While the *in silico* analysis predicted that *TaMYC2-B*, *TaMYC2-D*, and *FIT1* would be upregulated under salt stress, our RT-qPCR results showed that these transcription factors were downregulated in NaCl-treated samples relative to the corresponding untreated controls. Similarly, the *in silico* analysis predicted that *ORG2*-like and ORG2*-like would be downregulated, which was confirmed by RT-qPCR analysis relative to untreated controls.

### 3.6 Expression patterns of target genes regulated by the *in silico* upregulated bHLH transcription factors

**Figure 6:**
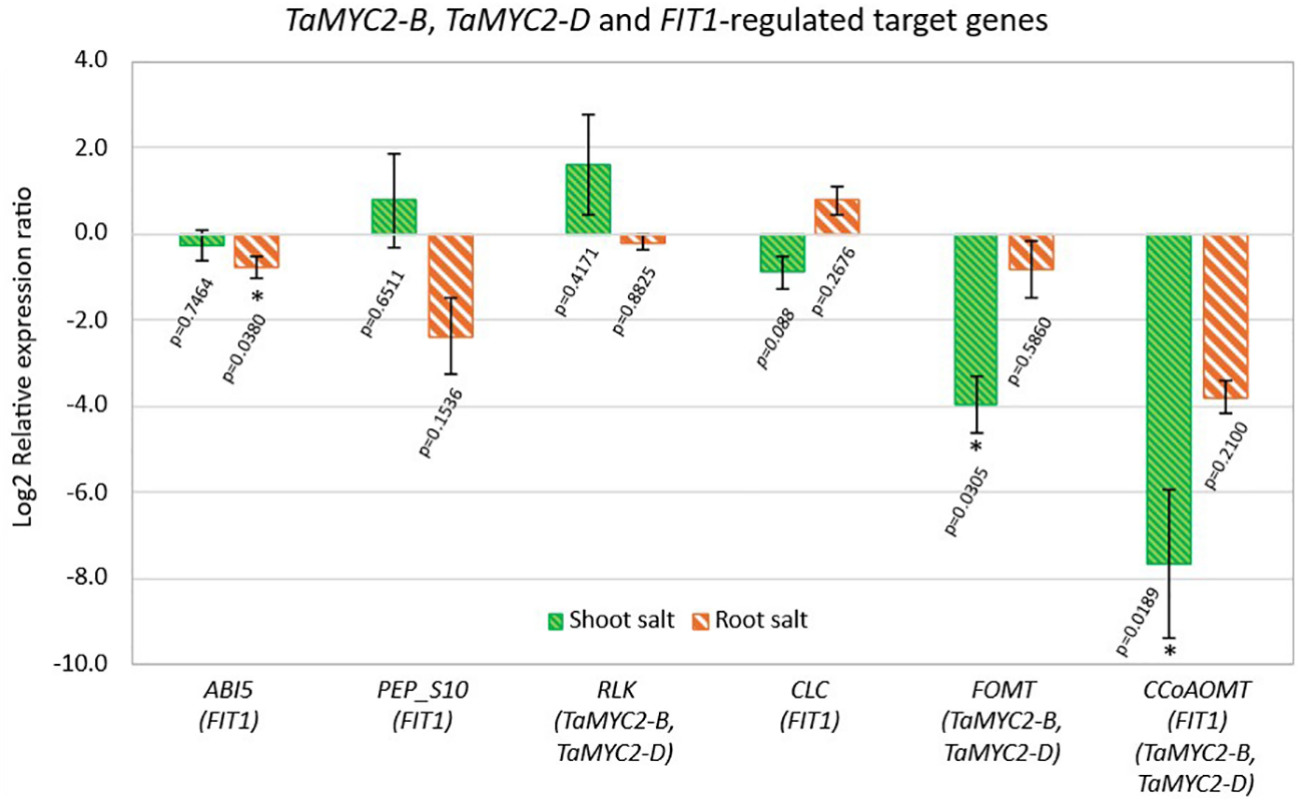
Expression pattern of the bHLH-regulated target genes in the root and shoot of the 14-d-old BARI Gom-25 wheat seedlings under 100 mM NaCl treatment. ABI5 (Abscisic acid-insensitive 5-like protein 5-related); PEP_S10 (Peptidase S10, serine carboxypeptidase); RLK (serine/threonine-protein kinase); CLC (chloride channel protein), FOMT (flavonoid O-methyltransferase) and CCoAOMT (caffeoyl-CoA O-methyltransferase). The names within each bracket in the figure represent the transcription factor(s) regulating that specific target gene. Actin and Glyceraldehyde-3-phosphate dehydrogenase (GAPDH) were used as internal controls. The expression levels of the NaCl treated samples were normalized against the untreated (control) samples. Data are presented as means ± SEM of three biological replicates (n=3). Two-tailed Student’s T-test was performed, and p values are indicated. (*) represents a significant difference between the control plants and the 100 mM NaCl treated plants of p < 0.05*.

Six target genes related to salt stress, regulated by *FIT1*, *TaMYC2-B* and *TaMYC2-D*, were selected for further gene expression studies based on the FIMO analysis; abscisic acid-insensitive 5-like protein 5-related (*ABI5*, TraesCS7A02G170600), Peptidase S10, serine carboxypeptidase (*PEP_S10*, TraesCS5B02G263100), serine/threonine-protein kinase (*RLK*, TraesCS4B02G031100), chloride channel protein (*CLC*, TraesCS2B02G326900), flavonoid *O*-methyltransferase (*FOMT*, TraesCS4D02G073600) and caffeoyl-CoA O-methyltransferase (*CCoAOMT*, TraesCS4A02G442400) (**Figure 6**).

One of the predicted target genes regulated by *FIT1* (bHLH29), *PEP_S10*, was upregulated in NaCl-treated shoots but downregulated in NaCl-treated roots relative to the corresponding untreated controls, whereas *ABI5*, also predicted to be regulated by *FIT1*, was downregulated in both NaCl-treated tissues relative to untreated controls (**Figure 6**). The third predicted *FIT1* target gene, *CLC*, was the only downstream gene showing increased expression in NaCl-treated roots compared with untreated controls (**Figure 6**).

The target genes regulated by *TaMYC2-B* and *TaMYC2-D*, *FOMT* and *RLK*, respectively, exhibited different expression patterns under salt stress. The expression of *RLK* increased in NaCl-treated shoots but decreased in NaCl-treated roots relative to the corresponding untreated controls, whereas the expression of *FOMT* decreased in both tissues under the same conditions (**Figure 6**). *CCoAOMT*, predicted to be regulated by *TaMYC2-B*, *TaMYC2-D* and *FIT1* (bHLH29), exhibited strongly reduced expression in both NaCl-treated root and shoot tissues relative to the corresponding untreated controls (**Figure 6**).

### 3.7 Expression patterns of target genes regulated by the *in silico* downregulated bHLH transcription factors

**Figure 7:**
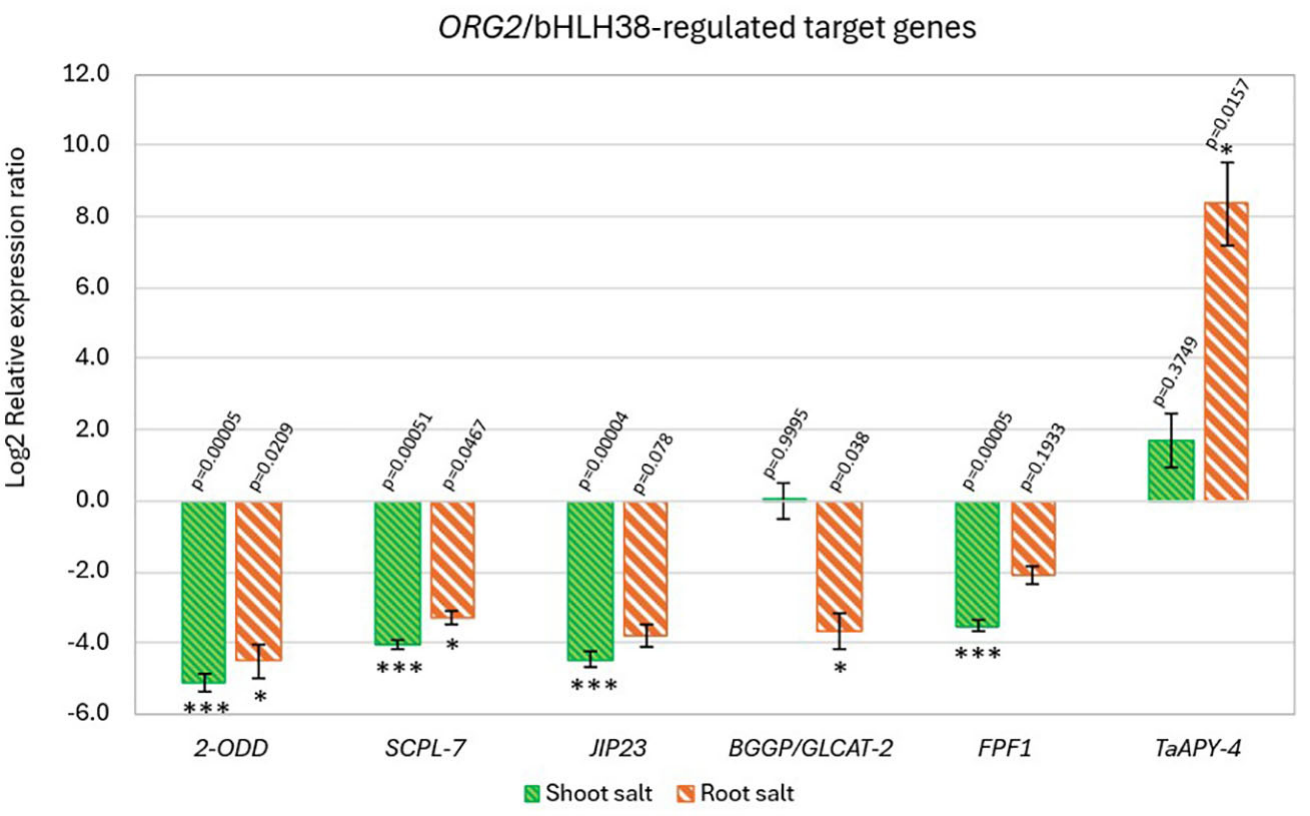
Expression pattern of the ORG2/bHLH38 and ORG2/bHLH38*-regulated target genes in the root and shoot of the 14-d-old BARI Gom-25 wheat seedlings under 100 mM NaCl treatment. 2-ODD (Oxoglutarate/iron-dependent dioxygenase); SCPL-7 (serine carboxypeptidase-like 7); JIP23 (23 kDa jasmonate-induced protein-like); BGGP (β-1-3-galactosyl-O-glycosyl-glycoprotein); FPF1 (flowering-promoting factor 1-like protein 1) and TaAPY (apyrase). Actin and Glyceraldehyde-3-phosphate dehydrogenase (GAPDH) were used as internal controls. The expression levels of the NaCl treated samples were normalized against the untreated (control) samples. Data are presented as means ± SEM of three biological replicates (n=3). Two-tailed Student’s T-test was performed, and p values are indicated. (*, ***) represents a significant difference between the control plants and the 100 mM NaCl treated plants of p < 0.05* or p < 0.0005***.

Six target genes related to salt-stress were selected for further gene expression studies based on the FIMO analysis; oxoglutarate/iron-dependent dioxygenase (*2-ODD*, TraesCS7D02G050500), serine carboxypeptidase-like 7 (*SCPL-7*, TraesCS7B02G021500), 23 kDa jasmonate-induced protein-like (*JIP23*, TraesCS3A02G539100), β-1-3-galactosyl-O-glycosyl-glycoprotein (*BGGP*, TraesCS4B02G061200), flowering-promoting factor 1-like protein 1 (*FPF1*, TraesCS3A02G452300) and apyrase (*TaAPY*, TraesCS5A02G547700) (**Figure 7**). These target genes were all predicted to be regulated by *ORG2*-like (bHLH38, TraesCS3A02G489500) and its paralog bHLH transcription factor ORG2*-like (bHLH38, TraesCS3D02G495400). Under NaCl treatment, the expression levels of *2-ODD*, *SCPL-7*, *JIP23* and *FPF1* decreased in both shoot and root tissues relative to the corresponding untreated controls (**Figure 7**). Following NaCl treatment, *BGGP* expression decreased in root tissues only relative to the corresponding untreated controls, whereas *TaAPY* expression increased in both shoot and root tissues under the same conditions, with the highest fold increase observed in roots (**Figure 7**). The results showed that in roots, all target genes except *TaAPY* were downregulated following treatment with 100 mM NaCl relative to the corresponding untreated controls (**Figure 7**). Specifically, *TaAPY* exhibited high expression levels in both shoot and root tissues under NaCl treatment, whereas *2-ODD*, *SCPL-7*, *JIP23* and *FPF1* were predominantly downregulated compared with untreated controls (**Figure 7**).

### 3.8 Co-expression network analysis reveals bHLH transcription factor modules in salt-stressed wheat

To independently validate the regulatory relationships predicted by FIMO/JASPAR promoter scanning, we constructed a genome-wide co-expression network from the 71 publicly available wheat RNA-seq samples analyzed in this study using Spearman rank correlation. Gene pairs with a correlation coefficient r ≥ 0.7 and a Bonferroni-corrected p-value < 0.01 were retained, yielding a network of 77,500 genes connected by 52,114,726 co-expression edges. Community structure was detected using the Infomap community detection method (Rosvall and Bergstrom, 2008), using 10 trials and undirected flow model, which partitioned the network into 150 top-level modules organized in a four-level hierarchy, reflecting distinct co-regulated gene sets across wheat chromosomes and functional categories.

Infomap community detection assigned *TaMYC2-B*, *TaMYC2-D*, *FIT1*, *ORG2*-like, and ORG2*-like and the 12 candidate target genes to 7 distinct communities within the full co-expression network. Globally, we found that *TaMYC2-B* and *TaMYC2-D* co-localized in community 1:1 together with two candidate target genes, *2-ODD* and *CCoAOMT*. *FIT1* was assigned to community 1:3 alongside the candidate target *RLK*, demonstrating that despite the absence of a direct co-expression edge at the applied threshold, *FIT1* and *RLK* belong to the same co-regulated transcriptional module, which confirms the regulatory relationship predicted by FIMO analysis. In contrast, *ORG2*-like and ORG2*-like were co-assigned to a separate community (1:105) containing neither candidate target genes nor the other manuscript bHLH TFs, suggesting that these two paralogs form an independent co-regulatory module whose transcriptional activity is not broadly captured in the 71-sample dataset.

### 3.9 Subnetwork analysis and bHLH motif enrichment validate predicted TF–target regulatory relationships

To further characterize the regulatory context of the identified co-expression communities, we performed Fisher’s exact test to assess the enrichment of transcription factor binding motifs in the promoters of all genes belonging to each community containing candidate target genes. Across all four communities analyzed (1:1, 1:3, 1:4, and 1:5), the same bHLH subfamilies bHLH77, bHLH66, bHLH31, bHLH69, and bHLH13 were consistently and significantly enriched (Benjamini-Hochberg adjusted p-value ≤ 0.01), indicating that bHLH binding motifs are a defining regulatory feature of the co-expression modules harboring the candidate target genes. Community 1:5, which contains the candidate targets *PEP_S10* and *CLC*, is a community displaying a positive log2 fold-change for multiple bHLH subfamilies members, meaning that the promoters of genes in this community are proportionally enriched in bHLH binding sites (**Supplementary Table 3**).

**Figure 8:**
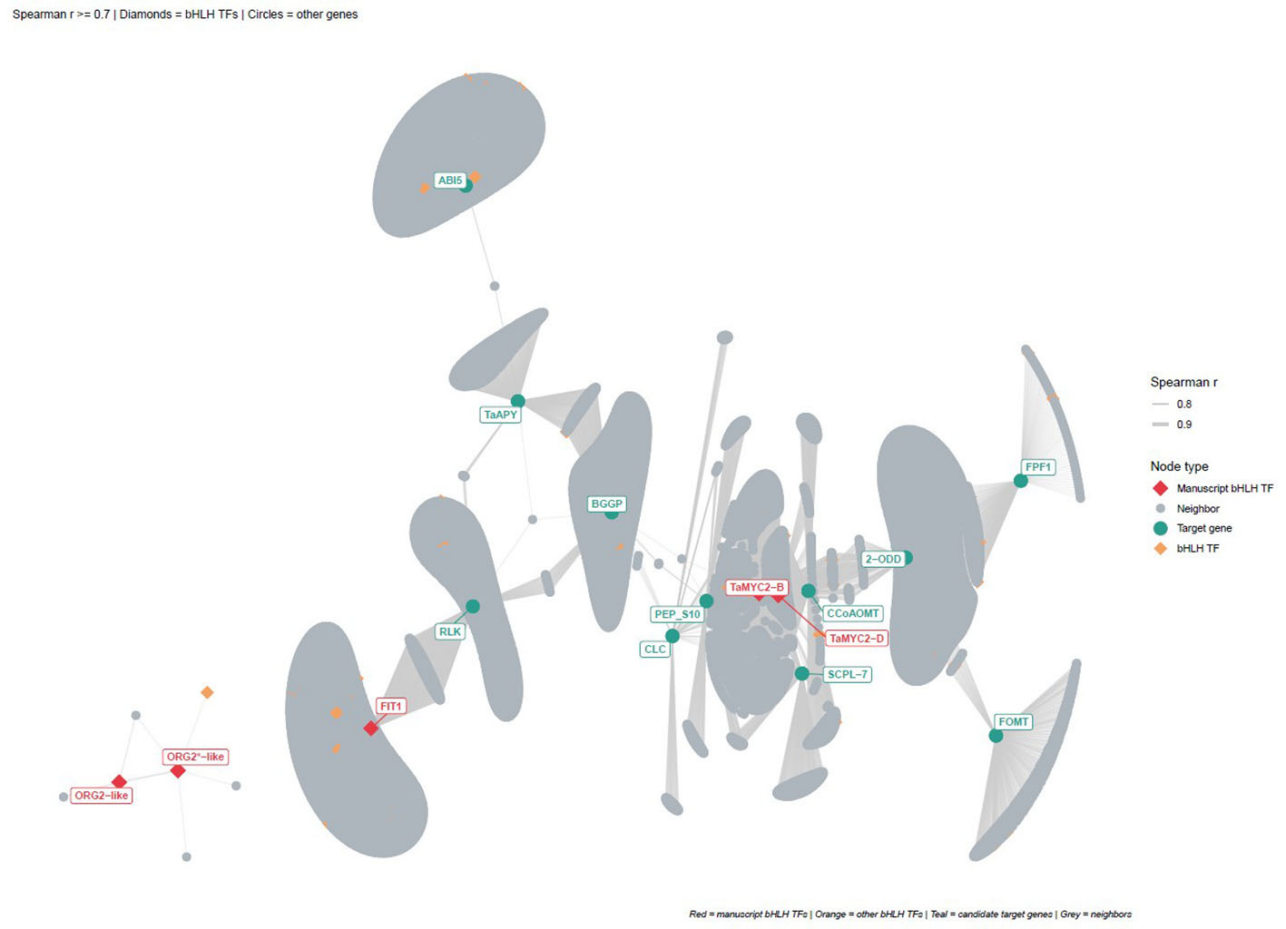
Co-expression subnetwork extracted from a genome-wide co-expression network (77,500 genes; 52,114,726 edges; Spearman r ≥ 0.7, Bonferroni-corrected p <0.01) built from 71 publicly available RNA-seq samples. The subnetwork includes candidate downstream target genes and bHLH transcription factors (TaMYC2-B, TaMYC2-D, FIT1, ORG2-like, and ORG2*-like) together with all genes directly co-expressed with any of these anchor nodes. Node shapes indicate gene category: diamonds represent bHLH transcription factors; circles represent target genes and co-expressed neighbor nodes. Node colors indicate node type: red, manuscript bHLH TFs; orange, other bHLH TFs co-expressed; teal, candidate target genes; grey, neighbor nodes. Edge width represents Spearman correlation strength. An interactive version of this figure with zoom, node search, and community filtering is available through **Supplementary Figure 3**. Community assignments were obtained by applying the Infomap algorithm to the full co-expression network and are indicated by node color grouping in the interactive version.

To represent the co-expression relationships between the five bHLH transcription factors (*TaMYC2-B*, *TaMYC2-D*, *FIT1*, *ORG2*-like, and ORG2*-like) and the 12 candidate downstream target genes identified through FIMO/JASPAR promoter scanning, we extracted a subnetwork using both gene sets, retaining all genes directly co-expressed with any anchor node. The resulting subnetwork comprised 10,629 nodes and 14,169 edges, and included 54 bHLH transcription factors co-expressed with the anchor genes (**Figure 8**). All five manuscript bHLH TFs were present in the full co-expression network; however, only *TaMYC2-B* and *TaMYC2-D* showed direct co-expression with any of the 12 candidate target genes at the applied threshold, while *FIT1*, *ORG2*-like, and ORG2*-like were incorporated into the subnetwork through their own co-expression neighborhoods. Of the 12 candidate target genes, 11 were recovered in the network; *JIP23* (TraesCS3A02G539100) had no co-expressed partners passing the Bonferroni-corrected threshold and was therefore absent from the visualization. *ORG2*-like and ORG2*-like showed a markedly limited co-expression neighborhood (3 and 5 direct neighbors, respectively), in contrast to *TaMYC2-B* (3,715 neighbors), *TaMYC2-D* (3,903 neighbors), and *FIT1* (2,590 neighbors), suggesting that *ORG2*-like and ORG2*-like may exhibit condition-specific or low-level expression patterns in the sampled RNA-seq dataset. Taken together, these results provide independent co-expression evidence supporting the regulatory roles of the manuscript bHLH TFs predicted by FIMO promoter scanning, and place the candidate target genes within defined co-regulated modules of the wheat transcriptome.

## 4 Discussion

### 4.1 DNA binding mode of bHLH transcription factors

In plants, the basic region of a bHLH is represented by the first 10 to 19 amino acids (Carretero-Paulet et al., 2010; Radani et al., 2023; Thoben and Pucker, 2023). The amino acid conservativity in this region determines the DNA binding behavior, such as E-box binding with the presence of glutamic acid and arginine at the positions 9 and 12 (Zhang et al., 2020; Thoben and Pucker, 2023). Consistent with previous reports, our multiple sequence alignment of 377 wheat bHLH domains revealed conserved glutamic acid and arginine at positions nine and twelve (**Figure 3**), respectively, confirming the canonical E-box–binding configuration. The glutamic acid at position nine contacts the first two bases (CA) of the E-box, and this interaction is stabilized by the arginine residue at position 12 which orientates the side chain of the glutamic acid while simultaneously contacting the DNA backbone (Thoben and Pucker, 2023). The WebLogo analysis further showed that most bHLHs—including *TaMYC2-B*, *TaMYC2-D*, *FIT1*, and both *ORG2*-like paralogs—possess the signature residues associated with G-box recognition (**Figure 3**). G-box binders are identified by histidine, glutamic acid and arginine at the positions five, nine, and 13. The histidine residue binds to the last base (G) of the motif. The arginine residue at position 13 distinguishes the G-box from the E-box motif CAGCTG (Ferré-D'Amaré et al., 1993; Shimizu, 1997). Thus, these observations are in agreement with earlier structural studies indicating that histidine, glutamic acid, and arginine at key positions enable high-affinity binding to CACGTG motifs.

Although these results do not establish binding experimentally, they provide a structural basis for predicting potential downstream regulatory targets. In particular, the conservation of G-box specificity across the wheat bHLH family supports the feasibility of the in silico promoter-binding predictions used in this work. Further studies such as electrophoretic mobility shift assays (EMSA) or yeast one-hybrid assays will be needed to confirm whether individual bHLHs bind these motifs under salt stress.

### 4.2 *TaMYC2-B*, *TaMYC2-D* and *FIT1*-regulated target genes

Both *TaMYC2* paralogs displayed reduced transcript levels after prolonged salt exposure (**Figure 5**), consistent with temporal fluctuations reported for *MYC2* orthologs in other species. Similar results in Caucasian clover (*Trifolium ambiguum* M. Bieb.) have been reported where *TaMYC2* expression was significantly lower in plants treated with NaCl for 48 hours compared with untreated controls (Zhao et al., 2022). In the same study, *TaMYC2* expression fluctuated across different time points, with reduced transcript levels observed in stems at most time points except 12 h, and significantly lower expression detected in leaves after 48 hours of NaCl exposure (Zhao et al., 2022). In the present study, gene expression was assessed after 7 days of exposure to 100 mM NaCl to evaluate transcriptional responses under prolonged salinity conditions rather than transient early stress signals. *TaMYC2* transcripts were reduced relative to controls, with a more pronounced downregulation observed in shoots compared with roots, although this difference was not statistically significant. The observed downregulation after 7 days of exposure may reflect later-stage stress responses, as shorter treatments in other systems show dynamic, time-dependent *MYC2* expression. These patterns suggest that *MYC2* activity in wheat may be both tissue-dependent and temporally regulated, although additional time-course analyses would be required to clarify these dynamics. To facilitate such analyses, we recently developed and validated a high-throughput Hyperplex assay platform for large-scale gene expression profiling (Viitanen et al., 2026), which enables reliable analysis of expanded sample sets and is currently being applied in ongoing time-course investigations of these candidate genes.

MYC2, a bHLH transcription factor, is a master regulator which controls a wide range of responses including biotic, abiotic, developmental pathways, signaling pathways such as light, jasmonic acid, auxin, ethylene, and gibberellic acid (Gangappa et al., 2013; Kazan and Manners, 2013; Verma et al., 2020). Although MYC2 has been reported to be involved in such broad range of functions, its involvement in salt stress, for example in wheat, has barely been studied. MYC2 has been reported to be activated through phosphorylation through the Mitogen-Activated Protein Kinase Cascade MKK3–MPK6, causing susceptibility toward salt stress in Arabidopsis (Verma et al., 2020). Overexpression of *MYC2* increased the sensitivity toward salt by inhibiting germination and causing retarded root growth, as well as repressing the gene expression of *P5CS1*, a key enzyme in proline biosynthesis (Verma et al., 2020). Using gene expression analysis, biochemical and physiological assays, it was confirmed that MYC2 negatively regulates salt stress tolerance in Arabidopsis (Verma et al., 2020). Our findings cannot determine whether wheat MYC2 orthologs play analogous roles; however, the reduced transcript levels of *TaMYC2-B* and *TaMYC2-D* under salt stress may be consistent with altered regulatory dynamics that could limit potential growth-inhibitory MYC2 functions. Whether these wheat proteins act as repressors or activators under salt stress remains a testable hypothesis requiring functional validation. Several predicted *TaMYC2* target genes, including *RLK*, *FOMT*, and *CCoAOMT*, exhibited reduced transcript levels in both roots and shoots following NaCl treatment relative to untreated controls, with the exception of *RLK*, which showed increased expression in shoots (**Figure 6**).

Although correlation alone cannot confirm transcriptional regulation, the shared expression patterns raise the possibility that *TaMYC2* may influence these genes, directly or indirectly. Whether this influence involves activation, repression, or indirect regulation remains to be determined experimentally.

The downstream target of *TaMYC2*, *RLK*, is a leucine-rich repeat kinase (LRR-RLK), which has been shown to play important roles in modulating plant growth and development during abiotic stress, as well as signal transduction (Rahim et al., 2022; Gandhi and Oelmüller, 2023; Yan et al., 2023). It has been demonstrated that a member of the LRR-RLK family, GhSIF1, is a negative regulator of salt tolerance in cotton (*Gossypium hirsutum*) (Yuan et al., 2018). In addition, an Arabidopsis receptor Serine/Threonine kinase-like protein (At4g18250) was shown to be downregulated in roots under 150 mM NaCl treatment for 6 h (Jiang et al., 2009). Finally, overexpression of the LRR-RLK gene *AtRPK1*, and a rice homolog *OsRPK1*, markedly decreased salt tolerance of Arabidopsis (Shi et al., 2013). When the expression of the *AtRPK1* gene was inhibited by RNAi, salt tolerance in Arabidopsis improved through increased expression of the *P5CS1* gene (Shi et al., 2013). P5CS1 is a key enzyme in the synthesis of proline in plants, and an increase in its expression can significantly increase the proline content in plants, thus improving salt stress tolerance (Shi et al., 2013; Nguyen et al., 2021). It has also been demonstrated that over-expression of *RPK1* increases cell membrane permeability, thus increasing intracellular Na^+^ flux when plants were exposed to salt stress (Shi et al., 2013).

LRR-RLKs contain three functional domains: an extracellular domain, a single membrane-spanning domain, and an intracellular kinase domain (Liu et al., 2017; Jose et al., 2020). The cytosolic kinase domain phosphorylates downstream substrates, thereby regulating processes such as the transcription of genes involved in proline biosynthesis and osmoprotectant production that contribute to salinity tolerance (Fàbregas et al., 2018; Soltabayeva et al., 2022). Our results indicate that salt stress reduced *RLK* expression in wheat roots while increasing it in wheat shoots relative to the corresponding untreated controls (**Figure 6**). The mixed expression response of *RLK*, a leucine-rich repeat receptor-like kinase, highlights the complexity of RLK functions in salt stress. Nevertheless, the specific roles of wheat LRR-RLKs remain poorly defined, and functional assays will be essential for determining whether the *RLK* identified here participates in salt-induced signaling or contributes to tissue-specific responses.

Lignin is the second most abundant component of the plant cell wall, and Caffeoyl-coenzyme A O-methyltransferase (CCoAOMT) is the main regulator determining the efficiency of lignin synthesis and composition (Nguyen et al., 2016; Yang et al., 2021). Although it has been characterized in many plants, to date, the importance of the CCoAOMT family in wheat is not well understood (Nguyen et al., 2016). The strong reduction in *CCoAOMT* transcript levels in both tissues (**Figure 6**) may have implications for cell wall remodeling under salt stress, as lignin biosynthesis is tightly linked to stress-induced structural adjustments. Several *CCoAOMTs* have been reported to exhibit decreased transcript levels in wheat under salt stress, with transcript abundance declining as the duration of salt exposure increases (Yang et al., 2021). Strikingly, based on available RNA-seq datasets, the same *CCoAOMT* (TraesCS4A02G442400) was reported to show markedly increased transcript levels in wheat under salt stress; however, details regarding salt concentration and tissue specificity were not provided (Yang et al., 2021). Thus, published studies show divergent expression patterns of *CCoAOMT* genes in wheat under salinity, indicating that gene- and condition-specific factors strongly influence their transcription. Additional comparative analyses across different varieties, tissues, and stress intensities will be required to determine whether *CCoAOMT* suppression represents a consistent feature of wheat salt responses.

Among the *FIT1*-predicted targets, *CLC* was the only gene showing increased transcript levels in NaCl-treated roots relative to untreated controls (**Figure 6**). Chlorine channel (CLC) proteins are highly associated with uptake and transport of anions such as Cl^-^ and NO3^-^ (Liu et al., 2020). The *CLC* gene family members contain three highly conserved regions related to anion selectivity, GxGIPE, GKxGPxxH and PxxGxLF, respectively (Liu et al., 2020). If the second residue in the first region (GxGIPE) is proline, NO3^-^ is preferentially transported, whereas if it is substituted by serine, Cl^-^ is transported (Liu et al., 2020). Based on the amino acid sequence of the identified *CLC*, a serine residue at the first region was identified, thus confirming its involvement in Cl^-^ transport (**Supplementary Figure 2**). The fifth residue of the conserved region III (PxxGxLF) was occupied by valine (**Supplementary Figure 2**), thus exerting CLC channel activity (Liu et al., 2020). A similar CLC, found in Arabidopsis, was shown to be involved in vacuolar Cl^-^ accumulation, osmoregulation and stomatal movement, thereby improving salt tolerance through the regulation of chloride homeostasis (Jossier et al., 2010). Several wheat *CLC* genes have been studied under NaCl stress, and the results indicate that their expression patterns are highly variable and dependent on the duration of salt exposure, unlike in some other species, suggesting complex regulatory control (Mao et al., 2022). In the present study, the identified *CLC* gene showed increased expression in roots under NaCl stress, consistent with the role of roots as the primary site of chloride uptake and early ion sensing during salinity stress. Root-specific or root-enhanced induction of *CLC* genes may therefore indicate roles in chloride transport, sequestration, or compartmentalization that help limit the translocation of toxic ions to aerial tissues. In contrast, in pomegranate (*Punica granatum*), the expression levels of *PgCLC* genes under salt stress were reported to be higher in shoots and lower in roots, indicating an opposite tissue-specific expression pattern compared with the wheat *CLC* identified in this study (**Figure 6**) (Liu et al., 2020). Such interspecies differences highlight the diversity of chloride handling strategies among plants and suggest that the spatial regulation of *CLC* genes may be tailored to species-specific physiological and ecological requirements. Nevertheless, direct physiological measurements of Cl^-^ flux or vacuolar transport capacity will be necessary to validate whether the identified *CLC* contributes functionally to salt tolerance in the wheat cultivar BARI Gom-25.

### 4.3 *ORG2*-like/bHLH38 and *ORG2*-like/bHLH38*-regulated target genes

Both *ORG2*-like paralogs, *ORG2*-like/bHLH38 and ORG2*-like/bHLH38, displayed substantial downregulation under salt stress (**Figure 5**). Specifically, the expression of ORG2*-like was downregulated the most by a decrease of ~ 3.5-fold in the roots, and ~ 2.8-fold in the shoots of the BARI Gom-25 wheat seedlings under salt stress (**Figure 5**). In Arabidopsis, *ORG2*/bHLH38 functions with *FIT1* in iron homeostasis and cadmium tolerance, but its role in salinity responses remains unclear (Wu et al., 2012; Cui et al., 2018). Our co-expression networks and bHLH motif enrichment analyses support these previous findings, as some of the genes co-expressed with both *ORG2*-like paralogs were associated with metal and iron ion activity, such as *protein iron-related transcription factor 2* (TraesCS3B02G549800), a MYC-type bHLH transcription factor similar to *FIT1*, as well as a metal transporter *Nramp1* (TraesCS7D02G324000), involved in cadmium tolerance in wheat (Rasheed et al., 2024).

Some studies have indicated that MYC2 can suppress *FIT* and *ORG2* transcription, potentially linking MYC2 to broader stress-regulatory networks (Cui et al., 2018). Our expression patterns—where *MYC2*, *FIT1*, and *ORG2*-like genes all decrease under salt—align with this possibility, though causation cannot be inferred without functional data. The co-expression networks and bHLH motif enrichment analysis likewise suggested that another MYC-type transcription factor (TraesCS3B02G549800), similar to *FIT1*, may be co-expressed with both *ORG2*-like bHLH transcription factors identified in this study, along with several genes associated with metal and iron transport (**Figure 8**, **Supplementary Figure 3**).

The downstream targets of the two *ORG2*/bHLH38 paralog transcription factors—including *2-ODD*, *SCPL-7*, *JIP23*, *BGGP*, *FPF1*, and *TaAPY*—showed distinct expression responses (**Figure 7**). Several of these genes, including *2-ODD*, *SCPL-7, JIP23 and FPF1*, respectively, were all downregulated in both shoots and roots of the BARI Gom-25 wheat seedlings (**Figure 7**). While their precise contributions to salt adaptation in wheat remain largely unknown, their expression trends indicate that ORG2-like networks may be broadly suppressed under chronic salt stress.

Serine carboxypeptidase-like proteins (SCPLs) belong to a large family of enzymes that hydrolyze proteins and play roles in multiple cellular processes (Liu et al., 2008; Chen et al., 2020; Xu et al., 2021). Available evidence in plants suggests that SCPLs have various functions, such as signal transduction, seed development and mobilization of storage proteins, yet their function in salinity tolerance is lacking (Liu et al., 2008; Chen et al., 2020; Xu et al., 2021). It has been shown that *SCPL-7* was downregulated in wheat when exposed to drought stress, similar to our reported results under salt stress (**Figure 7**) (Xu et al., 2021). Only *BGGP* showed a tissue-specific reduction in roots (**Figure 7**), suggesting potential involvement in cell wall carbohydrate remodeling, given its role in arabinogalactan biosynthesis. *BGGP*, or beta-glucuronosyltransferase GlcAT14A/B/C, is an enzyme belonging to glycosyltransferase family 14 (Knoch et al., 2013; Dilokpimol and Geshi, 2014; Ajayi and Showalter, 2020). They are involved in the biosynthesis of type II arabinogalactan (AG), a glycoprotein located on cell surfaces of plants (Dilokpimol and Geshi, 2014; Ajayi and Showalter, 2020; Ajayi et al., 2021). It was reported that an Arabidopsis β–glucuronosyltransferase, GlcAT14A, enhances cell elongation during seedling growth, and the enzymes may play a role in intracellular Ca^2+^ signaling (Knoch et al., 2013). Our results show that the expression levels of *BGGP* remained unchanged in the shoots of BARI Gom-25 under salt stress during the seedling stage (**Figure 7**), an important developmental phase when plants form leaves. However, insufficient data exist to relate changes in *BGGP* expression to salt tolerance mechanisms.

Among all targets, *TaAPY* exhibited the most striking induction, particularly in roots by an ~ 8-fold increase (**Figure 7**). Apyrases (APYs), a class of nucleoside triphosphate diphosphohydrolases (NTPDases), play an important role in maintaining NTP homeostasis (Roux and Steinebrunner, 2007; Liu et al., 2019; Clark et al., 2024). Abiotic stresses can significantly elevate extra-cellular ATP (eATP) levels, which further lead to growth inhibition, initiation of cell death and apoptosis (Liu et al., 2019). These negative effects that elevated eATP can cause on plant growth may, however, be countered by hydrolysis by apyrases (Roux and Steinebrunner, 2007; Clark et al., 2024). Unlike ATPases that use Mg^2+^ as a co-factor, APYs can use a variety of divalent co-factors, including Ca^2+^, Mg^2+^, Mn^2+^ and Zn^2+^ (Liu et al., 2019). The same *TaAPY* as investigated in this study, has also been reported to be involved in wheat salt tolerance (Liu et al., 2019). In the same study, it was also demonstrated that Ca^2+^ was the most effective cofactor and that *TaAPY* exhibited high enzymatic activity toward ATP compared to TTP, GTP and CTP, suggesting a strong substrate preference for ATP (Liu et al., 2019). Conclusively, these previously reported results showed that *TaAPY* preferentially hydrolyzes ATP in a Ca^2+^-dependent manner, supporting its potential involvement in salt-induced ATP signaling pathways. We thereby hypothesize that *TaAPY* could play an important role in enhancing salt tolerance in wheat through the cleavage of elevated eATP levels.

While our findings suggest that ORG2-like bHLHs may influence *TaAPY* expression, direct regulatory relationships would require promoter binding confirmation or reporter assays.

## 5 Conclusion

This study explored the potential regulatory roles of five in silico–identified bHLH transcription factors in wheat under salt stress, integrating sequence analyses, functional inference, and preliminary gene expression profiling. Two paralogous MYC2 transcription factors, *TaMYC2-B* and *TaMYC2-D*, were identified as candidate MYC2 master regulators in wheat. Several studies have reported that MYC2 contributes to susceptibility to salt stress by inhibiting germination and root growth, as well as repressing proline biosynthesis (Verma et al., 2020; Zhao et al., 2022). In our work, both *TaMYC2* paralogs showed reduced transcript levels under salt stress, raising the possibility that *MYC2* downregulation may contribute to the moderate salt tolerance observed in the BARI Gom-25 wheat variety. However, this interpretation remains speculative, as reduced *TaMYC2* expression does not necessarily equate to enhanced salt tolerance without direct functional or physiological validation. The mechanistic role of MYC2 in wheat salt stress responses therefore remains to be clarified.

Among the additional bHLH transcription factors assessed, *FIT1* and the *ORG2*-like/bHLH38 paralogs showed salt-responsive expression patterns. One predicted *FIT1* target, a chloride channel protein (*CLC*), exhibited increased expression in roots under salt stress in BARI Gom-25. Chloride channels (CLCs) are associated with uptake and transport of anions such as Cl^-^ and NO3^-^ (Liu et al., 2020). Sequence characteristics and amino acid substitutions of this CLC suggest Cl^-^ specificity, consistent with roles in chloride transport and homeostasis (**Supplementary Figure 2**). CLCs maintain chloride homeostasis through the accumulation of Cl^-^ ions in the vacuole, osmoregulation and stomatal movement (Jossier et al., 2010; Liu et al., 2020). While our findings are consistent with the hypothesis that this CLC may support vacuolar Cl^-^ sequestration or osmotic adjustment under salinity, experimental validation will be required to determine its functional contribution to salt tolerance in wheat.

We also show that both *ORG2*-like/bHLH38 paralogs were downregulated under salt stress. It has been reported that *ORG2*/bHLH38 interacts with *FIT* to improve iron homeostasis in Arabidopsis (Wu et al., 2012; Cui et al., 2018). Furthermore, the expression of both *ORG2*/bHLH38 and *FIT* can be repressed by the master regulator MYC2 (Cui et al., 2018; Kanwar et al., 2021). However, the roles of these transcription factors in wheat salinity responses are largely unknown. The regulatory relationships among *MYC2*, *FIT1*, and *ORG2*-like transcription factors in wheat thus remain an important topic for future investigation.

Finally, this study highlights the potential importance of apyrases in salt stress responses. Apyrases (APYs) are known to play crucial roles in abiotic stress responses, primarily through alleviating the adverse effects of elevated extra-cellular ATP (eATP) levels (Roux and Steinebrunner, 2007; Liu et al., 2019; Clark et al., 2024).

*TaAPY*, previously linked to Ca^2+^-mediated stress signaling and shown to enhance salt tolerance in wheat (Liu et al., 2019), exhibited strong upregulation in roots under salt stress (**Figure 7**). Given that extracellular ATP (eATP) accumulates during abiotic stress and can inhibit growth, increased *TaAPY* expression may help mitigate these effects by cleaving elevated eATP levels in the roots, the primary site of salt stress-induced responses. Although our results suggest a possible link between apyrase activity and the moderate salt tolerance of BARI Gom-25, establishing this relationship will require physiological and functional analyses.

Overall, this study identifies a set of salt-responsive bHLH transcription factors and associated candidate genes that may contribute to salinity tolerance in wheat. While the putative regulatory relationships presented here are based on *in silico* promoter analyses, co-expression networks, bHLH motif enrichment analyses, and preliminary expression data, they provide a focused set of hypotheses for future functional investigation. These findings establish a useful framework for advancing our understanding of bHLH-mediated salt stress responses in wheat. From a translational perspective, the candidate genes identified in this study represent promising targets for wheat improvement following functional validation, including the development of molecular markers for marker-assisted selection or prioritization for gene-editing approaches aimed at enhancing salt tolerance. Collectively, these insights refine our understanding of the molecular basis of wheat salt-stress responses and highlight candidate targets that may ultimately support the development of salt-resilient cultivars.

## Data Availability

The original contributions presented in the study are included in the article/**Supplementary Material**, further inquiries can be directed to the corresponding author/s.

## Supplementary material

Supplementary Figure 1 Multiple sequence alignment with the 377 bHLH sequences.

Supplementary Figure 2 Multiple sequence alignment of the CLC proteins.

Supplementary Figure 3 An HTML version of the Interactive co-expression network is provided as a ZIP, which can be downloaded and viewed locally in a web browser.

Supplementary Figure 4 Venn diagram showing the overlap of differentially expressed genes across the RNA-seq dataset.

Supplementary Table 1 Primers used for RT-qPCR.

Supplementary Table 2 RNA-seq sample metadata.

Supplementary Table 3 Transcription factor enrichment analysis.
