# Supplementary figures and images for: Genome-wide identification and expression analysis of salt-responsive bHLH transcription factors in the wheat (Triticum aestivum) genome
Source: Front Plant Sci. 2026 Apr 23;17:1770759. doi: 10.3389/fpls.2026.1770759 (PMC13151688; doi:10.3389/fpls.2026.1770759)

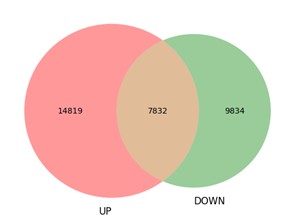

Supplement: Supplementary Figure 1 — Multiple sequence alignment with the 377 bHLH sequences. [file Image1.jpeg]
