# Supplementary material for: Genome-wide identification and expression analysis of salt-responsive bHLH transcription factors in the wheat (Triticum aestivum) genome
Source: Front Plant Sci. 2026 Apr 23;17:1770759. doi: 10.3389/fpls.2026.1770759 (PMC13151688; doi:10.3389/fpls.2026.1770759)
Supplement: Supplementary Figure 2 — Multiple sequence alignment of the CLC proteins. [file DataSheet1.pdf]

Reference sequence (1): TraesCS6B02G244800.1#domain1#PF00010
Identities normalised by aligned length.
Colored by: identity

```
                                                cov    pid   1 [        .         .         .         .         :         .         .        . 80
 1 TraesCS6B02G244800.1#domain1#PF00010        100.0% 100.0%  ----RSIAERERRTRISDKLRKLQDLV--------------PNMDKQ-----------------TSTSDMLDLAV--EH
 2 TraesCS6A02G214900.1#domain1#PF00010        100.0% 100.0%  ----RSIAERERRTRISDKLRKLQDLV--------------PNMDKQ-----------------TSTSDMLDLAV--EH
 3 TraesCS6D02G197500.1#domain1#PF00010        100.0%  97.9%  ----RSIAERERRTRISYKLRKLQDLV--------------PNMDKQ-----------------TSTSDMLDLAV--EH
 4 TraesCS3D02G388700.1#domain1#PF00010        100.0%  83.0%  ----RSIAERERRTRISKRLRRLQDLV--------------PNMDKQ-----------------TNTSDMLDIAV--DY
 5 TraesCS3B02G426900.1#domain1#PF00010        100.0%  83.0%  ----RSIAERERRTRISKRLRRLQDLV--------------PNMDKQ-----------------TNTSDMLDIAV--DY
 6 TraesCS3A02G395000.1#domain1#PF00010        100.0%  83.0%  ----RSIAERERRTRISKRLRRLQDLV--------------PNMDKQ-----------------TNTSDMLDIAV--DY
 7 TraesCS7A02G245800.1#domain1#PF00010        100.0%  78.7%  ----RSIAERVRRTRISERIRKLQELV--------------PNMDKQ-----------------TNTADMLDLAV--DY
 8 TraesCS7D02G244300.1#domain1#PF00010        100.0%  78.7%  ----RSIAERVRRTRISERIRKLQELV--------------PNMDKQ-----------------TNTADMLDLAV--DY
 9 TraesCS7B02G145800.1#domain1#PF00010        100.0%  78.7%  ----RSIAERVRRTRISERIRKLQELV--------------PNMDKQ-----------------TNTADMLDLAV--DY
10 TraesCS5A02G264800.1#domain1#PF00010        100.0%  76.6%  ----RSIAERVRRTKISERIRKLQELV--------------PNMEKQ-----------------TNTSDMLDLAV--DY
11 TraesCS5D02G272800.1#domain1#PF00010        100.0%  76.6%  ----RSIAERVRRTKISERIRKLQELV--------------PNMEKQ-----------------TNTSDMLDLAV--DY
12 TraesCS5B02G264300.1#domain1#PF00010        100.0%  76.6%  ----RSIAERVRRTKISERIRKLQELV--------------PNMEKQ-----------------TNTSDMLDLAV--DY
13 TraesCS4B02G317900.1#domain1#PF00010         97.9%  37.5%  ---THNDTEKRRRCKISDRLRTLQQLV--------------PGCKK------------------CNQASTLEQTI--QY
14 TraesCS3B02G010400.1#domain1#PF00010         93.6%  44.4%  ------AVAKVRRERISERLKVLQDLV--------------PNGTK------------------VDMVTMLEKAI--TY
15 TraesCS3B02G007700.1#domain1#PF00010         93.6%  44.4%  ------AVAKVRRERISERLKVLQDLV--------------PNGTK------------------VDMVTMLEKAI--TY
16 TraesCS3D02G004300.1#domain1#PF00010         93.6%  44.4%  ------AVAKVRRERISERLKVLQDLV--------------PNGTK------------------VDMVTMLEKAI--TY
17 TraesCS3A02G006300.1#domain1#PF00010         87.2%  47.6%  ---------KVRRERISERLKVLQDLV--------------PNGTK------------------VDMVTMLEKAI--TY
18 TraesCS6A02G276200.1#domain1#PF00010         87.2%  45.2%  ---------KTRREKISERLRTLQELV--------------PNGTK------------------VDMVTMLEKAF--SY
19 TraesCS6B02G303600.1#domain1#PF00010         89.4%  46.5%  --------AKNRREKISERLRTLQELV--------------PNGTK------------------VDMVTMLEKAV--SY
20 TraesCS6D02G256600.1#domain1#PF00010         89.4%  46.5%  --------AKNRREKISERLRTLQELV--------------PNGTK------------------VDMVTMLEKAV--SY
21 TraesCS6D02G256400.1#domain1#PF00010         89.4%  44.2%  --------AKNRREKISERLRTLQELV--------------PNGTK------------------VDMVTMLEKAF--SY
22 TraesCS6D02G256500.1#domain1#PF00010         87.2%  45.2%  ---------KNRREKISERLRTLQELV--------------PNGTK------------------VDMVTMLEKAF--SY
23 TraesCS6A02G276100.1#domain1#PF00010         87.2%  45.2%  ---------KNRREKISERLRTLQELV--------------PNGTK------------------VDMVTMLEKAF--SY
24 TraesCS6B02G303500.1#domain1#PF00010         87.2%  45.2%  ---------KNRREKISERLRTLQELV--------------PNGTK------------------VDMVTMLEKAF--SY
25 TraesCS6D02G256300.1#domain1#PF00010         87.2%  45.2%  ---------KNRREKISERLRTLQELV--------------PNGTK------------------VDMVTMLEKAF--SY
26 TraesCS4B02G257200.1#domain1#PF00010         87.2%  45.2%  ---------RKRRQKINERLKVLQKLI--------------PNGTK------------------VDISTMLEEAV--HY
27 TraesCS4A02G047700.1#domain1#PF00010         87.2%  45.2%  ---------RKRREKINERLKVLQNLI--------------PNGTK------------------VDISTMLEEAV--HY
28 TraesCS4D02G257100.1#domain1#PF00010         87.2%  45.2%  ---------RKRREKINERLKVLQNLI--------------PNGTK------------------VDISTMLEEAV--HY
29 TraesCS5A02G067600.1#domain1#PF00010         87.2%  50.0%  ---------RKRRERINERLKTLQTLV--------------PNGTK------------------VDMSTMLEEAV--QY
30 TraesCS5D02G078800.1#domain1#PF00010         87.2%  50.0%  ---------RKRRERINERLKTLQTLV--------------PNGTK------------------VDMSTMLEEAV--QY
31 TraesCS5B02G074500.1#domain1#PF00010         87.2%  52.4%  ---------RKRRKRINERLKTLQTLV--------------PNGTK------------------VDMSTMLEEAV--QH
32 TraesCS2A02G194200.1#domain1#PF00010         87.2%  50.0%  ---------RKRRERINERLKTLQNLV--------------PNGTK------------------VDISTMLEEAV--EY
33 TraesCS2B02G212700.1#domain1#PF00010         87.2%  50.0%  ---------RKRRERINERLKTLQNLV--------------PNGTK------------------VDISTMLEEAV--EY
34 TraesCS2D02G193700.1#domain1#PF00010         87.2%  50.0%  ---------RKRRERINERLKTLQNLV--------------PNGTK------------------VDISTMLEEAV--EY
35 TraesCS4A02G234500.1#domain1#PF00010         87.2%  50.0%  ---------RKRREKINERLKVLQNLV--------------PNGTK------------------VDISTMLEEAV--EY
36 TraesCS4D02G079500.1#domain1#PF00010         87.2%  50.0%  ---------RKRREKINERLKVLQNLV--------------PNGTK------------------VDISTMLEEAV--EY
37 TraesCS4B02G080700.1#domain1#PF00010         87.2%  50.0%  ---------RKRREKINERLKVLQNLV--------------PNGTK------------------VDISTMLEEAV--EY
38 TraesCS5B02G406100.1#domain1#PF00010         87.2%  52.4%  ---------KRRRERINDRLRVLQKLV--------------PNGTK------------------VDLSTMLEEAV--LY
39 TraesCS5D02G411600.1#domain1#PF00010         87.2%  50.0%  ---------KRRREKINDRLRALQKLV--------------PNGTK------------------VDLSTMLEEAV--LY
40 TraesCS5A02G401300.1#domain1#PF00010         87.2%  50.0%  ---------KRRREKINDRLRVLQKLV--------------PNGTK------------------VDLSTMLEEAV--LY
41 TraesCS1B02G359000.1#domain1#PF00010         85.1%  43.9%  ---------KRRRHKINERLRVLQHLI--------------PNGTK------------------VDISTMLEEAV--QY
42 TraesCS1A02G345200.1#domain1#PF00010         85.1%  43.9%  ---------KRRRHKINERLRVLQHLI--------------PNGTK------------------VDISTMLEEAV--QY
43 TraesCS1D02G347900.1#domain1#PF00010         85.1%  43.9%  ---------KRRRHKINERLRVLQHLI--------------PNGTK------------------VDISTMLEEAV--HY
44 TraesCS3A02G440600.1#domain1#PF00010         89.4%  48.8%  --------AKRRRERINEKLRELQNLI--------------PNGTK------------------VDISTMLEEAV--QY
45 TraesCS3D02G433200.1#domain1#PF00010         89.4%  48.8%  --------AKRRRERINEKLRVLQRLI--------------PNGTK------------------VDISTMLEEAV--QY
46 TraesCS3B02G474700.1#domain1#PF00010         87.2%  50.0%  ---------KRRRERINEKLRVLQHLI--------------PNGTK------------------VDISTMLEEAV--QY
47 TraesCS3A02G350600.1.cds1#domain1#PF00010   85.1%  48.8%  ---------RERRHRISDRFRVLRSLV--------------PGGSK------------------MDTVSMLEQAI--HY
48 TraesCS3B02G383000.1.cds1#domain1#PF00010   85.1%  48.8%  ---------RERRHRISDRFRVLRSLV--------------PGGSK------------------MDTVSMLEQAI--HY
49 TraesCS3D02G344600.1.cds1#domain1#PF00010   85.1%  48.8%  ---------RERRHRISDRFRVLRSLV--------------PGGSK------------------MDTVSMLEQAI--HY
50 TraesCS7B02G160500.1.cds1#domain1#PF00010   87.2%  21.7%  ---------RMRRERISERIRILQRLV--------------PGGTK------------------MDTASMLDEAI--HY
51 TraesCS7A02G340300.1#domain1#PF00010        100.0%  32.0%  -RSKHSATEQRRRCKINERFQILRDLV--------------PQSDQK-----------------RDKATFLLEVI--EY
52 TraesCS7B02G251900.1#domain1#PF00010        100.0%  32.0%  -RSKHSATEQRRRCKINERFQILRDLV--------------PQSDQK-----------------RDKATFLLEVI--EY
53 TraesCS7D02G347900.1#domain1#PF00010        100.0%  32.0%  -RSKHSATEQRRRCKINERFQILRDLV--------------PQSDQK-----------------RDKATFLLEVI--EY
54 TraesCS1B02G226200.2#domain1#PF00010         97.9%  46.8%  ----HNESERKRRDRINQKMQTLQKLV--------------PNSSK------------------TDKASMLDEVI--DH
55 TraesCS1D02G215600.2#domain1#PF00010         97.9%  46.8%  ----HNESERKRRDRINQKMQTLQKLV--------------PNSSK------------------TDKASMLDEVI--DH
56 TraesCS1A02G212700.1#domain1#PF00010         97.9%  46.8%  ----HNESERKRRDRINQKMQTLQKLV--------------PNSSK------------------TDKASMLDEVI--DH
57 TraesCS2D02G461900.1#domain1#PF00010         97.9%  42.6%  ----HNFSERRRRDKINEKLKALQELL--------------PNCTK------------------TDKVSMLDEVI--DY
58 TraesCS2D02G461700.3#domain1#PF00010         97.9%  42.9%  ----HNFSERRRRDKINEKLKALQELL--------------PNCTK----------------VHTDKVSMLDEAI--DY
59 TraesCS2A02G461700.2#domain1#PF00010         97.9%  42.9%  ----HNFSERRRRDKINEKLKALQELL--------------PNCTK----------------VHTDKVSMLDEAI--DY
60 TraesCS2B02G483300.1#domain1#PF00010         97.9%  42.9%  ----HNFSERRRRDKINEKLKALQELL--------------PNCTK----------------VHTDKVSMLDEAI--DY
61 TraesCS1B02G100400.2#domain1#PF00010         97.9%  43.8%  ---VHNMSERRRRDRINEKMRALQELI--------------PNCNK------------------IDKASMLEEAI--EY
62 TraesCS1A02G083000.1#domain1#PF00010         97.9%  43.8%  ---VHNMSERRRRDRINEKMRALQELI--------------PNCNK------------------IDKASMLEEAI--EY
63 TraesCS1D02G084200.1#domain1#PF00010         97.9%  43.8%  ---VHNMSERRRRDRINEKMRALQELI--------------PNCNK------------------IDKASMLEEAI--EY
64 TraesCS5B02G054800.2#domain1#PF00010         97.9%  46.9%  --QVHNLSERRRRDRINEKMRALQELV--------------PHCNK------------------TDKASMLDEAI--EY
65 TraesCS5A02G049600.1#domain1#PF00010         97.9%  46.9%  --QVHNLSERRRRDRINEKMRALQELV--------------PHCNK------------------TDKASMLDEAI--EY
66 TraesCS5D02G060300.1#domain1#PF00010         97.9%  46.9%  --QVHNLSERRRRDRINEKMRALQELV--------------PHCNK------------------TDKASMLDEAI--EY
67 TraesCS5A02G376500.1#domain1#PF00010         97.9%  45.8%  ---VHNLSERRRRDRINEKMRALQELI--------------PHCNK------------------TDKASMLDEAI--EY
68 TraesCS5B02G380200.1#domain1#PF00010         97.9%  45.8%  ---VHNLSERRRRDRINEKMRALQELI--------------PHCNK------------------TDKASMLDEAI--EY
69 TraesCS5D02G386500.1#domain1#PF00010         97.9%  45.8%  ---VHNLSERRRRDRINEKMRALQELI--------------PHCNK------------------TDKASMLDEAI--EY
70 TraesCS2A02G253900.2#domain1#PF00010         97.9%  41.7%  ---VHNQSERKRRDRINEKMKALQELV--------------PHCNK------------------SDKASILDEAI--EY
71 TraesCS2B02G273500.1#domain1#PF00010         97.9%  41.7%  ---VHNQSERKRRDRINEKMKALQELV--------------PHCNK------------------SDKASILDEAI--EY
72 TraesCS2D02G254400.3#domain1#PF00010         97.9%  41.7%  ---VHNQSERKRRDRINEKMKALQELV--------------PHCNK------------------SDKASILDEAI--EY
73 TraesCS5A02G420200.1#domain1#PF00010         97.9%  41.7%  ---VHNQSERRRRDRINEKMRSLQELI--------------PHCNK------------------ADKASILDEAI--EY
74 TraesCS5B02G422000.1#domain1#PF00010         97.9%  41.7%  ---VHNQSERRRRDRINEKMRSLQELI--------------PHCNK------------------ADKASILDEAI--EY
75 TraesCS5D02G428400.2#domain1#PF00010         97.9%  41.7%  ---VHNQSERRRRDRINEKMRSLQELI--------------PHCNK------------------ADKASILDEAI--EY
76 TraesCS6B02G411300.1#domain1#PF00010         97.9%  41.7%  ---VHNLSEKRRRCKINEKMKALQSLV--------------PDSSK------------------TDKASMLDDAI--EY
77 TraesCS6A02G373500.2#domain1#PF00010         97.9%  43.8%  ---VHNLSEKRRRCKINEKMKALQSLV--------------PNSSK------------------TDKASMLDDAI--EY
78 TraesCS6D02G357700.1#domain1#PF00010         97.9%  43.8%  ---VHNLSEKRRRCKINEKMKALQSLV--------------PNSSK------------------TDKASMLDDAI--EY
79 TraesCS7A02G126900.1#domain1#PF00010         97.9%  43.8%  ---VHNLSEKRRRSRINEKMKALQSLI--------------PNSNK------------------TDKASMLDEAI--EY
80 TraesCS7D02G124700.1#domain1#PF00010         97.9%  43.8%  ---VHNLSEKRRRSRINEKMKALQSLI--------------PNSNK------------------TDKASMLDEAI--EY
81 TraesCS7B02G026300.1#domain1#PF00010         97.9%  43.8%  ---VHNLSEKRRRSRINEKMKALQSLI--------------PNSNK------------------TDKASMLDEAI--EY
82 TraesCS6A02G190600.1#domain1#PF00010         95.7%  42.9%  ---VHNLSERRRRSRINEKMKALQILI--------------PNSNK------------------TDKASMLDEAILIEY
83 TraesCS7A02G323000.1.cds1#domain1#PF00010   85.1%  51.2%  ---------RHRRERISERIRILQRLV--------------PGGTK------------------MDTASMLDEAI--RY
84 TraesCS7D02G319600.1.cds1#domain1#PF00010   85.1%  51.2%  ---------RHRRERISERIRILQRLV--------------PGGTK------------------MDTASMLDEAI--RY
85 TraesCS7B02G223900.1.cds1#domain1#PF00010   85.1%  51.2%  ---------RHRRERISERIRILQRLV--------------PGGTK------------------MDTASMLDEAI--RY
86 TraesCS7D02G263400.1.cds1#domain1#PF00010   85.1%  48.8%  ---------RMRRERISERIRILQRLV--------------PGGTK------------------MDTASMLDEAI--HY
87 TraesCS7A02G262600.1.cds1#domain1#PF00010   85.1%  48.8%  ---------RMRRERISERIRILQRLV--------------PGGTK------------------MDTASMLDEAI--HY
88 TraesCS7B02G303300.1#domain1#PF00010        100.0%  35.4%  ---SHSLAERARREKINARMELLKELV--------------PGCSKV-----------------SGTALVLEEII--NH
89 TraesCS7D02G397300.1#domain1#PF00010        100.0%  35.4%  ---SHSLAERARREKINARMELLKELV--------------PGCSKV-----------------SGTALVLEEII--NH
90 TraesCS7A02G157700.1#domain1#PF00010         97.9%  51.1%  ----HSIAERLRREKISERMKNLQDLV--------------PNSNK------------------ADKSSMLDEII--DY
91 TraesCS7D02G158300.1#domain1#PF00010         97.9%  51.1%  ----HSIAERLRREKISERMKNLQDLV--------------PNSNK------------------ADKSSMLDEII--DY
```

92	TraesCS7B02G062100.2#domain1#PF00010	97.9%	51.1%	----HSIAERLRREKISERMKNLQDLV---------------PNSNK------------------ADKSSMLDEII--DY
93	TraesCS7A02G147500.1#domain1#PF00010	97.9%	48.9%	----HSIAERLRRERIAERMKSLQELV---------------PNANK------------------TDKASMLDEII--DY
94	TraesCS7D02G149400.1#domain1#PF00010	97.9%	48.9%	----HSIAERLRRERIAERMKSLQELV---------------PNANK------------------TDKASMLDEII--DY
95	TraesCS6B02G391100.1#domain1#PF00010	97.9%	46.8%	----HSIAERLRRERIAERMKALQELV---------------PSANK------------------TDKASMLDEII--DY
96	TraesCS6D02G341200.1#domain1#PF00010	97.9%	46.8%	----HSIAERLRRERIAERMKALQELV---------------PSANK------------------TDKASMLDEII--DY
97	TraesCS6A02G358400.3#domain1#PF00010	97.9%	48.9%	----HSIAERLRRERIAERMKALQELV---------------PNANK------------------TDKASMLDEII--DY
98	TraesCS5A02G225700.1#domain1#PF00010	97.9%	48.9%	----HSIAERLRRERIAERMKALQELV---------------PNANK------------------TDKASMLDEII--DY
99	TraesCS5D02G233000.1#domain1#PF00010	100.0%	46.9%	----HSIAERLRRERIAERMKALQELV---------------PNANKW---------------LQTDKASMLDEII--DY
100	TraesCS5B02G224200.1#domain1#PF00010	100.0%	46.9%	----HSIAERLRRERIAERMKALQELV---------------PNANKW---------------LQTDKASMLDEII--DY
101	TraesCS5D02G300100.1#domain1#PF00010	97.9%	40.8%	---SHSLSERVRRERISERMRMLQSLV---------------PGCD-K---------------V-TGKALILDEII--NY
102	TraesCS5B02G292100.1#domain1#PF00010	97.9%	40.8%	---SHSLSERVRRERISERMRMLQSLV---------------PGCD-K---------------V-TGKALILDEII--NY
103	TraesCS5D02G459800.1#domain1#PF00010	97.9%	32.7%	---SHSLAERARREKITERMKILQDLV---------------PGCN-K---------------V-IGKASVLDEII--NY
104	TraesCS5B02G457600.1#domain1#PF00010	97.9%	32.7%	---SHSLAERARREKITERMKILQDLV---------------PGCN-K---------------V-IGKASVLDEII--NY
105	TraesCS4D02G237100.3#domain1#PF00010	100.0%	40.0%	--NNHSLAERFRREKINERMKHLQDLV---------------PGCNKK---------------I-TGKAMMLDEII--NY
106	TraesCS4B02G235700.1#domain1#PF00010	97.9%	38.0%	--NNHSLAERFRREKINERMKHLQDLV---------------PGCN-K---------------I-TGKAMMLDEII--NY
107	TraesCS4A02G060200.2#domain1#PF00010	97.9%	38.0%	--NNHSLAERFRREKINERMKHLQDLV---------------PGCN-K---------------I-TGKAMMLDEII--NY
108	TraesCS4A02G255800.3#domain1#PF00010	97.9%	42.0%	--NSHSLAERLRRKKISEKMKLLQDLV---------------PGCN-K---------------I-TGKAVMLDEII--NY
109	TraesCS4D02G058900.1#domain1#PF00010	97.9%	40.0%	--NSHSLAERLRRKKISERMKLLQDLV---------------PGCN-K---------------I-TGKAVMLDEII--NY
110	TraesCS4B02G059000.1#domain1#PF00010	97.9%	40.0%	--NSHSLAERLRRKKISERMKLLQDLV---------------PGCN-K---------------I-TGKAVMLDEII--NY
111	TraesCS7D02G327900.1#domain1#PF00010	95.7%	24.5%	-KTAHITVERNRRKQMNEHLTVLRSLM---------------PCFY----------------VKRGDQASIIGGVV--DY
112	TraesCS7A02G350900.1#domain1#PF00010	95.7%	24.5%	-KTAHITVERNRRKQMNEHLTVLRSLM---------------PCFY----------------VKRGDQASIIGGVV--DY
113	TraesCS7B02G232000.1#domain1#PF00010	95.7%	24.5%	-KTAHITVERNRRKQMNEHLTVLRSLM---------------PCFY----------------VKRGDQASIIGGVV--DY
114	TraesCS4B02G347100.1#domain1#PF00010	95.7%	22.2%	QRMTHIAVERNRRRLMNDHLASLRALI---------------PSDY----------------IPRGDQATVVGGAI--DY
115	TraesCS5A02G515800.1#domain1#PF00010	95.7%	22.2%	QRMTHIAVERNRRRLMNDHLASLRALI---------------PSDY----------------IPRGDQATVVGGAI--DY
116	TraesCS4D02G342000.1#domain1#PF00010	95.7%	22.2%	QRMTHIAVERNRRRLMNDHLASLRALI---------------PSDY----------------IPRGDQATVVGGAI--DY
117	TraesCS2B02G141600.1#domain1#PF00010	93.6%	24.5%	RKMSHIAVERNRRRQMNDHLKVLRALT---------------PAFY----------------IKRCDQASIIGGAI--EF
118	TraesCS2D02G122800.1#domain1#PF00010	93.6%	26.0%	---SHIAVERNRRRQMNDHLKVLRALT---------------PAFY----------------IKRCDQASIIGGAI--EF
119	TraesCS2A02G120700.1#domain1#PF00010	93.6%	26.0%	---SHIAVERNRRRQMNDHLKVLRALT---------------PAFY----------------IKRCDQASIIGGAI--EF
120	TraesCS6B02G346100.1#domain1#PF00010	95.7%	20.4%	QRRNHIAVERNRRRQMNDYLAVLRSAM---------------PPSY----------------AQRGDQASIVAGAI--NF
121	TraesCS6D02G294900.1#domain1#PF00010	95.7%	20.4%	QRRNHIAVERNRRRQMNDYLAVLRSAM---------------PPSY----------------AQRGDQASIVAGAI--NF
122	TraesCS6A02G315700.1#domain1#PF00010	95.7%	20.4%	QRRNHIAVERNRRRQMNDYLAVLRSSM---------------PPSY----------------AQRGDQASIVAGAI--NF
123	TraesCS1B02G300100.1#domain1#PF00010	95.7%	25.9%	QRMTHIAVERNRRRQMNDYLRVLRSLM---------------PGSY----------------VQRGDQASIIGGAI--EF
124	TraesCS1D02G289400.1#domain1#PF00010	95.7%	25.9%	QRMTHIAVERNRRRQMNDYLRVLRSLM---------------PGSY----------------VQRGDQASIIGGAI--EF
125	TraesCS1A02G290800.1#domain1#PF00010	95.7%	25.9%	QRMTHIAVERNRRRQMNDYLRVLRSLM---------------PGSY----------------VQRGDQASIIGGAI--EF
126	TraesCS6D02G315200.1#domain1#PF00010	95.7%	20.4%	QRMTHIAVERNRRKQMNEYLGVLRSLM---------------PASY----------------VHRGDQASIIGGAI--NY
127	TraesCS6A02G335500.1#domain1#PF00010	95.7%	20.4%	QRMTHIAVERNRRKQMNEYLGVLRSLM---------------PASY----------------VHRGDQASIIGGAI--NY
128	TraesCS6B02G366200.1#domain1#PF00010	95.7%	20.4%	QRLTHIAVERNRRRQMNEYLGVLRSLM---------------PASY----------------VHRGDQASIIGGAI--NY
129	TraesCS6A02G264200.1#domain1#PF00010	95.7%	20.4%	QRMTHIAVERNRRRQMNEYLVVLRSLM---------------PESY----------------VQRGDQASIVGGAI--DF
130	TraesCS6B02G291200.1#domain1#PF00010	95.7%	20.4%	QRMTHIAVERNRRRQMNEYLVVLRSLM---------------PESY----------------VQRGDQASIVGGAI--DF
131	TraesCS6D02G250700.1#domain1#PF00010	95.7%	20.4%	QRMTHIAVERNRRRQMNEYLVVLRSLM---------------PESY----------------VQRGDQASIVGGAI--DF
132	TraesCS2B02G469700.1#domain1#PF00010	95.7%	20.4%	QRMTHIAVERNRRRQMNEYLAALRSLM---------------PESY----------------AHRGDQASIVGGAI--DF
133	TraesCS2B02G469800.1#domain1#PF00010	95.7%	20.4%	QRMTHIAVERNRRRQMNEYLAALRSLM---------------PESY----------------AHRGDQASIVGGAI--DF
134	TraesCS2D02G448000.1#domain1#PF00010	95.7%	20.4%	QRMTHIAVERNRRRQMNEYLAALRSLM---------------PESY----------------AHRGDQASIVGGAI--DF
135	TraesCS2A02G448800.1#domain1#PF00010	95.7%	20.4%	QRMTHIAVERNRRRQMNEYLAVLRSLM---------------PESY----------------AHRGDQASIVGGAI--DF
136	TraesCS4B02G304500.1#domain1#PF00010	95.7%	20.4%	QRRTHIAVERNRRRQMNEYLAALRSLM---------------PQSY----------------AQRGDQASIVGGAI--NY
137	TraesCS4A02G404700.1#domain1#PF00010	95.7%	20.4%	QRRTHIAVERNRRRQMNEYLAALRSLM---------------PQSY----------------AQRGDQASIVGGAI--NY
138	TraesCS4D02G302700.1#domain1#PF00010	95.7%	20.4%	QRRTHIAVERNRRRQMNEYLAALRSLM---------------PQSY----------------AQRGDQASIVGGAI--NY
139	TraesCS5B02G245900.1#domain1#PF00010	95.7%	20.4%	QRMTHIAVERNRRKQMNEYLAALRSLM---------------PPSY----------------AQRGDQASIVGGAI--NF
140	TraesCS5A02G248200.1#domain1#PF00010	95.7%	20.4%	QRMTHIAVERNRRKQMNEYLAALRSLM---------------PPSY----------------AQRGDQASIVGGAI--NF
141	TraesCS5D02G255100.1#domain1#PF00010	95.7%	20.4%	QRMTHIAVERNRRKQMNEYLAALRSLM---------------PPSY----------------ALRGDQASIVGGAI--NF
142	TraesCS4B02G391500.1#domain1#PF00010	95.7%	24.0%	--QEHVIAERKRREKLHLQFVSLAMII---------------PGIK------------------KTDKLSLLGSTI--DY
143	TraesCS5A02G555200.1#domain1#PF00010	95.7%	24.0%	--QEHVIAERKRREKLQQQFVSLATII---------------PGIK------------------KTDKLSLLGSTI--DY
144	TraesCS4B02G345800.1#domain1#PF00010	95.7%	24.0%	--QEHVIAERKRREKLQQQFVSLATII---------------PGIK------------------KTDKLSLLGSTI--DY
145	TraesCS5A02G505100.1#domain1#PF00010	95.7%	26.0%	--QEHVIAERKRREKLQQQFVSLATIV---------------PGLK------------------KTDKLSLLGSTI--DY
146	TraesCS4B02G234700.1#domain1#PF00010	95.7%	26.0%	--QEHVIAERKRREKLQQQFVSLATIV---------------PGLK------------------KTDKISLLGSTI--DY
147	TraesCS4D02G235900.1#domain1#PF00010	95.7%	26.0%	--QEHVIAERKRREKLQQQFVSLATIV---------------PGLK------------------KTDKISLLGSTI--DY
148	TraesCS4B02G335200.1#domain1#PF00010	95.7%	26.0%	--QEHVIAERKRREKLQQQFVSLATIV---------------PGLK------------------KTDKLSLLGSTI--DY
149	TraesCS4D02G330500.1#domain1#PF00010	95.7%	26.0%	--QEHVIAERKRREKLQQQFVSLATIV---------------PGLK------------------KTDKLSLLGSTI--DY
150	TraesCS4B02G056600.2#domain1#PF00010	95.7%	28.0%	--QDHIMAERKRREKLSERFIALSKIV---------------PGLK------------------KMDKASVLGDAI--KY
151	TraesCS4D02G056900.2#domain1#PF00010	95.7%	28.0%	--QDHIMAERKRREKLSERFIALSKIV---------------PGLK------------------KMDKASVLGDAI--KY
152	TraesCS4A02G257900.2#domain1#PF00010	95.7%	28.0%	--QDHILAERRRREKLSERFIALSKIV---------------PGLK------------------KMDKASVLGDAI--KY
153	TraesCS4B02G021100.1#domain1#PF00010	95.7%	28.6%	---DHNMAERKRREKINQRFIELSTVI---------------PGLK------------------KMHKATILSDAT--RH
154	TraesCS4B02G021500.1#domain1#PF00010	95.7%	28.6%	---DHIMAERKRREKINQRFIELSTVI---------------PGLK------------------KMDKATILSDAT--RH
155	TraesCS4D02G019200.1#domain1#PF00010	95.7%	28.6%	---DHIMAERKRREKINQRFIELSTVI---------------PGLK------------------KMDKATILSDAT--RH
156	TraesCS4A02G292800.1#domain1#PF00010	95.7%	28.6%	---DHIMAERKRREKINQRFIELSTVI---------------PGLK------------------KMDKATILSDAT--RH
157	TraesCS4B02G020900.1#domain1#PF00010	95.7%	28.6%	---DHIMAERKRREKINQRFIELSTVI---------------PGLK------------------KMDKATILSDAT--RH
158	TraesCS4B02G021200.1#domain1#PF00010	95.7%	26.5%	---DHIMAERKRREKINQRFIELSTII---------------PGLK------------------KMDKATILSDTT--RH
159	TraesCS4D02G018700.1#domain1#PF00010	95.7%	26.5%	---DHIMAERKRREKINQRFIELSTVI---------------PGLK------------------KMDKATILSDTT--RH
160	TraesCS3A02G019100.1#domain1#PF00010	95.7%	27.1%	----HIMAERKRREKTNQRFIELSALI---------------PGLK------------------KMDKGTILSNAT--SY
161	TraesCS3A02G019000.1#domain1#PF00010	95.7%	27.1%	----HIMAERKRREKTNQRFIELSALI---------------PGLK------------------KMDKGTILSNAT--SY
162	TraesCS3B02G029800.1#domain1#PF00010	95.7%	24.5%	---DHIMAERKRREKTNQRFIELSAVI---------------PGLK------------------KMDKVTILSNAT--SY
163	TraesCS3D02G014900.1#domain1#PF00010	95.7%	24.5%	---DHIMAERKRREKTNQRFIELSAVI---------------PGLK------------------KMDKGTILSNAT--SY
164	TraesCS4A02G408700.1#domain1#PF00010	95.7%	28.6%	---DHIMAERKRRENINQSFIELSALI---------------PGLK------------------KMDKGTILADAT--IF
165	TraesCS4D02G306400.1#domain1#PF00010	95.7%	26.5%	---DHIMAERKRREKINQRFIELSAVI---------------PGLK------------------KMDKGTILADAT--RY
166	TraesCS4B02G308300.1#domain1#PF00010	95.7%	26.5%	---DHIMAERKRREKINQRFIELSAVI---------------PGLK------------------KMDKGTILADAT--RY
167	TraesCS4B02G308200.1#domain1#PF00010	95.7%	26.5%	---DHIMAERKRREKINQRFIELSAVI---------------PGLK------------------KMDKGTILADAT--RY
168	TraesCS4A02G408800.1#domain1#PF00010	95.7%	26.5%	---DHIMAERKRREKINQRFIELSAVI---------------PGLK------------------KMDKGTILTDAT--RY
169	TraesCS4B02G308100.1#domain1#PF00010	93.6%	28.6%	---DHVMAERKRREKINQRFIELSALI---------------PGL-------------------KMDKGTILTEAT--RY
170	TraesCS4D02G306300.1#domain1#PF00010	93.6%	28.6%	---DHVMAERKRREKINQRFIELSALI---------------PGL-------------------KMDKGTILTDAT--RY
171	TraesCS7B02G483600.1#domain1#PF00010	95.7%	33.3%	----HIIAERRRREKINQRLMELSTLI---------------PGLK------------------KMNKATIIGDAV--KH
172	TraesCS7A02G558700.1#domain1#PF00010	95.7%	33.3%	----HIIAERRRREKINQRLMELSTLI---------------PGLK------------------KMNKATIIGDAV--KH
173	TraesCS5B02G518800.1#domain1#PF00010	95.7%	22.4%	---EHVMAERKRREKLNRHFIEHSTVI---------------PGLK------------------KMDKTTILSDAV--RY
174	TraesCSU02G075100.1#domain1#PF00010	91.5%	22.9%	--QKHVMAERKRREKLNRHFIELSTVI---------------PGLK------------------KMDKTTILSDAV--RY
175	TraesCS5B02G013000.2#domain1#PF00010	95.7%	30.6%	---DHIIAERRRREKINQRFIELSTVI---------------PNLK------------------KMDKATILGDAV--KY
176	TraesCS5D02G020600.1#domain1#PF00010	95.7%	30.6%	---DHIIAERRRREKINQRFIELSTVI---------------PNLK------------------KMDKATILGDAV--KY
177	TraesCS5A02G014800.1#domain1#PF00010	95.7%	30.6%	---DHIIAERRRREKINQRFIELSTVI---------------PNLK------------------KMDKATILGDAV--KY
178	TraesCS4B02G020700.1#domain1#PF00010	95.7%	24.5%	---DHIMAERKRREKINRRFIELSTVI---------------PGLK------------------KMDKATILSDAV--KY
179	TraesCS4D02G018800.2#domain1#PF00010	95.7%	26.5%	---DHIMAERKRREKINRRFIELSTVI---------------PGLK------------------KMDKATILSDAV--KY
180	TraesCS4D02G019100.1#domain1#PF00010	95.7%	26.5%	---DHIMAERKRREKINRRFIELSTVI---------------PGLK------------------KMDKATILSDAV--KY
181	TraesCS4A02G292700.1#domain1#PF00010	95.7%	26.5%	---DHIMAERKRREKINRRFIELSTVI---------------PGLK------------------KMDKATILSDAV--RY
182	TraesCSU02G075200.3#domain1#PF00010	95.7%	24.5%	---EHVMAERKRREKINRRFIELSTVI---------------PGLK------------------RMDKATILSDAL--RC
183	TraesCS5B02G518500.1#domain1#PF00010	95.7%	24.5%	---EHVVAERKRREKINRRFIELSTVI---------------PGLK------------------KMDKATILSDAL--RY
184	TraesCS5B02G518400.1#domain1#PF00010	95.7%	26.0%	--QEHVMAERKRREKINRRFIELSTVI---------------PSLK------------------KMDKATILSDAV--RY
185	TraesCS4A02G354100.1#domain1#PF00010	95.7%	26.0%	--QEHVMAERKRREKINRRFIELSTVI---------------PGLK------------------KMDKATILSDAV--RY
186	TraesCS5D02G517900.1#domain1#PF00010	95.7%	26.0%	--QEHVMAERKRREKINRRHFIELSTVI---------------PGLK------------------KMDKATILSDAV--RY
187	TraesCS7A02G421000.1#domain1#PF00010	95.7%	25.5%	KKLSHNAYERDRRTQLNQLYSTLRSLI---------------PNAD---------------HTKKLSIPTTVCQVL--DY
188	TraesCS7B02G321400.1#domain1#PF00010	95.7%	25.5%	KKLNHNAYERDRRTQLNQLYSTLRSLI---------------PNAD---------------HTKKLSIPTTVCQVL--DY
189	TraesCS3A02G489400.1#domain1#PF00010	95.7%	19.6%	RKMSHSAYERDSRKQLNEHYSNLRSLL---------------PDDD--------------HHTRKLNNPTIVSRVI--KY
190	TraesCS3B02G549700.1#domain1#PF00010	95.7%	19.6%	RKMSHSAYERDSRKQLNEHYSNLRSLL---------------PDDD--------------HHTRKLGNPTIVSRVI--KY

191	TraesCS3D02G495300.1#domain1#PF00010	95.7%	19.6%	RKMSHSAYERDSRKQLNEHYSNLRSLL---------------PDDD--------------HHTRKLGNPTIVSRVI--KY
192	TraesCS3A02G489500.1#domain1#PF00010	95.7%	21.8%	RKMSHNAYERDRRKQLNEQYSSLRSLL---------------PDDD---------------HNKKMSIPTTVSRVI--KY
193	TraesCS3D02G495400.1#domain1#PF00010	95.7%	21.8%	RKMSHNAYERDRRKQLNEQYSSLRSLL---------------PDDD---------------HNKKMSIPTTVSRVI--KY
194	TraesCS3B02G549800.1#domain1#PF00010	95.7%	21.8%	RKMSHNAYERDRRKQLNEQYSSLRSLL---------------PDDD---------------HTKKMSIPTTVSRVI--NY
195	TraesCS2B02G543700.1#domain1#PF00010	95.7%	23.6%	KKLSHNAYERDRRKQLNELYLSLRSLL---------------PDAD---------------HTKKLSIPTTVCRAL--KY
196	TraesCS2B02G543800.1#domain1#PF00010	95.7%	23.6%	KKLSHNAYERDRRKQLNELYLSLRSLL---------------PDAD---------------HTKKLSIPTTVCRAL--KY
197	TraesCS2D02G517000.1#domain1#PF00010	95.7%	23.6%	KKLSHNAYERDRRKQLNELYLSLRSLL---------------PDAD---------------HTKKLSIPTTVCRAL--KY
198	TraesCS2A02G515300.1#domain1#PF00010	95.7%	23.6%	KKLSHNAYERDRRKQLNELYLSLRSLL---------------PDTD---------------HTKKLSIPTTVCRAL--KY
199	TraesCS3B02G550200.2#domain1#PF00010	95.7%	21.8%	RKISHNAYERDRRKQLNELYSDLRSLL---------------PDTD---------------HTKKLSIPITVSRVL--KY
200	TraesCS3D02G495700.1#domain1#PF00010	95.7%	21.8%	RKISHNAYERDRRKQLNELYSDLRSLL---------------PDTD---------------HTKKLSIPITVSRVL--KY
201	TraesCS3A02G489700.1#domain1#PF00010	95.7%	21.8%	RKISHNAYERDRRKQLNELYSDLRSLL---------------PDTD---------------HTKKLSIPITVSRVL--KY
202	TraesCS3B02G550000.1#domain1#PF00010	95.7%	21.8%	RKISHNAYERDRRKELNELYSDLRSLL---------------PEDD---------------RTKKLSIPITVSRVL--KY
203	TraesCS3D02G495600.1#domain1#PF00010	95.7%	21.8%	RKISHNAYERDRRKELNELYSDLRSLL---------------PEDD---------------RTKKLSIPITVSRVL--KY
204	TraesCS3A02G489600.1#domain1#PF00010	93.6%	18.5%	RKISHNAYERDRRKELNELYSDLRSLL---------------PEDD---------------RTKKLSIPITVSRVL--KY
205	TraesCS6A02G114100.1#domain1#PF00010	95.7%	32.7%	-RELHIITERERRRRMSEMFTKLHGLL---------------PTLP-----------------DKVDKSSIVMEAI--HY
206	TraesCS6B02G142000.1#domain1#PF00010	95.7%	32.7%	-RELHIITERERRRRMSEMFTKLHGLL---------------PTLP-----------------DKVDKSSIVMEAI--HY
207	TraesCS2A02G442500.1#domain1#PF00010	87.2%	24.4%	------ANERQRREWMNVAFWALRILL---------------PD-P-----------------K-DDRVSNVEDAI--EY
208	TraesCS6D02G246100.2#domain1#PF00010	91.5%	26.0%	--KANFATEKERREQINVKYGALRSLL---------------PS-P-----------------TKNDRASIVGDAI--EY
209	TraesCS6B02G295900.2#domain1#PF00010	91.5%	26.0%	--KANFATEKERREQINVKYGALRSLL---------------PS-P-----------------TKNDRASIVGDAI--EY
210	TraesCS6A02G268800.1#domain1#PF00010	91.5%	26.0%	--KANFATEKERREQINVKYGALRSLL---------------PS-P-----------------TKNDRASIVGDAI--EY
211	TraesCS2D02G441800.1#domain1#PF00010	91.5%	28.6%	---ANFATERERREQLNVKYKTLRMLF---------------PN-P-----------------TKNDRASVVGDAI--EY
212	TraesCS2A02G442700.1#domain1#PF00010	91.5%	28.6%	---ANFATERERREQLNVKYKTLRMLF---------------PN-P-----------------TKNDRASVVGDAI--EY
213	TraesCS2B02G463800.1#domain1#PF00010	91.5%	28.6%	---ANFATERERREQLNVKYKTLRMLF---------------PN-P-----------------TKNDRASVVGDAI--EY
214	TraesCS6D02G185100.1#domain1#PF00010	91.5%	18.9%	RKVHKADCERMRRDKLNEQFMELGTTLD--------------PD-R-----------------PRHDKATILGDTV--QM
215	TraesCS6B02G220300.1#domain1#PF00010	91.5%	18.9%	RKVHKADRERMRRDKLNEQFMELGTTLD--------------PD-R-----------------PRHDKATILGDTV--QM
216	TraesCS6A02G204300.1#domain1#PF00010	91.5%	18.9%	RKVHKADRERMRRDKLNEQFLELGTTLD--------------PD-R-----------------PRHDKATILGDTV--QM
217	TraesCS1D02G280600.1#domain1#PF00010	93.6%	18.0%	---TKACREKLRRDRLNERFSELCAVLE--------------PGKP-----------------PKADKVAILSDAT--RL
218	TraesCS1B02G290500.1#domain1#PF00010	93.6%	18.0%	---TKACREKLRRDRLNERFSELCAVLE--------------PGKP-----------------PKADKVAILSDAT--RL
219	TraesCS1A02G281300.1#domain1#PF00010	93.6%	18.0%	---TKACREKLRRDRLNERFNELCAVLE--------------PGKP-----------------PKADKVAILSDAT--RL
220	TraesCS6A02G045100.1#domain1#PF00010	93.6%	14.1%	---SKACREKMRRDKLNDRFLELCSVMNSGKHSGLEECSASNPGKN-----------------AKLDKASILSDAT--RM
221	TraesCS6D02G051600.2#domain1#PF00010	93.6%	14.1%	---SKACREKMRRDKLNDRFLELCSVMNSGKHTGVEECSASNPGKN-----------------AKLDKASILSDAT--RM
222	TraesCS6B02G060700.2#domain1#PF00010	93.6%	14.1%	---SKACREKMRRDKLNDRFLELCSVMNSGKHTGLEECSASNPGKN-----------------AKLDKASILSDAT--RM
223	TraesCS7A02G307700.1#domain1#PF00010	93.6%	17.6%	--TSKACREKVRRDKLNDRFLELGTTLD--------------PGKP-----------------VKADKAAILSDAT--RM
224	TraesCS7B02G208000.1#domain1#PF00010	93.6%	17.6%	--TSKACREKVRRDKLNDRFLELGTTLD--------------PGKP-----------------VKADKAAILSDAT--RM
225	TraesCS7D02G304500.1#domain1#PF00010	93.6%	17.6%	--TSKACREKVRRDKLNDRFLELGTTLD--------------PGKP-----------------VKADKAAILSDAT--RM
226	TraesCS2B02G240600.1#domain1#PF00010	93.6%	16.0%	---SKACREKVRRDKLNERFLELSAVLD--------------PGKT-----------------PKIDKCAILNDAI--RA
227	TraesCS2A02G215600.1#domain1#PF00010	93.6%	16.0%	---SKACREKVRRDKLNERFLELGAVLD--------------PGKT-----------------PKIDKCAILNDAI--RA
228	TraesCS2D02G221200.1#domain1#PF00010	93.6%	16.0%	---SKACREKVRRDKLNERFLELGAVLD--------------PGKT-----------------PKIDKCAILNDAI--RA
229	TraesCS7D02G360600.1#domain1#PF00010	91.5%	26.5%	---HHVISERRRRERLNDSFQTLRALL---------------PPG------------------SKKDKANVLASTT--EY
230	TraesCS7B02G265900.1#domain1#PF00010	91.5%	26.5%	---HHVISERRRRERLNDSFQTLRALL---------------PPG------------------SKKDKANVLASTT--EY
231	TraesCS7A02G362500.1#domain1#PF00010	91.5%	26.5%	---HHVISERRRRERLNDSFQTLRALL---------------PPG------------------SKKDKANVLASTT--EY
232	TraesCS6A02G186200.1#domain1#PF00010	87.2%	21.3%	---HHMISERRRRERLNESFEALRGLL---------------PPG------------------SKKDKATVLANTL--DY
233	TraesCS6D02G173300.1#domain1#PF00010	87.2%	21.3%	---HHMISERRRRERLNESFEALRGLL---------------PPG------------------SKKDKATVLANTL--DY
234	TraesCS6B02G215200.1#domain1#PF00010	87.2%	21.3%	---HHMISERRRRERLNESFEALRGLL---------------PPG------------------SKKDKATVLANTL--DY
235	TraesCS5A02G251390.4#domain1#PF00010	95.7%	30.8%	-KSKHSATEQRRSKINDRFQILRDLL---------------PHTD-----------------QKRDKATFLLEVI--EY
236	TraesCS6A02G288100.3#domain1#PF00010	95.7%	36.5%	-RSKHSATEQRRTKINDRLDVLRDLL---------------PNCD-----------------QKRDKASFLLQVI--EY
237	TraesCS6B02G317600.2#domain1#PF00010	95.7%	36.5%	-RSKHSATEQRRTKINDRLDILRDLL---------------PNCD-----------------QKRDKASFLLEVI--EY
238	TraesCS6D02G270900.1#domain1#PF00010	95.7%	36.5%	-RSKHSATEQRRTKINDRLDILRDLL---------------PNCD-----------------QKRDKASFLLEVI--EY
239	TraesCS5A02G466300.1#domain1#PF00010	91.5%	26.0%	--RNHREAEKRRERIKSHLDRLRAVL---------------ACD------------------PKIDKATLLAKAV--ER
240	TraesCS5B02G478000.1#domain1#PF00010	91.5%	26.0%	--RNHREAEKRRERIKSHLDRLRAVL---------------ACD------------------PKIDKATLLAKAV--ER
241	TraesCS5D02G479100.1#domain1#PF00010	91.5%	26.0%	--RNHREAEKRRERIKSHLDRLRAVL---------------ACD------------------PKIDKATLLAKAV--ER
242	TraesCS1B02G129200.1#domain1#PF00010	91.5%	29.2%	----HSESERRRRERINTHLATLRRMI---------------PDA------------------NQMDKATLLACVV--NQ
243	TraesCS1D02G112000.1#domain1#PF00010	91.5%	29.2%	----HSESERRRRERINTHLATLRRMI---------------PDA------------------NQMDKATLLACVV--NQ
244	TraesCS1A02G110400.1#domain1#PF00010	91.5%	29.2%	----HSLSERRRRERINTHLATLRRMI---------------PDA------------------NQMDKATLLACVV--NQ
245	TraesCS3A02G102900.1#domain1#PF00010	91.5%	28.6%	---AHSEAERRRRVRINAHLTALRRMI---------------PDT------------------KQMDKAALLARVV--DQ
246	TraesCS3D02G105100.2#domain1#PF00010	91.5%	29.2%	----HSEAERRRRVRINAHLTALRRMI---------------PDT------------------KQMDKAALLGRVV--DQ
247	TraesCS3B02G120200.1#domain1#PF00010	93.6%	30.6%	----HSEAERRRRVRINAHLTALRRMI---------------PDT------------------KQMDKAALLARVV--DQ
248	TraesCS3B02G120500.1#domain1#PF00010	93.6%	30.6%	----HSEAERRRRVRINAHLTALRRMI---------------PDT------------------KQMDKAALLARVV--DQ
249	TraesCS3A02G102600.1#domain1#PF00010	91.5%	29.2%	----HSEAERRRRVRINAHLTALRRMI---------------PDT------------------KQMDKAALLARVV--DQ
250	TraesCS2B02G494900.1#domain1#PF00010	93.6%	35.3%	--RSHSEAERKRRQRINAHLATLRTLV---------------PSA------------------SRMDKAALLGEVV--RH
251	TraesCS2A02G472300.1#domain1#PF00010	93.6%	35.3%	--RSHSEAERKRRQRINAHLATLRTLV---------------PSA------------------SRMDKAALLGEVV--RH
252	TraesCS2D02G472000.1#domain1#PF00010	93.6%	35.3%	--RSHSEAERKRRQRINAHLATLRTLV---------------PSA------------------SRMDKAALLGEVV--RH
253	TraesCS5A02G245600.1#domain1#PF00010	91.5%	32.0%	--RSHSEAERKRRQRINTHLATLRSLL---------------PSA------------------SQMDKAALLGEVV--RH
254	TraesCS5D02G252300.1#domain1#PF00010	91.5%	32.0%	--RSHSEAERKRRQRINTHLATLRSLL---------------PSA------------------SQMDKAALLGEVV--RH
255	TraesCS5B02G243000.1#domain1#PF00010	91.5%	32.0%	--RSHSEAERKRRQRINTHLATLRSLL---------------PSA------------------SQMDKAALLGEVV--RH
256	TraesCS5B02G229000.1#domain1#PF00010	91.5%	38.0%	--RSHSEAERRRRQRINGHLARLRSLL---------------PNT------------------TKTDKASLLAEVL--EH
257	TraesCS5A02G230500.1#domain1#PF00010	91.5%	38.0%	--RSHSEAERRRRQRINGHLARLRSLL---------------PNT------------------TKTDKASLLAEVL--EH
258	TraesCS5D02G237300.1#domain1#PF00010	91.5%	38.0%	--RSHSEAERRRRQRINGHLARLRSLL---------------PNT------------------TKTDKASLLAEVL--EH
259	TraesCS3A02G142900.1#domain1#PF00010	91.5%	32.7%	---SHSEAERRRRERINAHLATLRSMV---------------PCT------------------DKMDKAALLAEVI--NH
260	TraesCS3D02G144700.1#domain1#PF00010	91.5%	30.6%	---SHSEAERRRRERINAHLATLRSML---------------PCT------------------DKMDKAALLAEVI--NH
261	TraesCS3A02G252900.1.cds1#domain2#PF00010	95.7%	24.5%	---NHVEAERQRREKLNQRFYALRAVV---------------PKIS------------------KMDKASLLSDAI--AY
262	TraesCS3B02G284800.1.cds1#domain2#PF00010	95.7%	24.5%	---NHVEAERQRREKLNQRFYALRAVV---------------PKIS------------------KMDKASLLSDAI--AY
263	TraesCS3D02G253700.1.cds1#domain2#PF00010	95.7%	24.5%	---NHVEAERQRREKLNQRFYALRAVV---------------PKIS------------------KMDKASLLSDAI--AY
264	TraesCS3B02G288700.1#domain2#PF00010	95.7%	24.5%	---NHVEAERQRREKLNQRFYALRAVV---------------PKIS------------------KMDKASLLSDAI--AY
265	5gnj:G	95.7%	17.3%	---NHVEAERQRREKLNQRFYALRAVV---------------PNVS------------------KMDKASLLGDAI--AY
266	TraesCS1B02G208000.1.cds1#domain2#PF00010	93.6%	27.1%	---NHVEAERQRREKLNQRFYALRAVV---------------PNVS------------------KMDKASLLGDAI--SY
267	TraesCS1D02G196900.1.cds1#domain2#PF00010	93.6%	27.1%	---NHVEAERQRREKLNQRFYALRAVV---------------PNVS------------------KMDKASLLGDAI--SY
268	TraesCS1A02G193200.1#domain2#PF00010	93.6%	27.1%	---NHVEAERQRREKLNQRFYALRAVV---------------PNVS------------------KMDKASLLGDAI--SY
269	TraesCS3B02G185400.1#domain2#PF00010	95.7%	28.6%	---NHVEAERQRREKLNQRFYALRAVV---------------PNIS------------------KMDKASLLGDAI--AY
270	TraesCS3D02G166300.2#domain2#PF00010	95.7%	28.6%	---NHVEAERQRREKLNQRFYALRAVV---------------PNIS------------------KMDKASLLGDAI--AY
271	TraesCS3A02G158600.1.cds1#domain2#PF00010	95.7%	28.6%	---NHVEAERQRREKLNQRFYALRAVV---------------PNIS------------------KMDKASLLGDAI--AY
272	TraesCS4A02G028900.1.cds1#domain2#PF00010	95.7%	30.6%	---NHVEAERQRREKLNQRFYALRAVV---------------PNIS------------------KMDKASLLGDAI--TH
273	TraesCS4D02G275500.1.cds1#domain2#PF00010	95.7%	30.6%	---NHVEAERQRREKLNQRFYALRAVV---------------PNIS------------------KMDKASLLGDAI--TH
274	TraesCS4B02G276900.1.cds1#domain2#PF00010	95.7%	30.6%	---NHVEAERQRREKLNQRFYALRAVV---------------PNIS------------------KMDKASLLGDAI--TH
275	TraesCS2A02G448500.1.cds1#domain1#PF00010	93.6%	28.6%	--VSHVQAERLRRDKLNRRFCDLRAAV---------------PNVS------------------RMNKASLLADAV--AY
276	TraesCS2B02G469200.1#domain1#PF00010	93.6%	28.6%	--VSHVQAERLRRDKLNRRFCDLRAAV---------------PNVS------------------RMDKASLLADAV--AY
277	TraesCSU02G222600.1#domain1#PF00010	93.6%	28.6%	--VSHVQAERLRRDKLNRRFCDLRAAV---------------PNVS------------------RMDKASLLADAV--AY
278	TraesCS2B02G469300.1.cds1#domain1#PF00010	93.6%	28.6%	--VSHVQAERLRRDKLNRRFCDLRAAV---------------PNVS------------------RMDKASLLADAV--AY
279	TraesCS5A02G306600.1.cds1#domain1#PF00010	93.6%	27.1%	---SHVEAERQRRDKLNRLFCDLRAAV---------------PRVS------------------RMDKASLLADAV--AY
280	TraesCS5D02G313700.1.cds1#domain1#PF00010	93.6%	27.1%	---SHVEAERQRRDKLNRRFCDLRAAV---------------PKVS------------------RMDKASLLADAV--AY
281	TraesCS5B02G307100.1.cds1#domain1#PF00010	93.6%	29.2%	---SHVEAERQRRDKLNRRFCDLRAAV---------------PNVS------------------RMDKASLLADAV--AY
282	TraesCS5A02G306700.1.cds1#domain1#PF00010	93.6%	26.5%	--VSHVEAERQRRDKLNRRFCDLRAAV---------------PRVS------------------RMDKASLLADAV--AY
283	TraesCS7D02G102200.1.cds1#domain1#PF00010	93.6%	24.5%	--VSHVQAERQRRNKLNRRFCDLRAAV---------------PTVS------------------RMDKASLLSDAT--IY
284	TraesCS1A02G369200.1.cds1#domain1#PF00010	93.6%	27.7%	----HVQAERQRRDKLNRRFCDLRAVV---------------PNVS------------------RMDKASLLADAA--TY
285	TraesCS1D02G374800.1.cds1#domain1#PF00010	93.6%	27.7%	----HVQAERQRRDKLNRRFCDLRAVV---------------PNVS------------------RMDKASLLADAA--TY
286	TraesCS5A02G306400.1.cds1#domain1#PF00010	93.6%	24.5%	--VSHVEAERQRRDKLNRRFCDLRAAV---------------PTVS------------------RMDKASLLADAA--AY
287	TraesCS5B02G307000.1.cds1#domain1#PF00010	93.6%	24.5%	--VSHVEAERQRRDKLNRRFCDLRAAV---------------PTVS------------------RMDKASLLADAA--AY
288	TraesCS5D02G313600.1.cds1#domain1#PF00010	93.6%	24.5%	--VSHVEAERQRRDKLNRRFCDLRAAV---------------PTVS------------------RMDKASLLADAA--AY
289	TraesCS5A02G306200.1.cds1#domain1#PF00010	93.6%	24.5%	--VSHVEAERQRRDKLNRRFCDLRAAV---------------PTVS------------------RMDKASLLADAA--AY

```
290 TraesCS5D02G313500.1.cds1#domain1#PF00010  93.6%  24.5%  --VSHVEAERQRRDKLNRRFCDLRAAV---------------PTVS------------------RMDKASLLADAA--AY
291 TraesCS5B02G306800.1.cds1#domain1#PF00010  93.6%  24.5%  --VSHVEAERQRRDKLNRRFCDLRAAV---------------PTVS------------------RMDKASLLADAA--AY
292 TraesCS5A02G306500.1.cds1#domain1#PF00010  93.6%  22.4%  --VSHVEAEQQRRDKLNRRFCDLRAAV---------------PTVS------------------RMDKASLLADAA--AY
293 TraesCS5B02G306600.1#domain1#PF00010       93.6%  25.0%  ---SHVEAERQRREKLNRRFCDLRAAV---------------PTVS------------------RMDRASLLADAA--DY
294 TraesCS5D02G313400.1.cds1#domain1#PF00010  93.6%  25.0%  ---SHVEAERQRREKLNRRFCDLRAAV---------------PTVS------------------RMDRTSLLADAA--AY
295 TraesCS3D02G106600.2#domain1#PF00010       93.6%  25.0%  ---QHMISERKRREKLNDSFQALKTVL---------------PPGS------------------KKDKTSILITAR--EY
296 TraesCS3A02G104400.1#domain1#PF00010       93.6%  25.0%  ---QHMISERKRREKLNDSFQALKTVL---------------PPGS------------------KKDKTSILITAR--EY
297 TraesCS3B02G122800.1#domain1#PF00010       93.6%  25.0%  ---QHMISERKRREKLNDSFQALKTVL---------------PPGS------------------KKDKTSILITAR--EY
298 TraesCS3B02G122900.1#domain1#PF00010       95.7%  26.5%  ---QHMISERKRREKLNDSFQALKTVL---------------PRGS------------------KKDKTSILITAR--EY
299 TraesCS3B02G236900.1#domain1#PF00010       95.7%  25.0%  ----HMMSERKRREKLNDSFHTLRSLL---------------PPCS------------------KKDKTTVLTKAA--GY
300 TraesCS3A02G204800.1#domain1#PF00010       95.7%  25.0%  ----HMMSERKRREKLNDSFHTLRSLL---------------PPCS------------------KKDKTTVLTKAA--GY
301 TraesCS3D02G210600.1#domain1#PF00010       95.7%  25.0%  ----HMMSERKRREKLNDSFHTLRSLL---------------PPCS------------------KKDKTTVLTKAA--WY
302 TraesCS4A02G087000.1#domain1#PF00010       93.6%  36.7%  --KSHSEAERRRRERINSHLARLRSLL---------------PNTT------------------KTDKASLLAEVI--QH
303 TraesCS4B02G217400.1#domain1#PF00010       93.6%  36.7%  --KSHSEAERRRRERINSHLARLRSLL---------------PNTT------------------KTDKASLLAEVI--QH
304 TraesCS4D02G217700.1#domain1#PF00010       93.6%  36.7%  --KSHSEAERRRRERINSHLARLRSLL---------------PNTT------------------KTDKASLLAEVI--QH
305 TraesCS2A02G281200.1#domain1#PF00010       93.6%  36.2%  -----IVSERKRRFRMKEKLYELRALV---------------PNIT------------------KMDKASIIADAV--AY
306 TraesCS2D02G280100.1#domain1#PF00010       93.6%  36.2%  -----IVSERKRRFRMKEKLYELRALV---------------PNIT------------------KMDKASIIADAV--AY
307 TraesCS2B02G298600.1#domain1#PF00010       93.6%  36.2%  -----IVSERKRRFRMKEKLYELRALV---------------PNIT------------------KMDKASIIADAV--AY
308 TraesCS3A02G442600.1#domain1#PF00010       85.1%  34.9%  ---------RLRGRTLNGKLYALRSVV---------------PNIT------------------KMDKVSILRDAV--EY
309 TraesCS3D02G435200.1#domain1#PF00010       85.1%  32.6%  ---------RLRGRTLNGKLYTLRSVV---------------PNIT------------------KMDKVSILRDAI--EY
310 TraesCS2B02G289900.1#domain1#PF00010       95.7%  31.2%  ----NILMERDRRRKLNEKLYTLRSVV---------------PNIT------------------KMDKASIIKDAI--EY
311 TraesCS2D02G270300.1#domain1#PF00010       95.7%  31.2%  ----NILMERDRRRKLNEKLYTLRSVV---------------PNIT------------------KMDKASIIKDAI--EY
312 TraesCS2A02G271700.1#domain1#PF00010       95.7%  29.2%  ----NILMERDRRRKLNEKLYTLRSVV---------------PNIT------------------KMDKASIIKDAI--EY
313 TraesCS4B02G364800.1#domain1#PF00010       95.7%  30.6%  ---KNIINERHRRRMLNEKLYALRRVV---------------PNIT------------------KMDKASIIQDAI--AY
314 TraesCS5A02G533200.1#domain1#PF00010       95.7%  30.6%  ---KNIINERHRRRMLNEKLYALRRVV---------------PNIT------------------KMDKASIIQDAI--AY
315 TraesCSU02G138900.1#domain1#PF00010        95.7%  30.6%  ---KNIINERHRRRMLNEKLYALRRVV---------------PNIT------------------KMDKASIIQDAI--AY
316 TraesCS2D02G270000.2#domain1#PF00010       95.7%  32.7%  ---KNIIMERDRRRRLNEKLYNLRGVV---------------PNIT------------------KMDKASIIQDAI--AY
317 TraesCS2B02G289700.2#domain1#PF00010       95.7%  32.7%  ---KNIIMERDRRRRLNEKLYNLRGVV---------------PNIT------------------KMDKASIIQDAI--AY
318 TraesCS5A02G533900.1#domain1#PF00010       91.5%  30.4%  ------LNERDRRRRLNEKLYAIRGVV---------------PNIT------------------KMNKASIIQDAI--AY
319 TraesCSU02G138400.2#domain1#PF00010        91.5%  30.4%  ------LNERDRRMRLNEKLYAIRGVV---------------PNIT------------------KMDKASIIQDAI--AY
320 TraesCS3B02G018400.1#domain1#PF00010       95.7%  28.8%  RASKNIVEERDRRRRLNEKLYAIRGVV---------------PNIT------------------KMDKASIIQDAV--AY
321 TraesCS3A02G028200.3#domain1#PF00010       95.7%  28.8%  RASKNIVEERDRRRRLNEKLYAIRGVV---------------PNIT------------------KMDKASIIQDAV--AY
322 TraesCS3D02G017700.1#domain1#PF00010       95.7%  28.8%  RASKNIVEERVRRRRLNEKLYAIRGVV---------------PNIT------------------KMDKASIIQDAV--AY
323 TraesCS2B02G237300.1#domain1#PF00010       95.7%  40.8%  ---KNLDAERRRRGRLNDNILKLRAVV---------------PNIT------------------KMSKESTLADAI--GH
324 TraesCS2A02G212200.1#domain1#PF00010       95.7%  38.8%  ---KNLDAERRRRGRLNTNILKLRAVV---------------PNIT------------------KMSKESTLADAI--GH
325 TraesCS2D02G218100.1#domain1#PF00010       95.7%  38.8%  ---KNLDAERRRRGRLNTNILKLRAVV---------------PNIT------------------KMSKESTLADAI--GH
326 TraesCS4D02G224600.1#domain2#PF00010       95.7%   4.9%  --NTHVMTERRRREKLNEMFLVLKSLV---------------PSVH------------------KVDKASILTETI--GY
327 TraesCS2D02G575600.3#domain2#PF00010       95.7%  20.4%  ---THVLSERRHREKLNEMFLVLKSLV---------------PSIN------------------KMDKASILAETI--TY
328 TraesCSU02G029400.1#domain2#PF00010        95.7%  22.4%  ---THVLSERRRREKLNEMFLVLKSLV---------------PSIN------------------KMDKASILAETI--TY
329 TraesCS4B02G397400.1#domain2#PF00010       95.7%  22.4%  ---THVLSERRRREKLNEMFLVLKSLV---------------PSIN------------------KMDKASILAETI--TY
330 TraesCS5A02G558500.1#domain2#PF00010       95.7%  20.4%  ---THVLSERRRREKLNDMFLVLKSMV---------------PSIN------------------KMDKASILAETI--TY
331 TraesCS5A02G489500.1#domain2#PF00010       95.7%  20.4%  ---THVLSERRRREKLNDMFLVLKSMV---------------PSIN------------------KMDKASILAETI--TY
332 TraesCS4B02G397300.1#domain2#PF00010       95.7%  18.4%  ---THVLSERRRREKLNDMFLVLKSMI---------------PSIN------------------KMDKASILAETI--TY
333 TraesCS2D02G406900.1#domain2#PF00010       95.7%  22.0%  --KNHVISERRRREKLNEMFLILKSLV---------------PSIH------------------KVDKASILAETI--AY
334 TraesCS2B02G428000.1#domain2#PF00010       95.7%  22.0%  --KNHVISERRRREKLNEMFLILKSLV---------------PSIH------------------KVDKASILAETI--AY
335 TraesCS2A02G409600.2#domain2#PF00010       95.7%  22.0%  --KNHVISERRRREKLNEMFLILKSLV---------------PSIH------------------KVDKASILAETI--AY
336 TraesCSU02G228400.1#domain1#PF00010        95.7%  22.0%  --KNHVISERRRREKLNEMFLILKSLV---------------PSIH------------------KVDKASILAETI--AY
337 TraesCSU02G237100.1#domain1#PF00010        95.7%  22.0%  --KNHVISERRRREKLNEMFLILKSLV---------------PSIH------------------KVDKASILAETI--AY
338 TraesCS2A02G409400.1#domain2#PF00010       95.7%  22.0%  --KNHVISERRRREKLNEMFLILKSLV---------------PSIH------------------KVDKASILAETI--TY
339 TraesCSU02G116000.1#domain2#PF00010        95.7%  26.5%  ---AHVISERRRRERLNEMFLVLKSLV---------------PSIH------------------KVDKASILVETI--AY
340 TraesCS4B02G397800.1#domain2#PF00010       95.7%  26.5%  ---NHVISERRRRERLNEMFMVLKSLV---------------PSIH------------------KVDKASILLQTI--AY
341 TraesCS1B02G112900.1#domain2#PF00010       97.9%  20.0%  --ASHVMQERKRREKLNERFIILRSLV---------------PFVTKARTQSSAKKLQSFLHGCQMDKASILGDTI--EY
342 TraesCS1B02G113100.4#domain2#PF00010       95.7%  27.1%  --ASHVMQERKRREKLNERFIILRSLV---------------PFVTK------------------MDKASILGDTI--EY
343 TraesCS1A02G102400.2#domain2#PF00010       95.7%  27.1%  --ASHVMQERKRREKLNERFIVLRSLV---------------PFVTK------------------MDKASILGDTI--EY
344 TraesCS6B02G095600.1#domain1#PF00010       91.5%  39.1%  ------EAERKRRKKLNERLYKLRSLV---------------PNIT------------------KMDRASILGDAI--DY
345 TraesCS6D02G069300.1#domain1#PF00010       91.5%  41.3%  ------EAERKRRKKLNDRLYKLRSLV---------------PNIT------------------KMDRASILGDAI--DY
346 TraesCS6A02G071200.1#domain1#PF00010       91.5%  41.3%  ------EAERKRRRKLNDRLYKLRSLV---------------PNIT------------------KMDRASILGDAI--DY
347 TraesCS6B02G095700.1#domain1#PF00010       91.5%  39.1%  ------IAERRRRKKLNNRLYTLRSLV---------------PNIT------------------KMDRASILGDAI--DY
348 TraesCS6D02G069400.1#domain1#PF00010       91.5%  39.1%  ------IAERKRRKKLNNRLYTLRSLV---------------PNIT------------------KMDRASILGDAI--DY
349 TraesCS6A02G071300.1#domain1#PF00010       91.5%  39.1%  ------IAERKRRKKLNNRLYTLRSLV---------------PNIT------------------KMDRASILGDAI--DY
350 TraesCS4B02G051500.2#domain1#PF00010       89.4%  33.3%  ------MAERRRRKKLNDRLYALRSVV---------------PRIS------------------KMDRASILGDAI--EY
351 TraesCS4D02G051600.2#domain1#PF00010       89.4%  33.3%  ------MAERRRRKKLNDRLYALRSVV---------------PRIS------------------KMDRASILGDAI--EY
352 TraesCS4A02G263300.2#domain1#PF00010       89.4%  33.3%  ------LAERRRRKKLNDRLYALRSVV---------------PRIS------------------KMDRASILGDAI--EY
353 TraesCS3B02G476000.1#domain1#PF00010       89.4%  31.1%  ------MAERRRRKKLNDRLYMLRSVV---------------PKIS------------------KMDRASILGDAI--DY
354 TraesCS3D02G434900.1#domain1#PF00010       89.4%  31.1%  ------MAERRRRKKLNDRLYMLRSVV---------------PKIS------------------KMDRASILGDAI--DY
355 TraesCS3A02G442200.1#domain1#PF00010       89.4%  31.1%  ------MAERRRRKKLNDRLYMLRSVV---------------PKIS------------------KMDRASILGDAI--DY
356 TraesCS7A02G311800.1#domain1#PF00010       89.4%  33.3%  ------MAERRRRKKLNDRLYMLRSVV---------------PKIS------------------KMDRASILGDAI--EY
357 TraesCS7B02G211600.1#domain1#PF00010       89.4%  33.3%  ------MAERRRRKKLNDRLYMLRSVV---------------PKIS------------------KMDRASILGDAI--EY
358 TraesCS7D02G308300.1#domain1#PF00010       89.4%  33.3%  ------MAERRRRKKLNDRLYMLRSVV---------------PKIS------------------KMDRASILGDAI--EY
359 TraesCS3A02G057900.1#domain1#PF00010       89.4%  31.1%  ------MAERRRRKRLNDRLSMLRSTV---------------PKIS------------------KMDRTATLGDTI--DY
360 TraesCS3D02G058100.1#domain1#PF00010       89.4%  28.9%  ------MAERRRQKRLNDRLSMLRSTV---------------PKIS------------------KMDRTATLGDTI--DY
361 TraesCS7A02G543300.1#domain1#PF00010       89.4%  31.1%  ------MAERRRRKRLNDRLSMLRSVV---------------PKIS------------------KMDRTSILGDTI--DY
362 TraesCS7D02G529200.1#domain1#PF00010       89.4%  31.1%  ------MAERRRRKRLNDRLSMLRSVV---------------PKIS------------------KMDRTSILGDTI--DY
363 TraesCS7B02G465400.1#domain1#PF00010       89.4%  31.1%  ------MAERRRRKRLNDRLSMLRSVV---------------PKIS------------------KMDRTSILGDTI--DY
364 TraesCSU02G069400.1#domain1#PF00010        91.5%  28.3%  -----LMAERRRRKRLNDRLSMLRSIV---------------PKIS------------------KMDRTSILEDTL--DY
365 TraesCSU02G069300.1#domain1#PF00010        91.5%  28.3%  -----LMAERRRRKRLNDRLSMLRSIV---------------PKIS------------------KMDRTSILEDTL--DY
366 TraesCS4D02G328800.1#domain1#PF00010       89.4%  26.7%  ------MAERRRRKRLNDRLSMLRSIM---------------PKIT------------------KMDRTSILGDTI--DY
367 TraesCS4B02G331900.1#domain1#PF00010       89.4%  28.9%  ------MAERRRRKRLNDRLSMLRSIV---------------PKIS------------------KMDRTSILGDTI--DY
368 TraesCS4B02G332000.1#domain1#PF00010       93.6%  26.0%  -RSKNLMAERRRRKRLNDRLSMLRSIV---------------PKIS------------------KMDRTSILGDTI--DY
369 TraesCS5A02G503400.1#domain1#PF00010       89.4%  28.9%  ------MAERRRRKRLNDRLSMLRSIV---------------PKVT------------------KMDRTSILGDTI--DY
370 TraesCS4D02G328900.1#domain1#PF00010       89.4%  28.9%  ------MAERRRRKRLNDRLSMLRSIV---------------PKIT------------------KMDRTSILGDTI--DY
371 TraesCS4D02G329100.1#domain1#PF00010       89.4%  28.9%  ------MAERRRRKRLNDRLSMLRSIV---------------PKIT------------------KMDRTSILGDTI--DY
372 TraesCS5A02G503300.1#domain1#PF00010       89.4%  28.9%  ------MAERRRRKRLNDRLSMLRSIV---------------PKIT------------------KMDRTSILGDTI--DY
373 TraesCS5A02G503500.1#domain1#PF00010       89.4%  28.9%  ------MAERRRRKRLNDRLSMLRSIV---------------PKIT------------------KMDRTSILGDTI--DY
374 TraesCS4D02G329000.2#domain1#PF00010       89.4%  28.9%  ------MAERRRRKRLNDRLSMLRSIV---------------PKIT------------------KMDRTSILGDTI--DY
    consensus/100%                                          .........p.pt..ht..h..ht..h...............s.......................t....l..hh....
    consensus/90%                                           ........t+.RRpphsphh..Lpthl...............Pt.....................hshsshlttsh..ta
    consensus/80%                                           ........ERpRRc+lNpphh.Lptll...............Psh....................hDpsohlspsl..pa
    consensus/70%                                           ....+..sERpRRc+lNc+hhtLpsll...............Pshp..................phD+uohLscsl..pY

                                                 cov    pid  81        .         1         .         .         .         .         :         . 160
  1 TraesCS6B02G244800.1#domain1#PF00010      100.0% 100.0%  IKGLQ---------------------------------------------------------------------------
  2 TraesCS6A02G214900.1#domain1#PF00010      100.0% 100.0%  IKGLQ---------------------------------------------------------------------------
  3 TraesCS6D02G197500.1#domain1#PF00010      100.0%  97.9%  IKGLQ---------------------------------------------------------------------------
  4 TraesCS3D02G388700.1#domain1#PF00010      100.0%  83.0%  IKVLQ---------------------------------------------------------------------------
  5 TraesCS3B02G426900.1#domain1#PF00010      100.0%  83.0%  IKVLQ---------------------------------------------------------------------------
  6 TraesCS3A02G395000.1#domain1#PF00010      100.0%  83.0%  IKVLQ---------------------------------------------------------------------------
  7 TraesCS7A02G245800.1#domain1#PF00010      100.0%  78.7%  IKELQ---------------------------------------------------------------------------
  8 TraesCS7D02G244300.1#domain1#PF00010      100.0%  78.7%  IKELQ---------------------------------------------------------------------------
```

9	TraesCS7B02G145800.1#domain1#PF00010	100.0%	78.7%	IKELQ---
10	TraesCS5A02G264800.1#domain1#PF00010	100.0%	76.6%	IKELQ---
11	TraesCS5D02G272800.1#domain1#PF00010	100.0%	76.6%	IKELQ---
12	TraesCS5B02G264300.1#domain1#PF00010	100.0%	76.6%	IKELQ---
13	TraesCS4B02G317900.1#domain1#PF00010	97.9%	37.5%	MKSLQ---
14	TraesCS3B02G010400.1#domain1#PF00010	93.6%	44.4%	VKFLQ---
15	TraesCS3B02G007700.1#domain1#PF00010	93.6%	44.4%	VKFLQ---
16	TraesCS3D02G004300.1#domain1#PF00010	93.6%	44.4%	VKFLQ---
17	TraesCS3A02G006300.1#domain1#PF00010	87.2%	47.6%	VKFLQ---
18	TraesCS6A02G276200.1#domain1#PF00010	87.2%	45.2%	VKFLQ---
19	TraesCS6B02G303600.1#domain1#PF00010	89.4%	46.5%	VKFLQ---
20	TraesCS6D02G256600.1#domain1#PF00010	89.4%	46.5%	VKFLQ---
21	TraesCS6D02G256400.1#domain1#PF00010	89.4%	44.2%	VKFLQ---
22	TraesCS6D02G256500.1#domain1#PF00010	87.2%	45.2%	VKFLQ---
23	TraesCS6A02G276100.1#domain1#PF00010	87.2%	45.2%	VKFLQ---
24	TraesCS6B02G303500.1#domain1#PF00010	87.2%	45.2%	VKFLQ---
25	TraesCS6D02G256300.1#domain1#PF00010	87.2%	45.2%	VKFLQ---
26	TraesCS4B02G257200.1#domain1#PF00010	87.2%	45.2%	IKFMQ---
27	TraesCS4A02G047700.1#domain1#PF00010	87.2%	45.2%	IKFMQ---
28	TraesCS4D02G257100.1#domain1#PF00010	87.2%	45.2%	IKFMQ---
29	TraesCS5A02G067600.1#domain1#PF00010	87.2%	50.0%	VKFLQ---
30	TraesCS5D02G078800.1#domain1#PF00010	87.2%	50.0%	VKFLQ---
31	TraesCS5B02G074500.1#domain1#PF00010	87.2%	52.4%	VKFLQ---
32	TraesCS2A02G194200.1#domain1#PF00010	87.2%	50.0%	VKFMQ---
33	TraesCS2B02G212700.1#domain1#PF00010	87.2%	50.0%	VKFMQ---
34	TraesCS2D02G193700.1#domain1#PF00010	87.2%	50.0%	VKFMQ---
35	TraesCS4A02G234500.1#domain1#PF00010	87.2%	50.0%	VKFLQ---
36	TraesCS4D02G079500.1#domain1#PF00010	87.2%	50.0%	VKFLQ---
37	TraesCS4B02G080700.1#domain1#PF00010	87.2%	50.0%	VKFLQ---
38	TraesCS5B02G406100.1#domain1#PF00010	87.2%	52.4%	VKFLQ---
39	TraesCS5D02G411600.1#domain1#PF00010	87.2%	50.0%	VKFLQ---
40	TraesCS5A02G401300.1#domain1#PF00010	87.2%	50.0%	VKFLQ---
41	TraesCS1B02G359000.1#domain1#PF00010	85.1%	43.9%	VKFL--
42	TraesCS1A02G345200.1#domain1#PF00010	85.1%	43.9%	VKFL--
43	TraesCS1D02G347900.1#domain1#PF00010	85.1%	43.9%	VKFL--
44	TraesCS3A02G440600.1#domain1#PF00010	89.4%	48.8%	VKFLQ---
45	TraesCS3D02G433200.1#domain1#PF00010	89.4%	48.8%	VKFLQ---
46	TraesCS3B02G474700.1#domain1#PF00010	87.2%	50.0%	VKFLQ---
47	TraesCS3A02G350600.1.cds1#domain1#PF00010	85.1%	48.8%	VKFL--
48	TraesCS3B02G383000.1.cds1#domain1#PF00010	85.1%	48.8%	VKFL--
49	TraesCS3D02G344600.1.cds1#domain1#PF00010	85.1%	48.8%	VKFL--
50	TraesCS7B02G160500.1.cds1#domain1#PF00010	87.2%	21.7%	VKFLRLRRRKQSNRESARRSRSRKAAHLNELE----------AQVSQLRVEN---SSL---LRRLADVNQK---------
51	TraesCS7A02G340300.1#domain1#PF00010	100.0%	32.0%	VKFLQ---
52	TraesCS7B02G251900.1#domain1#PF00010	100.0%	32.0%	VKFLQ---
53	TraesCS7D02G347900.1#domain1#PF00010	100.0%	32.0%	VKFLQ---
54	TraesCS1B02G226200.2#domain1#PF00010	97.9%	46.8%	LKQLQ---
55	TraesCS1D02G215600.2#domain1#PF00010	97.9%	46.8%	LKQLQ---
56	TraesCS1A02G212700.1#domain1#PF00010	97.9%	46.8%	LKELQ---
57	TraesCS2D02G461900.1#domain1#PF00010	97.9%	42.6%	LKSLQ---
58	TraesCS2D02G461700.3#domain1#PF00010	97.9%	42.9%	LKSLQ---
59	TraesCS2A02G461700.2#domain1#PF00010	97.9%	42.9%	LKSLQ---
60	TraesCS2B02G483300.1#domain1#PF00010	97.9%	42.9%	LKSLQ---
61	TraesCS1B02G100400.2#domain1#PF00010	97.9%	43.8%	LKTLQ---
62	TraesCS1A02G083000.1#domain1#PF00010	97.9%	43.8%	LKTLQ---
63	TraesCS1D02G084200.1#domain1#PF00010	97.9%	43.8%	LKTLQ---
64	TraesCS5B02G054800.2#domain1#PF00010	97.9%	46.9%	LKSLQ---
65	TraesCS5A02G049600.1#domain1#PF00010	97.9%	46.9%	LKSLQ---
66	TraesCS5D02G060300.1#domain1#PF00010	97.9%	46.9%	LKSLQ---
67	TraesCS5A02G376500.1#domain1#PF00010	97.9%	45.8%	LKTLQ---
68	TraesCS5B02G380200.1#domain1#PF00010	97.9%	45.8%	LKTLQ---
69	TraesCS5D02G386500.1#domain1#PF00010	97.9%	45.8%	LKTLQ---
70	TraesCS2A02G253900.2#domain1#PF00010	97.9%	41.7%	LKSLQ---
71	TraesCS2B02G273500.1#domain1#PF00010	97.9%	41.7%	LKSLQ---
72	TraesCS2D02G254400.3#domain1#PF00010	97.9%	41.7%	LKSLQ---
73	TraesCS5A02G420200.1#domain1#PF00010	97.9%	41.7%	LKSLQ---
74	TraesCS5B02G422000.1#domain1#PF00010	97.9%	41.7%	LKSLQ---
75	TraesCS5D02G428400.2#domain1#PF00010	97.9%	41.7%	LKSLQ---
76	TraesCS6B02G411300.1#domain1#PF00010	97.9%	41.7%	LKHLQ---
77	TraesCS6A02G373500.2#domain1#PF00010	97.9%	43.8%	LKHLQ---
78	TraesCS6D02G357700.1#domain1#PF00010	97.9%	43.8%	LKHLQ---
79	TraesCS7A02G126900.1#domain1#PF00010	97.9%	43.8%	LKQLQ---
80	TraesCS7D02G124700.1#domain1#PF00010	97.9%	43.8%	LKQLQ---
81	TraesCS7B02G026300.1#domain1#PF00010	97.9%	43.8%	LKQLQ---
82	TraesCS6A02G190600.1#domain1#PF00010	95.7%	42.9%	LKQL--
83	TraesCS7A02G323000.1.cds1#domain1#PF00010	85.1%	51.2%	IKFL--
84	TraesCS7D02G319600.1.cds1#domain1#PF00010	85.1%	51.2%	IKFL--
85	TraesCS7B02G223900.1.cds1#domain1#PF00010	85.1%	51.2%	IKFL--
86	TraesCS7D02G263400.1.cds1#domain1#PF00010	85.1%	48.8%	VKFL--
87	TraesCS7A02G262600.1.cds1#domain1#PF00010	85.1%	48.8%	VKFL--
88	TraesCS7B02G303300.1#domain1#PF00010	100.0%	35.4%	VQSLQ---
89	TraesCS7D02G397300.1#domain1#PF00010	100.0%	35.4%	VQSLQ---
90	TraesCS7A02G157700.1#domain1#PF00010	97.9%	51.1%	VKFLQ---
91	TraesCS7D02G158300.1#domain1#PF00010	97.9%	51.1%	VKFLQ---
92	TraesCS7B02G062100.2#domain1#PF00010	97.9%	51.1%	VKFLQ---
93	TraesCS7A02G147500.1#domain1#PF00010	97.9%	48.9%	VKFLQ---
94	TraesCS7D02G149400.1#domain1#PF00010	97.9%	48.9%	VKFLQ---
95	TraesCS6B02G391100.1#domain1#PF00010	97.9%	46.8%	VKFLQ---
96	TraesCS6D02G341200.1#domain1#PF00010	97.9%	46.8%	VKFLQ---
97	TraesCS6A02G358400.3#domain1#PF00010	97.9%	48.9%	VKFLQ---
98	TraesCS5A02G225700.1#domain1#PF00010	97.9%	48.9%	VKFLQ---
99	TraesCS5D02G233000.1#domain1#PF00010	100.0%	46.9%	VKFLQ---
100	TraesCS5B02G224200.1#domain1#PF00010	100.0%	46.9%	VKFLQ---
101	TraesCS5D02G300100.1#domain1#PF00010	97.9%	40.8%	VQSLQ---
102	TraesCS5B02G292100.1#domain1#PF00010	97.9%	40.8%	VQSLQ---
103	TraesCS5D02G459800.1#domain1#PF00010	97.9%	32.7%	VQALE---
104	TraesCS5B02G457600.1#domain1#PF00010	97.9%	32.7%	VQALE---
105	TraesCS4D02G237100.3#domain1#PF00010	100.0%	40.0%	VQSLQ---
106	TraesCS4B02G235700.1#domain1#PF00010	97.9%	38.0%	VQSLQ---
107	TraesCS4A02G060200.2#domain1#PF00010	97.9%	38.0%	VQSLQ---

108	TraesCS4A02G255800.3#domain1#PF00010	97.9%	42.0%	VQSLQ--
109	TraesCS4D02G058900.1#domain1#PF00010	97.9%	40.0%	VQSLQ--
110	TraesCS4B02G059000.1#domain1#PF00010	97.9%	40.0%	VQSLQ--
111	TraesCS7D02G327900.1#domain1#PF00010	95.7%	24.5%	IKELQ--
112	TraesCS7A02G350900.1#domain1#PF00010	95.7%	24.5%	IKELQ--
113	TraesCS7B02G232000.1#domain1#PF00010	95.7%	24.5%	IKELQ--
114	TraesCS4B02G347100.1#domain1#PF00010	95.7%	22.2%	VKQLE--
115	TraesCS5A02G515800.1#domain1#PF00010	95.7%	22.2%	VKQLE--
116	TraesCS4D02G342000.1#domain1#PF00010	95.7%	22.2%	VKQLE--
117	TraesCS2B02G141600.1#domain1#PF00010	93.6%	24.5%	IREL---
118	TraesCS2D02G122800.1#domain1#PF00010	93.6%	26.0%	IREL---
119	TraesCS2A02G120700.1#domain1#PF00010	93.6%	26.0%	IREL---
120	TraesCS6B02G346100.1#domain1#PF00010	95.7%	20.4%	VKELE--
121	TraesCS6D02G294900.1#domain1#PF00010	95.7%	20.4%	VKELE--
122	TraesCS6A02G315700.1#domain1#PF00010	95.7%	20.4%	VKELE--
123	TraesCS1B02G300100.1#domain1#PF00010	95.7%	25.9%	IRELE--
124	TraesCS1D02G289400.1#domain1#PF00010	95.7%	25.9%	IRELE--
125	TraesCS1A02G290800.1#domain1#PF00010	95.7%	25.9%	IRELE--
126	TraesCS6D02G315200.1#domain1#PF00010	95.7%	20.4%	VKELE--
127	TraesCS6A02G335500.1#domain1#PF00010	95.7%	20.4%	VKELE--
128	TraesCS6B02G366200.1#domain1#PF00010	95.7%	20.4%	VKELE--
129	TraesCS6A02G264200.1#domain1#PF00010	95.7%	20.4%	VKELE--
130	TraesCS6B02G291200.1#domain1#PF00010	95.7%	20.4%	VKELE--
131	TraesCS6D02G250700.1#domain1#PF00010	95.7%	20.4%	VKELE--
132	TraesCS2B02G469700.1#domain1#PF00010	95.7%	20.4%	VKELE--
133	TraesCS2B02G469800.1#domain1#PF00010	95.7%	20.4%	VKELE--
134	TraesCS2D02G448000.1#domain1#PF00010	95.7%	20.4%	VKELE--
135	TraesCS2A02G448800.1#domain1#PF00010	95.7%	20.4%	VKELE--
136	TraesCS4B02G304500.1#domain1#PF00010	95.7%	20.4%	VKELE--
137	TraesCS4A02G404700.1#domain1#PF00010	95.7%	20.4%	VKELE--
138	TraesCS4D02G302700.1#domain1#PF00010	95.7%	20.4%	VKELE--
139	TraesCS5B02G245900.1#domain1#PF00010	95.7%	20.4%	VKELE--
140	TraesCS5A02G248200.1#domain1#PF00010	95.7%	20.4%	VKELE--
141	TraesCS5D02G255100.1#domain1#PF00010	95.7%	20.4%	VKELE--
142	TraesCS4B02G391500.1#domain1#PF00010	95.7%	24.0%	VKQLE--
143	TraesCS5A02G555200.1#domain1#PF00010	95.7%	24.0%	VKQLE--
144	TraesCS4B02G345800.1#domain1#PF00010	95.7%	24.0%	VKQLE--
145	TraesCS5A02G505100.1#domain1#PF00010	95.7%	26.0%	VKNLE--
146	TraesCS4B02G234700.1#domain1#PF00010	95.7%	26.0%	VKQLE--
147	TraesCS4D02G235900.1#domain1#PF00010	95.7%	26.0%	VKQLE--
148	TraesCS4B02G335200.1#domain1#PF00010	95.7%	26.0%	VKQLE--
149	TraesCS4D02G330500.1#domain1#PF00010	95.7%	26.0%	VKQLE--
150	TraesCS4B02G056600.2#domain1#PF00010	95.7%	28.0%	VKTLQ--
151	TraesCS4D02G056900.2#domain1#PF00010	95.7%	28.0%	VKTLQ--
152	TraesCS4A02G257900.2#domain1#PF00010	95.7%	28.0%	VKTLQ--
153	TraesCS4B02G021100.1#domain1#PF00010	95.7%	28.6%	VKELQ--
154	TraesCS4B02G021500.1#domain1#PF00010	95.7%	28.6%	VKELQ--
155	TraesCS4D02G019200.1#domain1#PF00010	95.7%	28.6%	VKELQ--
156	TraesCS4A02G292800.1#domain1#PF00010	95.7%	28.6%	VKELQ--
157	TraesCS4B02G020900.1#domain1#PF00010	95.7%	28.6%	AKELQ--
158	TraesCS4B02G021200.1#domain1#PF00010	95.7%	26.5%	VKELQ--
159	TraesCS4D02G018700.1#domain1#PF00010	95.7%	26.5%	VKELQ--
160	TraesCS3A02G019100.1#domain1#PF00010	95.7%	27.1%	VKELQ--
161	TraesCS3A02G019000.1#domain1#PF00010	95.7%	27.1%	VKELQ--
162	TraesCS3B02G029800.1#domain1#PF00010	95.7%	24.5%	VKQLQ--
163	TraesCS3D02G014900.1#domain1#PF00010	95.7%	24.5%	VKQLQ--
164	TraesCS4A02G408700.1#domain1#PF00010	95.7%	28.6%	VKELQ--
165	TraesCS4D02G306400.1#domain1#PF00010	95.7%	26.5%	VKELQ--
166	TraesCS4B02G308300.1#domain1#PF00010	95.7%	26.5%	VKELQ--
167	TraesCS4B02G308200.1#domain1#PF00010	95.7%	26.5%	VKELQ--
168	TraesCS4A02G408800.1#domain1#PF00010	95.7%	26.5%	VKELQ--
169	TraesCS4B02G308100.1#domain1#PF00010	93.6%	28.6%	VKELQ--
170	TraesCS4D02G306300.1#domain1#PF00010	93.6%	28.6%	VKELQ--
171	TraesCS7B02G483600.1#domain1#PF00010	95.7%	33.3%	LRELQ--
172	TraesCS7A02G558700.1#domain1#PF00010	95.7%	33.3%	VRELQ--
173	TraesCS5B02G518800.1#domain1#PF00010	95.7%	22.4%	VKEQQ--
174	TraesCSU02G075100.1#domain1#PF00010	91.5%	22.9%	VKE--
175	TraesCS5B02G013000.2#domain1#PF00010	95.7%	30.6%	VRELQ--
176	TraesCS5D02G020600.1#domain1#PF00010	95.7%	30.6%	VRELQ--
177	TraesCS5A02G014800.1#domain1#PF00010	95.7%	30.6%	VRELQ--
178	TraesCS4B02G020700.1#domain1#PF00010	95.7%	24.5%	VREQQ--
179	TraesCS4D02G018800.2#domain1#PF00010	95.7%	26.5%	VKEQQ--
180	TraesCS4D02G019100.1#domain1#PF00010	95.7%	26.5%	VKEQQ--
181	TraesCS4A02G292700.1#domain1#PF00010	95.7%	26.5%	VKEQQ--
182	TraesCSU02G075200.3#domain1#PF00010	95.7%	24.5%	VKEQQ--
183	TraesCS5B02G518500.1#domain1#PF00010	95.7%	24.5%	VKEQQ--
184	TraesCS5B02G518400.1#domain1#PF00010	95.7%	26.0%	VKEQQ--
185	TraesCS4A02G354100.1#domain1#PF00010	95.7%	26.0%	VKEQQ--
186	TraesCS5D02G517900.1#domain1#PF00010	95.7%	26.0%	VKEQQ--
187	TraesCS7A02G421000.1#domain1#PF00010	95.7%	25.5%	IPELQ--
188	TraesCS7B02G321400.1#domain1#PF00010	95.7%	25.5%	IPKLQ--
189	TraesCS3A02G489400.1#domain1#PF00010	95.7%	19.6%	IPELQ--
190	TraesCS3B02G549700.1#domain1#PF00010	95.7%	19.6%	IPELQ--
191	TraesCS3D02G495300.1#domain1#PF00010	95.7%	19.6%	IPELQ--
192	TraesCS3A02G489500.1#domain1#PF00010	95.7%	21.8%	IPELQ--
193	TraesCS3D02G495400.1#domain1#PF00010	95.7%	21.8%	IPELQ--
194	TraesCS3B02G549800.1#domain1#PF00010	95.7%	21.8%	IPELQ--
195	TraesCS2B02G543700.1#domain1#PF00010	95.7%	23.6%	IPELQ--
196	TraesCS2B02G543800.1#domain1#PF00010	95.7%	23.6%	IPELQ--
197	TraesCS2D02G517000.1#domain1#PF00010	95.7%	23.6%	IPELQ--
198	TraesCS2A02G515300.1#domain1#PF00010	95.7%	23.6%	IPELQ--
199	TraesCS3B02G550200.2#domain1#PF00010	95.7%	21.8%	IPELQ--
200	TraesCS3D02G495700.1#domain1#PF00010	95.7%	21.8%	IPELQ--
201	TraesCS3A02G489700.1#domain1#PF00010	95.7%	21.8%	IPELQ--
202	TraesCS3B02G550000.1#domain1#PF00010	95.7%	21.8%	IPELQ--
203	TraesCS3D02G495600.1#domain1#PF00010	95.7%	21.8%	IPELQ--
204	TraesCS3A02G489600.1#domain1#PF00010	93.6%	18.5%	IPEQ---
205	TraesCS6A02G114100.1#domain1#PF00010	95.7%	32.7%	IKSLE--
206	TraesCS6B02G142000.1#domain1#PF00010	95.7%	32.7%	IKSLE--

```
207 TraesCS2A02G442500.1#domain1#PF00010             87.2%  24.4%   IHEL----------------------------------------------------------------------------
208 TraesCS6D02G246100.2#domain1#PF00010             91.5%  26.0%   INEL----------------------------------------------------------------------------
209 TraesCS6B02G295900.2#domain1#PF00010             91.5%  26.0%   INEL----------------------------------------------------------------------------
210 TraesCS6A02G268800.1#domain1#PF00010             91.5%  26.0%   INEL----------------------------------------------------------------------------
211 TraesCS2D02G441800.1#domain1#PF00010             91.5%  28.6%   IDEL----------------------------------------------------------------------------
212 TraesCS2A02G442700.1#domain1#PF00010             91.5%  28.6%   IDEL----------------------------------------------------------------------------
213 TraesCS2B02G463800.1#domain1#PF00010             91.5%  28.6%   IDEL----------------------------------------------------------------------------
214 TraesCS6D02G185100.1#domain1#PF00010             91.5%  18.9%   LKDL----------------------------------------------------------------------------
215 TraesCS6B02G220300.1#domain1#PF00010             91.5%  18.9%   LKDL----------------------------------------------------------------------------
216 TraesCS6A02G204300.1#domain1#PF00010             91.5%  18.9%   LKDL----------------------------------------------------------------------------
217 TraesCS1D02G280600.1#domain1#PF00010             93.6%  18.0%   LDQL----------------------------------------------------------------------------
218 TraesCS1B02G290500.1#domain1#PF00010             93.6%  18.0%   LDQL----------------------------------------------------------------------------
219 TraesCS1A02G281300.1#domain1#PF00010             93.6%  18.0%   LDQL----------------------------------------------------------------------------
220 TraesCS6A02G045100.1#domain1#PF00010             93.6%  14.1%   LTQL----------------------------------------------------------------------------
221 TraesCS6D02G051600.2#domain1#PF00010             93.6%  14.1%   LTQL----------------------------------------------------------------------------
222 TraesCS6B02G060700.2#domain1#PF00010             93.6%  14.1%   LTQL----------------------------------------------------------------------------
223 TraesCS7A02G307700.1#domain1#PF00010             93.6%  17.6%   VTQL----------------------------------------------------------------------------
224 TraesCS7B02G208000.1#domain1#PF00010             93.6%  17.6%   VTQL----------------------------------------------------------------------------
225 TraesCS7D02G304500.1#domain1#PF00010             93.6%  17.6%   VTQL----------------------------------------------------------------------------
226 TraesCS2B02G240600.1#domain1#PF00010             93.6%  16.0%   VTEL----------------------------------------------------------------------------
227 TraesCS2A02G215600.1#domain1#PF00010             93.6%  16.0%   VTEL----------------------------------------------------------------------------
228 TraesCS2D02G221200.1#domain1#PF00010             93.6%  16.0%   VTEL----------------------------------------------------------------------------
229 TraesCS7D02G360600.1#domain1#PF00010             91.5%  26.5%   MAKL----------------------------------------------------------------------------
230 TraesCS7B02G265900.1#domain1#PF00010             91.5%  26.5%   MAKL----------------------------------------------------------------------------
231 TraesCS7A02G362500.1#domain1#PF00010             91.5%  26.5%   MARL----------------------------------------------------------------------------
232 TraesCS6A02G186200.1#domain1#PF00010             87.2%  21.3%   MN------------------------------------------------------------------------------
233 TraesCS6D02G173300.1#domain1#PF00010             87.2%  21.3%   MN------------------------------------------------------------------------------
234 TraesCS6B02G215200.1#domain1#PF00010             87.2%  21.3%   MN------------------------------------------------------------------------------
235 TraesCS5A02G251390.4#domain1#PF00010             95.7%  30.8%   IRFLQ---------------------------------------------------------------------------
236 TraesCS6A02G288100.3#domain1#PF00010             95.7%  36.5%   IRLLQ---------------------------------------------------------------------------
237 TraesCS6B02G317600.2#domain1#PF00010             95.7%  36.5%   IRLLQ---------------------------------------------------------------------------
238 TraesCS6D02G270900.1#domain1#PF00010             95.7%  36.5%   IRLLQ---------------------------------------------------------------------------
239 TraesCS5A02G466300.1#domain1#PF00010             91.5%  26.0%   VREL----------------------------------------------------------------------------
240 TraesCS5B02G478000.1#domain1#PF00010             91.5%  26.0%   VREL----------------------------------------------------------------------------
241 TraesCS5D02G479100.1#domain1#PF00010             91.5%  26.0%   VREL----------------------------------------------------------------------------
242 TraesCS1B02G129200.1#domain1#PF00010             91.5%  29.2%   VKEL----------------------------------------------------------------------------
243 TraesCS1D02G112000.1#domain1#PF00010             91.5%  29.2%   VKEL----------------------------------------------------------------------------
244 TraesCS1A02G110400.1#domain1#PF00010             91.5%  29.2%   VKEL----------------------------------------------------------------------------
245 TraesCS3A02G102900.1#domain1#PF00010             91.5%  28.6%   VRQL----------------------------------------------------------------------------
246 TraesCS3D02G105100.2#domain1#PF00010             91.5%  29.2%   VRHL----------------------------------------------------------------------------
247 TraesCS3B02G120200.1#domain1#PF00010             93.6%  30.6%   VKHLK---------------------------------------------------------------------------
248 TraesCS3B02G120500.1#domain1#PF00010             93.6%  30.6%   VKHLK---------------------------------------------------------------------------
249 TraesCS3A02G102600.1#domain1#PF00010             91.5%  29.2%   VRHL----------------------------------------------------------------------------
250 TraesCS2B02G494900.1#domain1#PF00010             93.6%  35.3%   VRELQ---------------------------------------------------------------------------
251 TraesCS2A02G472300.1#domain1#PF00010             93.6%  35.3%   VRELQ---------------------------------------------------------------------------
252 TraesCS2D02G472000.1#domain1#PF00010             93.6%  35.3%   VRELQ---------------------------------------------------------------------------
253 TraesCS5A02G245600.1#domain1#PF00010             91.5%  32.0%   VREL----------------------------------------------------------------------------
254 TraesCS5D02G252300.1#domain1#PF00010             91.5%  32.0%   VREL----------------------------------------------------------------------------
255 TraesCS5B02G243000.1#domain1#PF00010             91.5%  32.0%   VREL----------------------------------------------------------------------------
256 TraesCS5B02G229000.1#domain1#PF00010             91.5%  38.0%   VKEL----------------------------------------------------------------------------
257 TraesCS5A02G230500.1#domain1#PF00010             91.5%  38.0%   VKEL----------------------------------------------------------------------------
258 TraesCS5D02G237300.1#domain1#PF00010             91.5%  38.0%   VKEL----------------------------------------------------------------------------
259 TraesCS3A02G142900.1#domain1#PF00010             91.5%  32.7%   VKKL----------------------------------------------------------------------------
260 TraesCS3D02G144700.1#domain1#PF00010             91.5%  30.6%   VKKL----------------------------------------------------------------------------
261 TraesCS3A02G252900.1.cds1#domain2#PF00010        95.7%  24.5%   IQELE---------------------------------------------------------------------------
262 TraesCS3B02G284800.1.cds1#domain2#PF00010        95.7%  24.5%   IQELE---------------------------------------------------------------------------
263 TraesCS3D02G253700.1.cds1#domain2#PF00010        95.7%  24.5%   IQELE---------------------------------------------------------------------------
264 TraesCS3B02G288700.1#domain2#PF00010             95.7%  24.5%   IPELE---------------------------------------------------------------------------
265 5gnj:G                                           95.7%  17.3%   INELKSKVVKTESEKLQIKN---QLEEVK-LELAG--------------------------------------------
266 TraesCS1B02G208000.1.cds1#domain2#PF00010        93.6%  27.1%   INEL----------------------------------------------------------------------------
267 TraesCS1D02G196900.1.cds1#domain2#PF00010        93.6%  27.1%   INEL----------------------------------------------------------------------------
268 TraesCS1A02G193200.1#domain2#PF00010             93.6%  27.1%   INEL----------------------------------------------------------------------------
269 TraesCS3B02G185400.1#domain2#PF00010             95.7%  28.6%   ITDLQ---------------------------------------------------------------------------
270 TraesCS3D02G166300.2#domain2#PF00010             95.7%  28.6%   ITDLQ---------------------------------------------------------------------------
271 TraesCS3A02G158600.1.cds1#domain2#PF00010        95.7%  28.6%   ITDLQ---------------------------------------------------------------------------
272 TraesCS4A02G028900.1.cds1#domain2#PF00010        95.7%  30.6%   ITDLQ---------------------------------------------------------------------------
273 TraesCS4D02G275500.1.cds1#domain2#PF00010        95.7%  30.6%   ITDLQ---------------------------------------------------------------------------
274 TraesCS4B02G276900.1.cds1#domain2#PF00010        95.7%  30.6%   ITDLQ---------------------------------------------------------------------------
275 TraesCS2A02G448500.1.cds1#domain1#PF00010        93.6%  28.6%   IAEL----------------------------------------------------------------------------
276 TraesCS2B02G469200.1#domain1#PF00010             93.6%  28.6%   IAEL----------------------------------------------------------------------------
277 TraesCSU02G222600.1#domain1#PF00010              93.6%  28.6%   IAEL----------------------------------------------------------------------------
278 TraesCS2B02G469300.1.cds1#domain1#PF00010        93.6%  28.6%   IAEL----------------------------------------------------------------------------
279 TraesCS5A02G306600.1.cds1#domain1#PF00010        93.6%  27.1%   ITDL----------------------------------------------------------------------------
280 TraesCS5D02G313700.1.cds1#domain1#PF00010        93.6%  27.1%   ITDL----------------------------------------------------------------------------
281 TraesCS5B02G307100.1.cds1#domain1#PF00010        93.6%  29.2%   ITDL----------------------------------------------------------------------------
282 TraesCS5A02G306700.1.cds1#domain1#PF00010        93.6%  26.5%   ITDL----------------------------------------------------------------------------
283 TraesCS7D02G102200.1.cds1#domain1#PF00010        93.6%  24.5%   ITEL----------------------------------------------------------------------------
284 TraesCS1A02G369200.1.cds1#domain1#PF00010        93.6%  27.7%   IAEL----------------------------------------------------------------------------
285 TraesCS1D02G374800.1.cds1#domain1#PF00010        93.6%  27.7%   IGEL----------------------------------------------------------------------------
286 TraesCS5A02G306400.1.cds1#domain1#PF00010        93.6%  24.5%   IAEL----------------------------------------------------------------------------
287 TraesCS5B02G307000.1.cds1#domain1#PF00010        93.6%  24.5%   IAEL----------------------------------------------------------------------------
288 TraesCS5D02G313600.1.cds1#domain1#PF00010        93.6%  24.5%   IAEL----------------------------------------------------------------------------
289 TraesCS5A02G306200.1.cds1#domain1#PF00010        93.6%  24.5%   IAEL----------------------------------------------------------------------------
290 TraesCS5D02G313500.1.cds1#domain1#PF00010        93.6%  24.5%   IAEL----------------------------------------------------------------------------
291 TraesCS5B02G306800.1.cds1#domain1#PF00010        93.6%  24.5%   IAEL----------------------------------------------------------------------------
292 TraesCS5A02G306500.1.cds1#domain1#PF00010        93.6%  22.4%   IAEL----------------------------------------------------------------------------
293 TraesCS5B02G306600.1#domain1#PF00010             93.6%  25.0%   IAEL----------------------------------------------------------------------------
294 TraesCS5D02G313400.1.cds1#domain1#PF00010        93.6%  25.0%   IAEL----------------------------------------------------------------------------
295 TraesCS3D02G106600.2#domain1#PF00010             93.6%  25.0%   VNSL----------------------------------------------------------------------------
296 TraesCS3A02G104400.1#domain1#PF00010             93.6%  25.0%   VNSL----------------------------------------------------------------------------
297 TraesCS3B02G122800.1#domain1#PF00010             93.6%  25.0%   VNSL----------------------------------------------------------------------------
298 TraesCS3B02G122900.1#domain1#PF00010             95.7%  26.5%   VKSLK---------------------------------------------------------------------------
299 TraesCS3B02G236900.1#domain1#PF00010             95.7%  25.0%   LKTLE---------------------------------------------------------------------------
300 TraesCS3A02G204800.1#domain1#PF00010             95.7%  25.0%   LKTLE---------------------------------------------------------------------------
301 TraesCS3D02G210600.1#domain1#PF00010             95.7%  25.0%   LKTLE---------------------------------------------------------------------------
302 TraesCS4A02G087000.1#domain1#PF00010             93.6%  36.7%   VKEL----------------------------------------------------------------------------
303 TraesCS4B02G217400.1#domain1#PF00010             93.6%  36.7%   VKEL----------------------------------------------------------------------------
304 TraesCS4D02G217700.1#domain1#PF00010             93.6%  36.7%   VKEL----------------------------------------------------------------------------
305 TraesCS2A02G281200.1#domain1#PF00010             93.6%  36.2%   VKNLQ---------------------------------------------------------------------------
```

306	TraesCS2D02G280100.1#domain1#PF00010	93.6%	36.2%	VKNLQ---
307	TraesCS2B02G298600.1#domain1#PF00010	93.6%	36.2%	VKNLQ---
308	TraesCS3A02G442600.1#domain1#PF00010	85.1%	34.9%	IQQLQ---
309	TraesCS3D02G435200.1#domain1#PF00010	85.1%	32.6%	IHQLQ---
310	TraesCS2B02G289900.1#domain1#PF00010	95.7%	31.2%	IQQLQ---
311	TraesCS2D02G270300.1#domain1#PF00010	95.7%	31.2%	IQQLQ---
312	TraesCS2A02G271700.1#domain1#PF00010	95.7%	29.2%	IQQLH---
313	TraesCS4B02G364800.1#domain1#PF00010	95.7%	30.6%	IEELQ---
314	TraesCS5A02G533200.1#domain1#PF00010	95.7%	30.6%	IEELQ---
315	TraesCSU02G138900.1#domain1#PF00010	95.7%	30.6%	IEELQ---
316	TraesCS2D02G270000.2#domain1#PF00010	95.7%	32.7%	IEALQ---
317	TraesCS2B02G289700.2#domain1#PF00010	95.7%	32.7%	IEALQ---
318	TraesCS5A02G533900.1#domain1#PF00010	91.5%	30.4%	IEELQ---
319	TraesCSU02G138400.2#domain1#PF00010	91.5%	30.4%	IEELQ---
320	TraesCS3B02G018400.1#domain1#PF00010	95.7%	28.8%	IEELQ---
321	TraesCS3A02G028200.3#domain1#PF00010	95.7%	28.8%	IEELQ---
322	TraesCS3D02G017700.1#domain1#PF00010	95.7%	28.8%	IEELQ---
323	TraesCS2B02G237300.1#domain1#PF00010	95.7%	40.8%	IKKLQ---
324	TraesCS2A02G212200.1#domain1#PF00010	95.7%	38.8%	IKKLQ---
325	TraesCS2D02G218100.1#domain1#PF00010	95.7%	38.8%	IKKLQ---
326	TraesCS4D02G224600.1#domain2#PF00010	95.7%	4.9%	LRELREEWTDGAISSLLDAYM-DRFEQLNRGNLRGRDWEDVAAAVTDGQGKSSGGKSVEQCKNKIDNLKKRYKVECQRIA
327	TraesCS2D02G575600.3#domain2#PF00010	95.7%	20.4%	LKELE---
328	TraesCSU02G029400.1#domain2#PF00010	95.7%	22.4%	LKELE---
329	TraesCS4B02G397400.1#domain2#PF00010	95.7%	22.4%	LKELE---
330	TraesCS5A02G558500.1#domain2#PF00010	95.7%	20.4%	LRELE---
331	TraesCS5A02G489500.1#domain2#PF00010	95.7%	20.4%	LRELE---
332	TraesCS4B02G397300.1#domain1#PF00010	95.7%	18.4%	LRELE---
333	TraesCS2D02G406900.1#domain2#PF00010	95.7%	22.0%	LRELE---
334	TraesCS2B02G428000.1#domain2#PF00010	95.7%	22.0%	LRELE---
335	TraesCS2A02G409600.2#domain2#PF00010	95.7%	22.0%	LRELE---
336	TraesCSU02G228400.1#domain1#PF00010	95.7%	22.0%	LRELE---
337	TraesCSU02G237100.1#domain1#PF00010	95.7%	22.0%	LRELE---
338	TraesCS2A02G409400.1#domain2#PF00010	95.7%	22.0%	LRELE---
339	TraesCSU02G116000.1#domain2#PF00010	95.7%	26.5%	LKELE---
340	TraesCS4B02G397800.1#domain2#PF00010	95.7%	26.5%	LKELE---
341	TraesCS1B02G112900.1#domain2#PF00010	97.9%	20.0%	VKQL--
342	TraesCS1B02G113100.4#domain2#PF00010	95.7%	27.1%	VKQL--
343	TraesCS1A02G102400.2#domain2#PF00010	95.7%	27.1%	VKQL--
344	TraesCS6B02G095600.1#domain1#PF00010	91.5%	39.1%	IVGLQ---
345	TraesCS6D02G069300.1#domain1#PF00010	91.5%	41.3%	IVGLQ---
346	TraesCS6A02G071200.1#domain1#PF00010	91.5%	41.3%	IVGLQ---
347	TraesCS6B02G095700.1#domain1#PF00010	91.5%	39.1%	IVGLQ---
348	TraesCS6D02G069400.1#domain1#PF00010	91.5%	39.1%	IVGLQ---
349	TraesCS6A02G071300.1#domain1#PF00010	91.5%	39.1%	IVGLQ---
350	TraesCS4B02G051500.2#domain1#PF00010	89.4%	33.3%	LKEL--
351	TraesCS4D02G051600.2#domain1#PF00010	89.4%	33.3%	LKEL--
352	TraesCS4A02G263300.2#domain1#PF00010	89.4%	33.3%	LKEL--
353	TraesCS3B02G476000.1#domain1#PF00010	89.4%	31.1%	LKEL--
354	TraesCS3D02G434900.1#domain1#PF00010	89.4%	31.1%	LKEL--
355	TraesCS3A02G442200.1#domain1#PF00010	89.4%	31.1%	LKEL--
356	TraesCS7A02G311800.1#domain1#PF00010	89.4%	33.3%	LKEL--
357	TraesCS7B02G211600.1#domain1#PF00010	89.4%	33.3%	LKEL--
358	TraesCS7D02G308300.1#domain1#PF00010	89.4%	33.3%	LKEL--
359	TraesCS3A02G057900.1#domain1#PF00010	89.4%	31.1%	VKEL--
360	TraesCS3D02G058100.1#domain1#PF00010	89.4%	28.9%	VKEL--
361	TraesCS7A02G543300.1#domain1#PF00010	89.4%	31.1%	MKEL--
362	TraesCS7D02G529200.1#domain1#PF00010	89.4%	31.1%	MKEL--
363	TraesCS7B02G465400.1#domain1#PF00010	89.4%	31.1%	MKEL--
364	TraesCSU02G069400.1#domain1#PF00010	91.5%	28.3%	VNEL--
365	TraesCSU02G069300.1#domain1#PF00010	91.5%	28.3%	VNEL--
366	TraesCS4D02G328800.1#domain1#PF00010	89.4%	26.7%	VNEL--
367	TraesCS4B02G331900.1#domain1#PF00010	89.4%	28.9%	VNEL--
368	TraesCS4B02G332000.1#domain1#PF00010	93.6%	26.0%	VNEL--
369	TraesCS5A02G503400.1#domain1#PF00010	89.4%	28.9%	VNEL--
370	TraesCS4D02G328900.1#domain1#PF00010	89.4%	28.9%	VNEL--
371	TraesCS4D02G329100.1#domain1#PF00010	89.4%	28.9%	VNEL--
372	TraesCS5A02G503300.1#domain1#PF00010	89.4%	28.9%	VNEL--
373	TraesCS5A02G503500.1#domain1#PF00010	89.4%	28.9%	VNEL--
374	TraesCS4D02G329000.2#domain1#PF00010	89.4%	28.9%	VNEL--
	consensus/100%			h...
	consensus/90%			lt.L..
	consensus/80%			lptL..
	consensus/70%			lcpL..

		cov	pid	161 . . . 2 240
1	TraesCS6B02G244800.1#domain1#PF00010	100.0%	100.0%	--
2	TraesCS6A02G214900.1#domain1#PF00010	100.0%	100.0%	--
3	TraesCS6D02G197500.1#domain1#PF00010	100.0%	97.9%	--
4	TraesCS3D02G388700.1#domain1#PF00010	100.0%	83.0%	--
5	TraesCS3B02G426900.1#domain1#PF00010	100.0%	83.0%	--
6	TraesCS3A02G395000.1#domain1#PF00010	100.0%	83.0%	--
7	TraesCS7A02G245800.1#domain1#PF00010	100.0%	78.7%	--
8	TraesCS7D02G244300.1#domain1#PF00010	100.0%	78.7%	--
9	TraesCS7B02G145800.1#domain1#PF00010	100.0%	78.7%	--
10	TraesCS5A02G264800.1#domain1#PF00010	100.0%	76.6%	--
11	TraesCS5D02G272800.1#domain1#PF00010	100.0%	76.6%	--
12	TraesCS5B02G264300.1#domain1#PF00010	100.0%	76.6%	--
13	TraesCS4B02G317900.1#domain1#PF00010	97.9%	37.5%	--
14	TraesCS3B02G010400.1#domain1#PF00010	93.6%	44.4%	--
15	TraesCS3B02G007700.1#domain1#PF00010	93.6%	44.4%	--
16	TraesCS3D02G004300.1#domain1#PF00010	93.6%	44.4%	--
17	TraesCS3A02G006300.1#domain1#PF00010	87.2%	47.6%	--
18	TraesCS6A02G276200.1#domain1#PF00010	87.2%	45.2%	--
19	TraesCS6B02G303600.1#domain1#PF00010	89.4%	46.5%	--
20	TraesCS6D02G256600.1#domain1#PF00010	89.4%	46.5%	--
21	TraesCS6D02G256400.1#domain1#PF00010	89.4%	44.2%	--
22	TraesCS6D02G256500.1#domain1#PF00010	87.2%	45.2%	--
23	TraesCS6A02G276100.1#domain1#PF00010	87.2%	45.2%	--
24	TraesCS6B02G303500.1#domain1#PF00010	87.2%	45.2%	--

25	TraesCS6D02G256300.1#domain1#PF00010	87.2%	45.2%	--
26	TraesCS4B02G257200.1#domain1#PF00010	87.2%	45.2%	--
27	TraesCS4A02G047700.1#domain1#PF00010	87.2%	45.2%	--
28	TraesCS4D02G257100.1#domain1#PF00010	87.2%	45.2%	--
29	TraesCS5A02G067600.1#domain1#PF00010	87.2%	50.0%	--
30	TraesCS5D02G078800.1#domain1#PF00010	87.2%	50.0%	--
31	TraesCS5B02G074500.1#domain1#PF00010	87.2%	52.4%	--
32	TraesCS2A02G194200.1#domain1#PF00010	87.2%	50.0%	--
33	TraesCS2B02G212700.1#domain1#PF00010	87.2%	50.0%	--
34	TraesCS2D02G193700.1#domain1#PF00010	87.2%	50.0%	--
35	TraesCS4A02G234500.1#domain1#PF00010	87.2%	50.0%	--
36	TraesCS4D02G079500.1#domain1#PF00010	87.2%	50.0%	--
37	TraesCS4B02G080700.1#domain1#PF00010	87.2%	50.0%	--
38	TraesCS5B02G406100.1#domain1#PF00010	87.2%	52.4%	--
39	TraesCS5D02G411600.1#domain1#PF00010	87.2%	50.0%	--
40	TraesCS5A02G401300.1#domain1#PF00010	87.2%	50.0%	--
41	TraesCS1B02G359000.1#domain1#PF00010	85.1%	43.9%	--
42	TraesCS1A02G345200.1#domain1#PF00010	85.1%	43.9%	--
43	TraesCS1D02G347900.1#domain1#PF00010	85.1%	43.9%	--
44	TraesCS3A02G440600.1#domain1#PF00010	89.4%	48.8%	--
45	TraesCS3D02G433200.1#domain1#PF00010	89.4%	48.8%	--
46	TraesCS3B02G474700.1#domain1#PF00010	87.2%	50.0%	--
47	TraesCS3A02G350600.1.cds1#domain1#PF00010	85.1%	48.8%	--
48	TraesCS3B02G383000.1.cds1#domain1#PF00010	85.1%	48.8%	--
49	TraesCS3D02G344600.1.cds1#domain1#PF00010	85.1%	48.8%	--
50	TraesCS7B02G160500.1.cds1#domain1#PF00010	87.2%	21.7%	--
51	TraesCS7A02G340300.1#domain1#PF00010	100.0%	32.0%	--
52	TraesCS7B02G251900.1#domain1#PF00010	100.0%	32.0%	--
53	TraesCS7D02G347900.1#domain1#PF00010	100.0%	32.0%	--
54	TraesCS1B02G226200.2#domain1#PF00010	97.9%	46.8%	--
55	TraesCS1D02G215600.2#domain1#PF00010	97.9%	46.8%	--
56	TraesCS1A02G212700.1#domain1#PF00010	97.9%	46.8%	--
57	TraesCS2D02G461900.1#domain1#PF00010	97.9%	42.6%	--
58	TraesCS2D02G461700.3#domain1#PF00010	97.9%	42.9%	--
59	TraesCS2A02G461700.2#domain1#PF00010	97.9%	42.9%	--
60	TraesCS2B02G483300.1#domain1#PF00010	97.9%	42.9%	--
61	TraesCS1B02G100400.2#domain1#PF00010	97.9%	43.8%	--
62	TraesCS1A02G083000.1#domain1#PF00010	97.9%	43.8%	--
63	TraesCS1D02G084200.1#domain1#PF00010	97.9%	43.8%	--
64	TraesCS5B02G054800.2#domain1#PF00010	97.9%	46.9%	--
65	TraesCS5A02G049600.1#domain1#PF00010	97.9%	46.9%	--
66	TraesCS5D02G060300.1#domain1#PF00010	97.9%	46.9%	--
67	TraesCS5A02G376500.1#domain1#PF00010	97.9%	45.8%	--
68	TraesCS5B02G380200.1#domain1#PF00010	97.9%	45.8%	--
69	TraesCS5D02G386500.1#domain1#PF00010	97.9%	45.8%	--
70	TraesCS2A02G253900.2#domain1#PF00010	97.9%	41.7%	--
71	TraesCS2B02G273500.1#domain1#PF00010	97.9%	41.7%	--
72	TraesCS2D02G254400.3#domain1#PF00010	97.9%	41.7%	--
73	TraesCS5A02G420200.1#domain1#PF00010	97.9%	41.7%	--
74	TraesCS5B02G422000.1#domain1#PF00010	97.9%	41.7%	--
75	TraesCS5D02G428400.2#domain1#PF00010	97.9%	41.7%	--
76	TraesCS6B02G411300.1#domain1#PF00010	97.9%	41.7%	--
77	TraesCS6A02G373500.2#domain1#PF00010	97.9%	43.8%	--
78	TraesCS6D02G357700.1#domain1#PF00010	97.9%	43.8%	--
79	TraesCS7A02G126900.1#domain1#PF00010	97.9%	43.8%	--
80	TraesCS7D02G124700.1#domain1#PF00010	97.9%	43.8%	--
81	TraesCS7B02G026300.1#domain1#PF00010	97.9%	43.8%	--
82	TraesCS6A02G190600.1#domain1#PF00010	95.7%	42.9%	--
83	TraesCS7A02G323000.1.cds1#domain1#PF00010	85.1%	51.2%	--
84	TraesCS7D02G319600.1.cds1#domain1#PF00010	85.1%	51.2%	--
85	TraesCS7B02G223900.1.cds1#domain1#PF00010	85.1%	51.2%	--
86	TraesCS7D02G263400.1.cds1#domain1#PF00010	85.1%	48.8%	--
87	TraesCS7A02G262600.1.cds1#domain1#PF00010	85.1%	48.8%	--
88	TraesCS7B02G303300.1#domain1#PF00010	100.0%	35.4%	--
89	TraesCS7D02G397300.1#domain1#PF00010	100.0%	35.4%	--
90	TraesCS7A02G157700.1#domain1#PF00010	97.9%	51.1%	--
91	TraesCS7D02G158300.1#domain1#PF00010	97.9%	51.1%	--
92	TraesCS7B02G062100.2#domain1#PF00010	97.9%	51.1%	--
93	TraesCS7A02G147500.1#domain1#PF00010	97.9%	48.9%	--
94	TraesCS7D02G149400.1#domain1#PF00010	97.9%	48.9%	--
95	TraesCS6B02G391100.1#domain1#PF00010	97.9%	46.8%	--
96	TraesCS6D02G341200.1#domain1#PF00010	97.9%	46.8%	--
97	TraesCS6A02G358400.3#domain1#PF00010	97.9%	48.9%	--
98	TraesCS5A02G225700.1#domain1#PF00010	97.9%	48.9%	--
99	TraesCS5D02G233000.1#domain1#PF00010	100.0%	46.9%	--
100	TraesCS5B02G224200.1#domain1#PF00010	100.0%	46.9%	--
101	TraesCS5D02G300100.1#domain1#PF00010	97.9%	40.8%	--
102	TraesCS5B02G292100.1#domain1#PF00010	97.9%	40.8%	--
103	TraesCS5D02G459800.1#domain1#PF00010	97.9%	32.7%	--
104	TraesCS5B02G457600.1#domain1#PF00010	97.9%	32.7%	--
105	TraesCS4D02G237100.3#domain1#PF00010	100.0%	40.0%	--
106	TraesCS4B02G235700.1#domain1#PF00010	97.9%	38.0%	--
107	TraesCS4A02G060200.2#domain1#PF00010	97.9%	38.0%	--
108	TraesCS4A02G255800.3#domain1#PF00010	97.9%	42.0%	--
109	TraesCS4D02G058900.1#domain1#PF00010	97.9%	40.0%	--
110	TraesCS4B02G059000.1#domain1#PF00010	97.9%	40.0%	--
111	TraesCS7D02G327900.1#domain1#PF00010	95.7%	24.5%	--
112	TraesCS7A02G350900.1#domain1#PF00010	95.7%	24.5%	--
113	TraesCS7B02G232000.1#domain1#PF00010	95.7%	24.5%	--
114	TraesCS4B02G347100.1#domain1#PF00010	95.7%	22.2%	--
115	TraesCS5A02G515800.1#domain1#PF00010	95.7%	22.2%	--
116	TraesCS4D02G342000.1#domain1#PF00010	95.7%	22.2%	--
117	TraesCS2B02G141600.1#domain1#PF00010	93.6%	24.5%	--
118	TraesCS2D02G122800.1#domain1#PF00010	93.6%	26.0%	--
119	TraesCS2A02G120700.1#domain1#PF00010	93.6%	26.0%	--
120	TraesCS6B02G346100.1#domain1#PF00010	95.7%	20.4%	--
121	TraesCS6D02G294900.1#domain1#PF00010	95.7%	20.4%	--
122	TraesCS6A02G315700.1#domain1#PF00010	95.7%	20.4%	--
123	TraesCS1B02G300100.1#domain1#PF00010	95.7%	25.9%	--

124	TraesCS1D02G289400.1#domain1#PF00010	95.7%	25.9%	--
125	TraesCS1A02G290800.1#domain1#PF00010	95.7%	25.9%	--
126	TraesCS6D02G315200.1#domain1#PF00010	95.7%	20.4%	--
127	TraesCS6A02G335500.1#domain1#PF00010	95.7%	20.4%	--
128	TraesCS6B02G366200.1#domain1#PF00010	95.7%	20.4%	--
129	TraesCS6A02G264200.1#domain1#PF00010	95.7%	20.4%	--
130	TraesCS6B02G291200.1#domain1#PF00010	95.7%	20.4%	--
131	TraesCS6D02G250700.1#domain1#PF00010	95.7%	20.4%	--
132	TraesCS2B02G469700.1#domain1#PF00010	95.7%	20.4%	--
133	TraesCS2B02G469800.1#domain1#PF00010	95.7%	20.4%	--
134	TraesCS2D02G448000.1#domain1#PF00010	95.7%	20.4%	--
135	TraesCS2A02G448800.1#domain1#PF00010	95.7%	20.4%	--
136	TraesCS4B02G304500.1#domain1#PF00010	95.7%	20.4%	--
137	TraesCS4A02G404700.1#domain1#PF00010	95.7%	20.4%	--
138	TraesCS4D02G302700.1#domain1#PF00010	95.7%	20.4%	--
139	TraesCS5B02G245900.1#domain1#PF00010	95.7%	20.4%	--
140	TraesCS5A02G248200.1#domain1#PF00010	95.7%	20.4%	--
141	TraesCS5D02G255100.1#domain1#PF00010	95.7%	20.4%	--
142	TraesCS4B02G391500.1#domain1#PF00010	95.7%	24.0%	--
143	TraesCS5A02G555200.1#domain1#PF00010	95.7%	24.0%	--
144	TraesCS4B02G345800.1#domain1#PF00010	95.7%	24.0%	--
145	TraesCS5A02G505100.1#domain1#PF00010	95.7%	26.0%	--
146	TraesCS4B02G234700.1#domain1#PF00010	95.7%	26.0%	--
147	TraesCS4D02G235900.1#domain1#PF00010	95.7%	26.0%	--
148	TraesCS4B02G335200.1#domain1#PF00010	95.7%	26.0%	--
149	TraesCS4D02G330500.1#domain1#PF00010	95.7%	26.0%	--
150	TraesCS4B02G056600.2#domain1#PF00010	95.7%	28.0%	--
151	TraesCS4D02G056900.2#domain1#PF00010	95.7%	28.0%	--
152	TraesCS4A02G257900.2#domain1#PF00010	95.7%	28.0%	--
153	TraesCS4B02G021100.1#domain1#PF00010	95.7%	28.6%	--
154	TraesCS4B02G021500.1#domain1#PF00010	95.7%	28.6%	--
155	TraesCS4D02G019200.1#domain1#PF00010	95.7%	28.6%	--
156	TraesCS4A02G292800.1#domain1#PF00010	95.7%	28.6%	--
157	TraesCS4B02G020900.1#domain1#PF00010	95.7%	28.6%	--
158	TraesCS4B02G021200.1#domain1#PF00010	95.7%	26.5%	--
159	TraesCS4D02G018700.1#domain1#PF00010	95.7%	26.5%	--
160	TraesCS3A02G019100.1#domain1#PF00010	95.7%	27.1%	--
161	TraesCS3A02G019000.1#domain1#PF00010	95.7%	27.1%	--
162	TraesCS3B02G029800.1#domain1#PF00010	95.7%	24.5%	--
163	TraesCS3D02G014900.1#domain1#PF00010	95.7%	24.5%	--
164	TraesCS4A02G408700.1#domain1#PF00010	95.7%	28.6%	--
165	TraesCS4D02G306400.1#domain1#PF00010	95.7%	26.5%	--
166	TraesCS4B02G308300.1#domain1#PF00010	95.7%	26.5%	--
167	TraesCS4B02G308200.1#domain1#PF00010	95.7%	26.5%	--
168	TraesCS4A02G408800.1#domain1#PF00010	95.7%	26.5%	--
169	TraesCS4B02G308100.1#domain1#PF00010	93.6%	28.6%	--
170	TraesCS4D02G306300.1#domain1#PF00010	93.6%	28.6%	--
171	TraesCS7B02G483600.1#domain1#PF00010	95.7%	33.3%	--
172	TraesCS7A02G558700.1#domain1#PF00010	95.7%	33.3%	--
173	TraesCS5B02G518800.1#domain1#PF00010	95.7%	22.4%	--
174	TraesCSU02G075100.1#domain1#PF00010	91.5%	22.9%	--
175	TraesCS5B02G013000.2#domain1#PF00010	95.7%	30.6%	--
176	TraesCS5D02G020600.1#domain1#PF00010	95.7%	30.6%	--
177	TraesCS5A02G014800.1#domain1#PF00010	95.7%	30.6%	--
178	TraesCS4B02G020700.1#domain1#PF00010	95.7%	24.5%	--
179	TraesCS4D02G018800.2#domain1#PF00010	95.7%	26.5%	--
180	TraesCS4D02G019100.1#domain1#PF00010	95.7%	26.5%	--
181	TraesCS4A02G292700.1#domain1#PF00010	95.7%	26.5%	--
182	TraesCSU02G075200.3#domain1#PF00010	95.7%	24.5%	--
183	TraesCS5B02G518500.1#domain1#PF00010	95.7%	24.5%	--
184	TraesCS5B02G518400.1#domain1#PF00010	95.7%	26.0%	--
185	TraesCS4A02G354100.1#domain1#PF00010	95.7%	26.0%	--
186	TraesCS5D02G517900.1#domain1#PF00010	95.7%	26.0%	--
187	TraesCS7A02G421000.1#domain1#PF00010	95.7%	25.5%	--
188	TraesCS7B02G321400.1#domain1#PF00010	95.7%	25.5%	--
189	TraesCS3A02G489400.1#domain1#PF00010	95.7%	19.6%	--
190	TraesCS3B02G549700.1#domain1#PF00010	95.7%	19.6%	--
191	TraesCS3D02G495300.1#domain1#PF00010	95.7%	19.6%	--
192	TraesCS3A02G489500.1#domain1#PF00010	95.7%	21.8%	--
193	TraesCS3D02G495400.1#domain1#PF00010	95.7%	21.8%	--
194	TraesCS3B02G549800.1#domain1#PF00010	95.7%	21.8%	--
195	TraesCS2B02G543700.1#domain1#PF00010	95.7%	23.6%	--
196	TraesCS2B02G543800.1#domain1#PF00010	95.7%	23.6%	--
197	TraesCS2D02G517000.1#domain1#PF00010	95.7%	23.6%	--
198	TraesCS2A02G515300.1#domain1#PF00010	95.7%	23.6%	--
199	TraesCS3B02G550200.2#domain1#PF00010	95.7%	21.8%	--
200	TraesCS3D02G495700.1#domain1#PF00010	95.7%	21.8%	--
201	TraesCS3A02G489700.1#domain1#PF00010	95.7%	21.8%	--
202	TraesCS3B02G550000.1#domain1#PF00010	95.7%	21.8%	--
203	TraesCS3D02G495600.1#domain1#PF00010	95.7%	21.8%	--
204	TraesCS3A02G489600.1#domain1#PF00010	93.6%	18.5%	--
205	TraesCS6A02G114100.1#domain1#PF00010	95.7%	32.7%	--
206	TraesCS6B02G142000.1#domain1#PF00010	95.7%	32.7%	--
207	TraesCS2A02G442500.1#domain1#PF00010	87.2%	24.4%	--
208	TraesCS6D02G246100.2#domain1#PF00010	91.5%	26.0%	--
209	TraesCS6B02G295900.2#domain1#PF00010	91.5%	26.0%	--
210	TraesCS6A02G268800.1#domain1#PF00010	91.5%	26.0%	--
211	TraesCS2D02G441800.1#domain1#PF00010	91.5%	28.6%	--
212	TraesCS2A02G442700.1#domain1#PF00010	91.5%	28.6%	--
213	TraesCS2B02G463800.1#domain1#PF00010	91.5%	28.6%	--
214	TraesCS6D02G185100.1#domain1#PF00010	91.5%	18.9%	--
215	TraesCS6B02G220300.1#domain1#PF00010	91.5%	18.9%	--
216	TraesCS6A02G204300.1#domain1#PF00010	91.5%	18.9%	--
217	TraesCS1D02G280600.1#domain1#PF00010	93.6%	18.0%	--
218	TraesCS1B02G290500.1#domain1#PF00010	93.6%	18.0%	--
219	TraesCS1A02G281300.1#domain1#PF00010	93.6%	18.0%	--
220	TraesCS6A02G045100.1#domain1#PF00010	93.6%	14.1%	--
221	TraesCS6D02G051600.2#domain1#PF00010	93.6%	14.1%	--
222	TraesCS6B02G060700.2#domain1#PF00010	93.6%	14.1%	--

223	TraesCS7A02G307700.1#domain1#PF00010	93.6%	17.6%	--
224	TraesCS7B02G208000.1#domain1#PF00010	93.6%	17.6%	--
225	TraesCS7D02G304500.1#domain1#PF00010	93.6%	17.6%	--
226	TraesCS2B02G240600.1#domain1#PF00010	93.6%	16.0%	--
227	TraesCS2A02G215600.1#domain1#PF00010	93.6%	16.0%	--
228	TraesCS2D02G221200.1#domain1#PF00010	93.6%	16.0%	--
229	TraesCS7D02G360600.1#domain1#PF00010	91.5%	26.5%	--
230	TraesCS7B02G265900.1#domain1#PF00010	91.5%	26.5%	--
231	TraesCS7A02G362500.1#domain1#PF00010	91.5%	26.5%	--
232	TraesCS6A02G186200.1#domain1#PF00010	87.2%	21.3%	--
233	TraesCS6D02G173300.1#domain1#PF00010	87.2%	21.3%	--
234	TraesCS6B02G215200.1#domain1#PF00010	87.2%	21.3%	--
235	TraesCS5A02G251390.4#domain1#PF00010	95.7%	30.8%	--
236	TraesCS6A02G288100.3#domain1#PF00010	95.7%	36.5%	--
237	TraesCS6B02G317600.2#domain1#PF00010	95.7%	36.5%	--
238	TraesCS6D02G270900.1#domain1#PF00010	95.7%	36.5%	--
239	TraesCS5A02G466300.1#domain1#PF00010	91.5%	26.0%	--
240	TraesCS5B02G478000.1#domain1#PF00010	91.5%	26.0%	--
241	TraesCS5D02G479100.1#domain1#PF00010	91.5%	26.0%	--
242	TraesCS1B02G129200.1#domain1#PF00010	91.5%	29.2%	--
243	TraesCS1D02G112000.1#domain1#PF00010	91.5%	29.2%	--
244	TraesCS1A02G110400.1#domain1#PF00010	91.5%	29.2%	--
245	TraesCS3A02G102900.1#domain1#PF00010	91.5%	28.6%	--
246	TraesCS3D02G105100.2#domain1#PF00010	91.5%	29.2%	--
247	TraesCS3B02G120200.1#domain1#PF00010	93.6%	30.6%	--
248	TraesCS3B02G120500.1#domain1#PF00010	93.6%	30.6%	--
249	TraesCS3A02G102600.1#domain1#PF00010	91.5%	29.2%	--
250	TraesCS2B02G494900.1#domain1#PF00010	93.6%	35.3%	--
251	TraesCS2A02G472300.1#domain1#PF00010	93.6%	35.3%	--
252	TraesCS2D02G472000.1#domain1#PF00010	93.6%	35.3%	--
253	TraesCS5A02G245600.1#domain1#PF00010	91.5%	32.0%	--
254	TraesCS5D02G252300.1#domain1#PF00010	91.5%	32.0%	--
255	TraesCS5B02G243000.1#domain1#PF00010	91.5%	32.0%	--
256	TraesCS5B02G229000.1#domain1#PF00010	91.5%	38.0%	--
257	TraesCS5A02G230500.1#domain1#PF00010	91.5%	38.0%	--
258	TraesCS5D02G237300.1#domain1#PF00010	91.5%	38.0%	--
259	TraesCS3A02G142900.1#domain1#PF00010	91.5%	32.7%	--
260	TraesCS3D02G144700.1#domain1#PF00010	91.5%	30.6%	--
261	TraesCS3A02G252900.1.cds1#domain2#PF00010	95.7%	24.5%	--
262	TraesCS3B02G284800.1.cds1#domain2#PF00010	95.7%	24.5%	--
263	TraesCS3D02G253700.1.cds1#domain2#PF00010	95.7%	24.5%	--
264	TraesCS3B02G288700.1#domain2#PF00010	95.7%	24.5%	--
265	5gnj:G	95.7%	17.3%	--
266	TraesCS1B02G208000.1.cds1#domain2#PF00010	93.6%	27.1%	--
267	TraesCS1D02G196900.1.cds1#domain2#PF00010	93.6%	27.1%	--
268	TraesCS1A02G193200.1#domain2#PF00010	93.6%	27.1%	--
269	TraesCS3B02G185400.1#domain2#PF00010	95.7%	28.6%	--
270	TraesCS3D02G166300.2#domain2#PF00010	95.7%	28.6%	--
271	TraesCS3A02G158600.1.cds1#domain2#PF00010	95.7%	28.6%	--
272	TraesCS4A02G028900.1.cds1#domain2#PF00010	95.7%	30.6%	--
273	TraesCS4D02G275500.1.cds1#domain2#PF00010	95.7%	30.6%	--
274	TraesCS4B02G276900.1.cds1#domain2#PF00010	95.7%	30.6%	--
275	TraesCS2A02G448500.1.cds1#domain1#PF00010	93.6%	28.6%	--
276	TraesCS2B02G469200.1#domain1#PF00010	93.6%	28.6%	--
277	TraesCSU02G222600.1#domain1#PF00010	93.6%	28.6%	--
278	TraesCS2B02G469300.1.cds1#domain1#PF00010	93.6%	28.6%	--
279	TraesCS5A02G306600.1.cds1#domain1#PF00010	93.6%	27.1%	--
280	TraesCS5D02G313700.1.cds1#domain1#PF00010	93.6%	27.1%	--
281	TraesCS5B02G307100.1.cds1#domain1#PF00010	93.6%	29.2%	--
282	TraesCS5A02G306700.1.cds1#domain1#PF00010	93.6%	26.5%	--
283	TraesCS7D02G102200.1.cds1#domain1#PF00010	93.6%	24.5%	--
284	TraesCS1A02G369200.1.cds1#domain1#PF00010	93.6%	27.7%	--
285	TraesCS1D02G374800.1.cds1#domain1#PF00010	93.6%	27.7%	--
286	TraesCS5A02G306400.1.cds1#domain1#PF00010	93.6%	24.5%	--
287	TraesCS5B02G307000.1.cds1#domain1#PF00010	93.6%	24.5%	--
288	TraesCS5D02G313600.1.cds1#domain1#PF00010	93.6%	24.5%	--
289	TraesCS5A02G306200.1.cds1#domain1#PF00010	93.6%	24.5%	--
290	TraesCS5D02G313500.1.cds1#domain1#PF00010	93.6%	24.5%	--
291	TraesCS5B02G306800.1.cds1#domain1#PF00010	93.6%	24.5%	--
292	TraesCS5A02G306500.1.cds1#domain1#PF00010	93.6%	22.4%	--
293	TraesCS5B02G306600.1#domain1#PF00010	93.6%	25.0%	--
294	TraesCS5D02G313400.1.cds1#domain1#PF00010	93.6%	25.0%	--
295	TraesCS3D02G106600.2#domain1#PF00010	93.6%	25.0%	--
296	TraesCS3A02G104400.1#domain1#PF00010	93.6%	25.0%	--
297	TraesCS3B02G122800.1#domain1#PF00010	93.6%	25.0%	--
298	TraesCS3B02G122900.1#domain1#PF00010	95.7%	26.5%	--
299	TraesCS3B02G236900.1#domain1#PF00010	95.7%	25.0%	--
300	TraesCS3A02G204800.1#domain1#PF00010	95.7%	25.0%	--
301	TraesCS3D02G210600.1#domain1#PF00010	95.7%	25.0%	--
302	TraesCS4A02G087000.1#domain1#PF00010	93.6%	36.7%	--
303	TraesCS4B02G217400.1#domain1#PF00010	93.6%	36.7%	--
304	TraesCS4D02G217700.1#domain1#PF00010	93.6%	36.7%	--
305	TraesCS2A02G281200.1#domain1#PF00010	93.6%	36.2%	--
306	TraesCS2D02G280100.1#domain1#PF00010	93.6%	36.2%	--
307	TraesCS2B02G298600.1#domain1#PF00010	93.6%	36.2%	--
308	TraesCS3A02G442600.1#domain1#PF00010	85.1%	34.9%	--
309	TraesCS3D02G435200.1#domain1#PF00010	85.1%	32.6%	--
310	TraesCS2B02G289900.1#domain1#PF00010	95.7%	31.2%	--
311	TraesCS2D02G270300.1#domain1#PF00010	95.7%	31.2%	--
312	TraesCS2A02G271700.1#domain1#PF00010	95.7%	29.2%	--
313	TraesCS4B02G364800.1#domain1#PF00010	95.7%	30.6%	--
314	TraesCS5A02G533200.1#domain1#PF00010	95.7%	30.6%	--
315	TraesCSU02G138900.1#domain1#PF00010	95.7%	30.6%	--
316	TraesCS2D02G270000.2#domain1#PF00010	95.7%	32.7%	--
317	TraesCS2B02G289700.2#domain1#PF00010	95.7%	32.7%	--
318	TraesCS5A02G533900.1#domain1#PF00010	91.5%	30.4%	--
319	TraesCSU02G138400.2#domain1#PF00010	91.5%	30.4%	--
320	TraesCS3B02G018400.1#domain1#PF00010	95.7%	28.8%	--
321	TraesCS3A02G028200.3#domain1#PF00010	95.7%	28.8%	--

```
322 TraesCS3D02G017700.1#domain1#PF00010       95.7%  28.8%     --------------------------------------------------------------------------------
323 TraesCS2B02G237300.1#domain1#PF00010       95.7%  40.8%     --------------------------------------------------------------------------------
324 TraesCS2A02G212200.1#domain1#PF00010       95.7%  38.8%     --------------------------------------------------------------------------------
325 TraesCS2D02G218100.1#domain1#PF00010       95.7%  38.8%     --------------------------------------------------------------------------------
326 TraesCS4D02G224600.1#domain2#PF00010       95.7%   4.9%     GSASVSLWPWYKQMEQIDSSLGLLTKKFINLLKQAEDGILDLNNAAETLEVQKRRIYDITNVLEGIGLIEKTLKNRIRWK
327 TraesCS2D02G575600.3#domain2#PF00010       95.7%  20.4%     --------------------------------------------------------------------------------
328 TraesCSU02G029400.1#domain2#PF00010        95.7%  22.4%     --------------------------------------------------------------------------------
329 TraesCS4B02G397400.1#domain2#PF00010       95.7%  22.4%     --------------------------------------------------------------------------------
330 TraesCS5A02G558500.1#domain2#PF00010       95.7%  20.4%     --------------------------------------------------------------------------------
331 TraesCS5A02G489500.1#domain2#PF00010       95.7%  20.4%     --------------------------------------------------------------------------------
332 TraesCS4B02G397300.1#domain1#PF00010       95.7%  18.4%     --------------------------------------------------------------------------------
333 TraesCS2D02G406900.1#domain2#PF00010       95.7%  22.0%     --------------------------------------------------------------------------------
334 TraesCS2B02G428000.1#domain2#PF00010       95.7%  22.0%     --------------------------------------------------------------------------------
335 TraesCS2A02G409600.2#domain2#PF00010       95.7%  22.0%     --------------------------------------------------------------------------------
336 TraesCSU02G228400.1#domain1#PF00010        95.7%  22.0%     --------------------------------------------------------------------------------
337 TraesCSU02G237100.1#domain1#PF00010        95.7%  22.0%     --------------------------------------------------------------------------------
338 TraesCS2A02G409400.1#domain2#PF00010       95.7%  22.0%     --------------------------------------------------------------------------------
339 TraesCSU02G116000.1#domain2#PF00010        95.7%  26.5%     --------------------------------------------------------------------------------
340 TraesCS4B02G397800.1#domain2#PF00010       95.7%  26.5%     --------------------------------------------------------------------------------
341 TraesCS1B02G112900.1#domain2#PF00010       97.9%  20.0%     --------------------------------------------------------------------------------
342 TraesCS1B02G113100.4#domain2#PF00010       95.7%  27.1%     --------------------------------------------------------------------------------
343 TraesCS1A02G102400.2#domain2#PF00010       95.7%  27.1%     --------------------------------------------------------------------------------
344 TraesCS6B02G095600.1#domain1#PF00010       91.5%  39.1%     --------------------------------------------------------------------------------
345 TraesCS6D02G069300.1#domain1#PF00010       91.5%  41.3%     --------------------------------------------------------------------------------
346 TraesCS6A02G071200.1#domain1#PF00010       91.5%  41.3%     --------------------------------------------------------------------------------
347 TraesCS6B02G095700.1#domain1#PF00010       91.5%  39.1%     --------------------------------------------------------------------------------
348 TraesCS6D02G069400.1#domain1#PF00010       91.5%  39.1%     --------------------------------------------------------------------------------
349 TraesCS6A02G071300.1#domain1#PF00010       91.5%  39.1%     --------------------------------------------------------------------------------
350 TraesCS4B02G051500.2#domain1#PF00010       89.4%  33.3%     --------------------------------------------------------------------------------
351 TraesCS4D02G051600.2#domain1#PF00010       89.4%  33.3%     --------------------------------------------------------------------------------
352 TraesCS4A02G263300.2#domain1#PF00010       89.4%  33.3%     --------------------------------------------------------------------------------
353 TraesCS3B02G476000.1#domain1#PF00010       89.4%  31.1%     --------------------------------------------------------------------------------
354 TraesCS3D02G434900.1#domain1#PF00010       89.4%  31.1%     --------------------------------------------------------------------------------
355 TraesCS3A02G442200.1#domain1#PF00010       89.4%  31.1%     --------------------------------------------------------------------------------
356 TraesCS7A02G311800.1#domain1#PF00010       89.4%  33.3%     --------------------------------------------------------------------------------
357 TraesCS7B02G211600.1#domain1#PF00010       89.4%  33.3%     --------------------------------------------------------------------------------
358 TraesCS7D02G308300.1#domain1#PF00010       89.4%  33.3%     --------------------------------------------------------------------------------
359 TraesCS3A02G057900.1#domain1#PF00010       89.4%  31.1%     --------------------------------------------------------------------------------
360 TraesCS3D02G058100.1#domain1#PF00010       89.4%  28.9%     --------------------------------------------------------------------------------
361 TraesCS7A02G543300.1#domain1#PF00010       89.4%  31.1%     --------------------------------------------------------------------------------
362 TraesCS7D02G529200.1#domain1#PF00010       89.4%  31.1%     --------------------------------------------------------------------------------
363 TraesCS7B02G465400.1#domain1#PF00010       89.4%  31.1%     --------------------------------------------------------------------------------
364 TraesCSU02G069400.1#domain1#PF00010        91.5%  28.3%     --------------------------------------------------------------------------------
365 TraesCSU02G069300.1#domain1#PF00010        91.5%  28.3%     --------------------------------------------------------------------------------
366 TraesCS4D02G328800.1#domain1#PF00010       89.4%  26.7%     --------------------------------------------------------------------------------
367 TraesCS4B02G331900.1#domain1#PF00010       89.4%  28.9%     --------------------------------------------------------------------------------
368 TraesCS4B02G332000.1#domain1#PF00010       93.6%  26.0%     --------------------------------------------------------------------------------
369 TraesCS5A02G503400.1#domain1#PF00010       89.4%  28.9%     --------------------------------------------------------------------------------
370 TraesCS4D02G328900.1#domain1#PF00010       89.4%  28.9%     --------------------------------------------------------------------------------
371 TraesCS4D02G329100.1#domain1#PF00010       89.4%  28.9%     --------------------------------------------------------------------------------
372 TraesCS5A02G503300.1#domain1#PF00010       89.4%  28.9%     --------------------------------------------------------------------------------
373 TraesCS5A02G503500.1#domain1#PF00010       89.4%  28.9%     --------------------------------------------------------------------------------
374 TraesCS4D02G329000.2#domain1#PF00010       89.4%  28.9%     --------------------------------------------------------------------------------
    consensus/100%                                              ................................................................................
    consensus/90%                                               ................................................................................
    consensus/80%                                               ................................................................................
    consensus/70%                                               ................................................................................

                                              cov    pid 241 ] 241
  1 TraesCS6B02G244800.1#domain1#PF00010      100.0% 100.0%     -
  2 TraesCS6A02G214900.1#domain1#PF00010      100.0% 100.0%     -
  3 TraesCS6D02G197500.1#domain1#PF00010      100.0%  97.9%     -
  4 TraesCS3D02G388700.1#domain1#PF00010      100.0%  83.0%     -
  5 TraesCS3B02G426900.1#domain1#PF00010      100.0%  83.0%     -
  6 TraesCS3A02G395000.1#domain1#PF00010      100.0%  83.0%     -
  7 TraesCS7A02G245800.1#domain1#PF00010      100.0%  78.7%     -
  8 TraesCS7D02G244300.1#domain1#PF00010      100.0%  78.7%     -
  9 TraesCS7B02G145800.1#domain1#PF00010      100.0%  78.7%     -
 10 TraesCS5A02G264800.1#domain1#PF00010      100.0%  76.6%     -
 11 TraesCS5D02G272800.1#domain1#PF00010      100.0%  76.6%     -
 12 TraesCS5B02G264300.1#domain1#PF00010      100.0%  76.6%     -
 13 TraesCS4B02G317900.1#domain1#PF00010       97.9%  37.5%     -
 14 TraesCS3B02G010400.1#domain1#PF00010       93.6%  44.4%     -
 15 TraesCS3B02G007700.1#domain1#PF00010       93.6%  44.4%     -
 16 TraesCS3D02G004300.1#domain1#PF00010       93.6%  44.4%     -
 17 TraesCS3A02G006300.1#domain1#PF00010       87.2%  47.6%     -
 18 TraesCS6A02G276200.1#domain1#PF00010       87.2%  45.2%     -
 19 TraesCS6B02G303600.1#domain1#PF00010       89.4%  46.5%     -
 20 TraesCS6D02G256600.1#domain1#PF00010       89.4%  46.5%     -
 21 TraesCS6D02G256400.1#domain1#PF00010       89.4%  44.2%     -
 22 TraesCS6D02G256500.1#domain1#PF00010       87.2%  45.2%     -
 23 TraesCS6A02G276100.1#domain1#PF00010       87.2%  45.2%     -
 24 TraesCS6B02G303500.1#domain1#PF00010       87.2%  45.2%     -
 25 TraesCS6D02G256300.1#domain1#PF00010       87.2%  45.2%     -
 26 TraesCS4B02G257200.1#domain1#PF00010       87.2%  45.2%     -
 27 TraesCS4A02G047700.1#domain1#PF00010       87.2%  45.2%     -
 28 TraesCS4D02G257100.1#domain1#PF00010       87.2%  45.2%     -
 29 TraesCS5A02G067600.1#domain1#PF00010       87.2%  50.0%     -
 30 TraesCS5D02G078800.1#domain1#PF00010       87.2%  50.0%     -
 31 TraesCS5B02G074500.1#domain1#PF00010       87.2%  52.4%     -
 32 TraesCS2A02G194200.1#domain1#PF00010       87.2%  50.0%     -
 33 TraesCS2B02G212700.1#domain1#PF00010       87.2%  50.0%     -
 34 TraesCS2D02G193700.1#domain1#PF00010       87.2%  50.0%     -
 35 TraesCS4A02G234500.1#domain1#PF00010       87.2%  50.0%     -
 36 TraesCS4D02G079500.1#domain1#PF00010       87.2%  50.0%     -
 37 TraesCS4B02G080700.1#domain1#PF00010       87.2%  50.0%     -
 38 TraesCS5B02G406100.1#domain1#PF00010       87.2%  52.4%     -
 39 TraesCS5D02G411600.1#domain1#PF00010       87.2%  50.0%     -
 40 TraesCS5A02G401300.1#domain1#PF00010       87.2%  50.0%     -
```

41	TraesCS1B02G359000.1#domain1#PF00010	85.1%	43.9%	-
42	TraesCS1A02G345200.1#domain1#PF00010	85.1%	43.9%	-
43	TraesCS1D02G347900.1#domain1#PF00010	85.1%	43.9%	-
44	TraesCS3A02G440600.1#domain1#PF00010	89.4%	48.8%	-
45	TraesCS3D02G433200.1#domain1#PF00010	89.4%	48.8%	-
46	TraesCS3B02G474700.1#domain1#PF00010	87.2%	50.0%	-
47	TraesCS3A02G350600.1.cds1#domain1#PF00010	85.1%	48.8%	-
48	TraesCS3B02G383000.1.cds1#domain1#PF00010	85.1%	48.8%	-
49	TraesCS3D02G344600.1.cds1#domain1#PF00010	85.1%	48.8%	-
50	TraesCS7B02G160500.1.cds1#domain1#PF00010	87.2%	21.7%	-
51	TraesCS7A02G340300.1#domain1#PF00010	100.0%	32.0%	-
52	TraesCS7B02G251900.1#domain1#PF00010	100.0%	32.0%	-
53	TraesCS7D02G347900.1#domain1#PF00010	100.0%	32.0%	-
54	TraesCS1B02G226200.2#domain1#PF00010	97.9%	46.8%	-
55	TraesCS1D02G215600.2#domain1#PF00010	97.9%	46.8%	-
56	TraesCS1A02G212700.1#domain1#PF00010	97.9%	46.8%	-
57	TraesCS2D02G461900.1#domain1#PF00010	97.9%	42.6%	-
58	TraesCS2D02G461700.3#domain1#PF00010	97.9%	42.9%	-
59	TraesCS2A02G461700.2#domain1#PF00010	97.9%	42.9%	-
60	TraesCS2B02G483300.1#domain1#PF00010	97.9%	42.9%	-
61	TraesCS1B02G100400.2#domain1#PF00010	97.9%	43.8%	-
62	TraesCS1A02G083000.1#domain1#PF00010	97.9%	43.8%	-
63	TraesCS1D02G084200.1#domain1#PF00010	97.9%	43.8%	-
64	TraesCS5B02G054800.2#domain1#PF00010	97.9%	46.9%	-
65	TraesCS5A02G049600.1#domain1#PF00010	97.9%	46.9%	-
66	TraesCS5D02G060300.1#domain1#PF00010	97.9%	46.9%	-
67	TraesCS5A02G376500.1#domain1#PF00010	97.9%	45.8%	-
68	TraesCS5B02G380200.1#domain1#PF00010	97.9%	45.8%	-
69	TraesCS5D02G386500.1#domain1#PF00010	97.9%	45.8%	-
70	TraesCS2A02G253900.2#domain1#PF00010	97.9%	41.7%	-
71	TraesCS2B02G273500.1#domain1#PF00010	97.9%	41.7%	-
72	TraesCS2D02G254400.3#domain1#PF00010	97.9%	41.7%	-
73	TraesCS5A02G420200.1#domain1#PF00010	97.9%	41.7%	-
74	TraesCS5B02G422000.1#domain1#PF00010	97.9%	41.7%	-
75	TraesCS5D02G428400.2#domain1#PF00010	97.9%	41.7%	-
76	TraesCS6B02G411300.1#domain1#PF00010	97.9%	41.7%	-
77	TraesCS6A02G373500.2#domain1#PF00010	97.9%	43.8%	-
78	TraesCS6D02G357700.1#domain1#PF00010	97.9%	43.8%	-
79	TraesCS7A02G126900.1#domain1#PF00010	97.9%	43.8%	-
80	TraesCS7D02G124700.1#domain1#PF00010	97.9%	43.8%	-
81	TraesCS7B02G026300.1#domain1#PF00010	97.9%	43.8%	-
82	TraesCS6A02G190600.1#domain1#PF00010	95.7%	42.9%	-
83	TraesCS7A02G323000.1.cds1#domain1#PF00010	85.1%	51.2%	-
84	TraesCS7D02G319600.1.cds1#domain1#PF00010	85.1%	51.2%	-
85	TraesCS7B02G223900.1.cds1#domain1#PF00010	85.1%	51.2%	-
86	TraesCS7D02G263400.1.cds1#domain1#PF00010	85.1%	48.8%	-
87	TraesCS7A02G262600.1.cds1#domain1#PF00010	85.1%	48.8%	-
88	TraesCS7B02G303300.1#domain1#PF00010	100.0%	35.4%	-
89	TraesCS7D02G397300.1#domain1#PF00010	100.0%	35.4%	-
90	TraesCS7A02G157700.1#domain1#PF00010	97.9%	51.1%	-
91	TraesCS7D02G158300.1#domain1#PF00010	97.9%	51.1%	-
92	TraesCS7B02G062100.2#domain1#PF00010	97.9%	51.1%	-
93	TraesCS7A02G147500.1#domain1#PF00010	97.9%	48.9%	-
94	TraesCS7D02G149400.1#domain1#PF00010	97.9%	48.9%	-
95	TraesCS6B02G391100.1#domain1#PF00010	97.9%	46.8%	-
96	TraesCS6D02G341200.1#domain1#PF00010	97.9%	46.8%	-
97	TraesCS6A02G358400.3#domain1#PF00010	97.9%	48.9%	-
98	TraesCS5A02G225700.1#domain1#PF00010	97.9%	48.9%	-
99	TraesCS5D02G233000.1#domain1#PF00010	100.0%	46.9%	-
100	TraesCS5B02G224200.1#domain1#PF00010	100.0%	46.9%	-
101	TraesCS5D02G300100.1#domain1#PF00010	97.9%	40.8%	-
102	TraesCS5B02G292100.1#domain1#PF00010	97.9%	40.8%	-
103	TraesCS5D02G459800.1#domain1#PF00010	97.9%	32.7%	-
104	TraesCS5B02G457600.1#domain1#PF00010	97.9%	32.7%	-
105	TraesCS4D02G237100.3#domain1#PF00010	100.0%	40.0%	-
106	TraesCS4B02G235700.1#domain1#PF00010	97.9%	38.0%	-
107	TraesCS4A02G060200.2#domain1#PF00010	97.9%	38.0%	-
108	TraesCS4A02G255800.3#domain1#PF00010	97.9%	42.0%	-
109	TraesCS4D02G058900.1#domain1#PF00010	97.9%	40.0%	-
110	TraesCS4B02G059000.1#domain1#PF00010	97.9%	40.0%	-
111	TraesCS7D02G327900.1#domain1#PF00010	95.7%	24.5%	-
112	TraesCS7A02G350900.1#domain1#PF00010	95.7%	24.5%	-
113	TraesCS7B02G232000.1#domain1#PF00010	95.7%	24.5%	-
114	TraesCS4B02G347100.1#domain1#PF00010	95.7%	22.2%	-
115	TraesCS5A02G515800.1#domain1#PF00010	95.7%	22.2%	-
116	TraesCS4D02G342000.1#domain1#PF00010	95.7%	22.2%	-
117	TraesCS2B02G141600.1#domain1#PF00010	93.6%	24.5%	-
118	TraesCS2D02G122800.1#domain1#PF00010	93.6%	26.0%	-
119	TraesCS2A02G120700.1#domain1#PF00010	93.6%	26.0%	-
120	TraesCS6B02G346100.1#domain1#PF00010	95.7%	20.4%	-
121	TraesCS6D02G294900.1#domain1#PF00010	95.7%	20.4%	-
122	TraesCS6A02G315700.1#domain1#PF00010	95.7%	20.4%	-
123	TraesCS1B02G300100.1#domain1#PF00010	95.7%	25.9%	-
124	TraesCS1D02G289400.1#domain1#PF00010	95.7%	25.9%	-
125	TraesCS1A02G290800.1#domain1#PF00010	95.7%	25.9%	-
126	TraesCS6D02G315200.1#domain1#PF00010	95.7%	20.4%	-
127	TraesCS6A02G335500.1#domain1#PF00010	95.7%	20.4%	-
128	TraesCS6B02G366200.1#domain1#PF00010	95.7%	20.4%	-
129	TraesCS6A02G264200.1#domain1#PF00010	95.7%	20.4%	-
130	TraesCS6B02G291200.1#domain1#PF00010	95.7%	20.4%	-
131	TraesCS6D02G250700.1#domain1#PF00010	95.7%	20.4%	-
132	TraesCS2B02G469700.1#domain1#PF00010	95.7%	20.4%	-
133	TraesCS2B02G469800.1#domain1#PF00010	95.7%	20.4%	-
134	TraesCS2D02G448000.1#domain1#PF00010	95.7%	20.4%	-
135	TraesCS2A02G448800.1#domain1#PF00010	95.7%	20.4%	-
136	TraesCS4B02G304500.1#domain1#PF00010	95.7%	20.4%	-
137	TraesCS4A02G404700.1#domain1#PF00010	95.7%	20.4%	-
138	TraesCS4D02G302700.1#domain1#PF00010	95.7%	20.4%	-
139	TraesCS5B02G245900.1#domain1#PF00010	95.7%	20.4%	-

140	TraesCS5A02G248200.1#domain1#PF00010	95.7%	20.4%	-
141	TraesCS5D02G255100.1#domain1#PF00010	95.7%	20.4%	-
142	TraesCS4B02G391500.1#domain1#PF00010	95.7%	24.0%	-
143	TraesCS5A02G555200.1#domain1#PF00010	95.7%	24.0%	-
144	TraesCS4B02G345800.1#domain1#PF00010	95.7%	24.0%	-
145	TraesCS5A02G505100.1#domain1#PF00010	95.7%	26.0%	-
146	TraesCS4B02G234700.1#domain1#PF00010	95.7%	26.0%	-
147	TraesCS4D02G235900.1#domain1#PF00010	95.7%	26.0%	-
148	TraesCS4B02G335200.1#domain1#PF00010	95.7%	26.0%	-
149	TraesCS4D02G330500.1#domain1#PF00010	95.7%	26.0%	-
150	TraesCS4B02G056600.2#domain1#PF00010	95.7%	28.0%	-
151	TraesCS4D02G056900.2#domain1#PF00010	95.7%	28.0%	-
152	TraesCS4A02G257900.2#domain1#PF00010	95.7%	28.0%	-
153	TraesCS4B02G021100.1#domain1#PF00010	95.7%	28.6%	-
154	TraesCS4B02G021500.1#domain1#PF00010	95.7%	28.6%	-
155	TraesCS4D02G019200.1#domain1#PF00010	95.7%	28.6%	-
156	TraesCS4A02G292800.1#domain1#PF00010	95.7%	28.6%	-
157	TraesCS4B02G020900.1#domain1#PF00010	95.7%	28.6%	-
158	TraesCS4B02G021200.1#domain1#PF00010	95.7%	26.5%	-
159	TraesCS4D02G018700.1#domain1#PF00010	95.7%	26.5%	-
160	TraesCS3A02G019100.1#domain1#PF00010	95.7%	27.1%	-
161	TraesCS3A02G019000.1#domain1#PF00010	95.7%	27.1%	-
162	TraesCS3B02G029800.1#domain1#PF00010	95.7%	24.5%	-
163	TraesCS3D02G014900.1#domain1#PF00010	95.7%	24.5%	-
164	TraesCS4A02G408700.1#domain1#PF00010	95.7%	28.6%	-
165	TraesCS4D02G306400.1#domain1#PF00010	95.7%	26.5%	-
166	TraesCS4B02G308300.1#domain1#PF00010	95.7%	26.5%	-
167	TraesCS4B02G308200.1#domain1#PF00010	95.7%	26.5%	-
168	TraesCS4A02G408800.1#domain1#PF00010	95.7%	26.5%	-
169	TraesCS4B02G308100.1#domain1#PF00010	93.6%	28.6%	-
170	TraesCS4D02G306300.1#domain1#PF00010	93.6%	28.6%	-
171	TraesCS7B02G483600.1#domain1#PF00010	95.7%	33.3%	-
172	TraesCS7A02G558700.1#domain1#PF00010	95.7%	33.3%	-
173	TraesCS5B02G518800.1#domain1#PF00010	95.7%	22.4%	-
174	TraesCSU02G075100.1#domain1#PF00010	91.5%	22.9%	-
175	TraesCS5B02G013000.2#domain1#PF00010	95.7%	30.6%	-
176	TraesCS5D02G020600.1#domain1#PF00010	95.7%	30.6%	-
177	TraesCS5A02G014800.1#domain1#PF00010	95.7%	30.6%	-
178	TraesCS4B02G020700.1#domain1#PF00010	95.7%	24.5%	-
179	TraesCS4D02G018800.2#domain1#PF00010	95.7%	26.5%	-
180	TraesCS4D02G019100.1#domain1#PF00010	95.7%	26.5%	-
181	TraesCS4A02G292700.1#domain1#PF00010	95.7%	26.5%	-
182	TraesCSU02G075200.3#domain1#PF00010	95.7%	24.5%	-
183	TraesCS5B02G518500.1#domain1#PF00010	95.7%	24.5%	-
184	TraesCS5B02G518400.1#domain1#PF00010	95.7%	26.0%	-
185	TraesCS4A02G354100.1#domain1#PF00010	95.7%	26.0%	-
186	TraesCS5D02G517900.1#domain1#PF00010	95.7%	26.0%	-
187	TraesCS7A02G421000.1#domain1#PF00010	95.7%	25.5%	-
188	TraesCS7B02G321400.1#domain1#PF00010	95.7%	25.5%	-
189	TraesCS3A02G489400.1#domain1#PF00010	95.7%	19.6%	-
190	TraesCS3B02G549700.1#domain1#PF00010	95.7%	19.6%	-
191	TraesCS3D02G495300.1#domain1#PF00010	95.7%	19.6%	-
192	TraesCS3A02G489500.1#domain1#PF00010	95.7%	21.8%	-
193	TraesCS3D02G495400.1#domain1#PF00010	95.7%	21.8%	-
194	TraesCS3B02G549800.1#domain1#PF00010	95.7%	21.8%	-
195	TraesCS2B02G543700.1#domain1#PF00010	95.7%	23.6%	-
196	TraesCS2B02G543800.1#domain1#PF00010	95.7%	23.6%	-
197	TraesCS2D02G517000.1#domain1#PF00010	95.7%	23.6%	-
198	TraesCS2A02G515300.1#domain1#PF00010	95.7%	23.6%	-
199	TraesCS3B02G550200.2#domain1#PF00010	95.7%	21.8%	-
200	TraesCS3D02G495700.1#domain1#PF00010	95.7%	21.8%	-
201	TraesCS3A02G489700.1#domain1#PF00010	95.7%	21.8%	-
202	TraesCS3B02G550000.1#domain1#PF00010	95.7%	21.8%	-
203	TraesCS3D02G495600.1#domain1#PF00010	95.7%	21.8%	-
204	TraesCS3A02G489600.1#domain1#PF00010	93.6%	18.5%	-
205	TraesCS6A02G114100.1#domain1#PF00010	95.7%	32.7%	-
206	TraesCS6B02G142000.1#domain1#PF00010	95.7%	32.7%	-
207	TraesCS2A02G442500.1#domain1#PF00010	87.2%	24.4%	-
208	TraesCS6D02G246100.2#domain1#PF00010	91.5%	26.0%	-
209	TraesCS6B02G295900.2#domain1#PF00010	91.5%	26.0%	-
210	TraesCS6A02G268800.1#domain1#PF00010	91.5%	26.0%	-
211	TraesCS2D02G441800.1#domain1#PF00010	91.5%	28.6%	-
212	TraesCS2A02G442700.1#domain1#PF00010	91.5%	28.6%	-
213	TraesCS2B02G463800.1#domain1#PF00010	91.5%	28.6%	-
214	TraesCS6D02G185100.1#domain1#PF00010	91.5%	18.9%	-
215	TraesCS6B02G220300.1#domain1#PF00010	91.5%	18.9%	-
216	TraesCS6A02G204300.1#domain1#PF00010	91.5%	18.9%	-
217	TraesCS1D02G280600.1#domain1#PF00010	93.6%	18.0%	-
218	TraesCS1B02G290500.1#domain1#PF00010	93.6%	18.0%	-
219	TraesCS1A02G281300.1#domain1#PF00010	93.6%	18.0%	-
220	TraesCS6A02G045100.1#domain1#PF00010	93.6%	14.1%	-
221	TraesCS6D02G051600.2#domain1#PF00010	93.6%	14.1%	-
222	TraesCS6B02G060700.2#domain1#PF00010	93.6%	14.1%	-
223	TraesCS7A02G307700.1#domain1#PF00010	93.6%	17.6%	-
224	TraesCS7B02G208000.1#domain1#PF00010	93.6%	17.6%	-
225	TraesCS7D02G304500.1#domain1#PF00010	93.6%	17.6%	-
226	TraesCS2B02G240600.1#domain1#PF00010	93.6%	16.0%	-
227	TraesCS2A02G215600.1#domain1#PF00010	93.6%	16.0%	-
228	TraesCS2D02G221200.1#domain1#PF00010	93.6%	16.0%	-
229	TraesCS7D02G360600.1#domain1#PF00010	91.5%	26.5%	-
230	TraesCS7B02G265900.1#domain1#PF00010	91.5%	26.5%	-
231	TraesCS7A02G362500.1#domain1#PF00010	91.5%	26.5%	-
232	TraesCS6A02G186200.1#domain1#PF00010	87.2%	21.3%	-
233	TraesCS6D02G173300.1#domain1#PF00010	87.2%	21.3%	-
234	TraesCS6B02G215200.1#domain1#PF00010	87.2%	21.3%	-
235	TraesCS5A02G251390.4#domain1#PF00010	95.7%	30.8%	-
236	TraesCS6A02G288100.3#domain1#PF00010	95.7%	36.5%	-
237	TraesCS6B02G317600.2#domain1#PF00010	95.7%	36.5%	-
238	TraesCS6D02G270900.1#domain1#PF00010	95.7%	36.5%	-

239	TraesCS5A02G466300.1#domain1#PF00010	91.5%	26.0%	-
240	TraesCS5B02G478000.1#domain1#PF00010	91.5%	26.0%	-
241	TraesCS5D02G479100.1#domain1#PF00010	91.5%	26.0%	-
242	TraesCS1B02G129200.1#domain1#PF00010	91.5%	29.2%	-
243	TraesCS1D02G112000.1#domain1#PF00010	91.5%	29.2%	-
244	TraesCS1A02G110400.1#domain1#PF00010	91.5%	29.2%	-
245	TraesCS3A02G102900.1#domain1#PF00010	91.5%	28.6%	-
246	TraesCS3D02G105100.2#domain1#PF00010	91.5%	29.2%	-
247	TraesCS3B02G120200.1#domain1#PF00010	93.6%	30.6%	-
248	TraesCS3B02G120500.1#domain1#PF00010	93.6%	30.6%	-
249	TraesCS3A02G102600.1#domain1#PF00010	91.5%	29.2%	-
250	TraesCS2B02G494900.1#domain1#PF00010	93.6%	35.3%	-
251	TraesCS2A02G472300.1#domain1#PF00010	93.6%	35.3%	-
252	TraesCS2D02G472000.1#domain1#PF00010	93.6%	35.3%	-
253	TraesCS5A02G245600.1#domain1#PF00010	91.5%	32.0%	-
254	TraesCS5D02G252300.1#domain1#PF00010	91.5%	32.0%	-
255	TraesCS5B02G243000.1#domain1#PF00010	91.5%	32.0%	-
256	TraesCS5B02G229000.1#domain1#PF00010	91.5%	38.0%	-
257	TraesCS5A02G230500.1#domain1#PF00010	91.5%	38.0%	-
258	TraesCS5D02G237300.1#domain1#PF00010	91.5%	38.0%	-
259	TraesCS3A02G142900.1#domain1#PF00010	91.5%	32.7%	-
260	TraesCS3D02G144700.1#domain1#PF00010	91.5%	30.6%	-
261	TraesCS3A02G252900.1.cds1#domain2#PF00010	95.7%	24.5%	-
262	TraesCS3B02G284800.1.cds1#domain2#PF00010	95.7%	24.5%	-
263	TraesCS3D02G253700.1.cds1#domain2#PF00010	95.7%	24.5%	-
264	TraesCS3B02G288700.1#domain2#PF00010	95.7%	24.5%	-
265	5gnj:G	95.7%	17.3%	-
266	TraesCS1B02G208000.1.cds1#domain2#PF00010	93.6%	27.1%	-
267	TraesCS1D02G196900.1.cds1#domain2#PF00010	93.6%	27.1%	-
268	TraesCS1A02G193200.1#domain2#PF00010	93.6%	27.1%	-
269	TraesCS3B02G185400.1#domain2#PF00010	95.7%	28.6%	-
270	TraesCS3D02G166300.2#domain2#PF00010	95.7%	28.6%	-
271	TraesCS3A02G158600.1.cds1#domain2#PF00010	95.7%	28.6%	-
272	TraesCS4A02G028900.1.cds1#domain2#PF00010	95.7%	30.6%	-
273	TraesCS4D02G275500.1.cds1#domain2#PF00010	95.7%	30.6%	-
274	TraesCS4B02G276900.1.cds1#domain2#PF00010	95.7%	30.6%	-
275	TraesCS2A02G448500.1.cds1#domain1#PF00010	93.6%	28.6%	-
276	TraesCS2B02G469200.1#domain1#PF00010	93.6%	28.6%	-
277	TraesCSU02G222600.1#domain1#PF00010	93.6%	28.6%	-
278	TraesCS2B02G469300.1.cds1#domain1#PF00010	93.6%	28.6%	-
279	TraesCS5A02G306600.1.cds1#domain1#PF00010	93.6%	27.1%	-
280	TraesCS5D02G313700.1.cds1#domain1#PF00010	93.6%	27.1%	-
281	TraesCS5B02G307100.1.cds1#domain1#PF00010	93.6%	29.2%	-
282	TraesCS5A02G306700.1.cds1#domain1#PF00010	93.6%	26.5%	-
283	TraesCS7D02G102200.1.cds1#domain1#PF00010	93.6%	24.5%	-
284	TraesCS1A02G369200.1.cds1#domain1#PF00010	93.6%	27.7%	-
285	TraesCS1D02G374800.1.cds1#domain1#PF00010	93.6%	27.7%	-
286	TraesCS5A02G306400.1.cds1#domain1#PF00010	93.6%	24.5%	-
287	TraesCS5B02G307000.1.cds1#domain1#PF00010	93.6%	24.5%	-
288	TraesCS5D02G313600.1.cds1#domain1#PF00010	93.6%	24.5%	-
289	TraesCS5A02G306200.1.cds1#domain1#PF00010	93.6%	24.5%	-
290	TraesCS5D02G313500.1.cds1#domain1#PF00010	93.6%	24.5%	-
291	TraesCS5B02G306800.1.cds1#domain1#PF00010	93.6%	24.5%	-
292	TraesCS5A02G306500.1.cds1#domain1#PF00010	93.6%	22.4%	-
293	TraesCS5B02G306600.1#domain1#PF00010	93.6%	25.0%	-
294	TraesCS5D02G313400.1.cds1#domain1#PF00010	93.6%	25.0%	-
295	TraesCS3D02G106600.2#domain1#PF00010	93.6%	25.0%	-
296	TraesCS3A02G104400.1#domain1#PF00010	93.6%	25.0%	-
297	TraesCS3B02G122800.1#domain1#PF00010	93.6%	25.0%	-
298	TraesCS3B02G122900.1#domain1#PF00010	95.7%	26.5%	-
299	TraesCS3B02G236900.1#domain1#PF00010	95.7%	25.0%	-
300	TraesCS3A02G204800.1#domain1#PF00010	95.7%	25.0%	-
301	TraesCS3D02G210600.1#domain1#PF00010	95.7%	25.0%	-
302	TraesCS4A02G087000.1#domain1#PF00010	93.6%	36.7%	-
303	TraesCS4B02G217400.1#domain1#PF00010	93.6%	36.7%	-
304	TraesCS4D02G217700.1#domain1#PF00010	93.6%	36.7%	-
305	TraesCS2A02G281200.1#domain1#PF00010	93.6%	36.2%	-
306	TraesCS2D02G280100.1#domain1#PF00010	93.6%	36.2%	-
307	TraesCS2B02G298600.1#domain1#PF00010	93.6%	36.2%	-
308	TraesCS3A02G442600.1#domain1#PF00010	85.1%	34.9%	-
309	TraesCS3D02G435200.1#domain1#PF00010	85.1%	32.6%	-
310	TraesCS2B02G289900.1#domain1#PF00010	95.7%	31.2%	-
311	TraesCS2D02G270300.1#domain1#PF00010	95.7%	31.2%	-
312	TraesCS2A02G271700.1#domain1#PF00010	95.7%	29.2%	-
313	TraesCS4B02G364800.1#domain1#PF00010	95.7%	30.6%	-
314	TraesCS5A02G533200.1#domain1#PF00010	95.7%	30.6%	-
315	TraesCSU02G138900.1#domain1#PF00010	95.7%	30.6%	-
316	TraesCS2D02G270000.2#domain1#PF00010	95.7%	32.7%	-
317	TraesCS2B02G289700.2#domain1#PF00010	95.7%	32.7%	-
318	TraesCS5A02G533900.1#domain1#PF00010	91.5%	30.4%	-
319	TraesCSU02G138400.2#domain1#PF00010	91.5%	30.4%	-
320	TraesCS3B02G018400.1#domain1#PF00010	95.7%	28.8%	-
321	TraesCS3A02G028200.3#domain1#PF00010	95.7%	28.8%	-
322	TraesCS3D02G017700.1#domain1#PF00010	95.7%	28.8%	-
323	TraesCS2B02G237300.1#domain1#PF00010	95.7%	40.8%	-
324	TraesCS2A02G212200.1#domain1#PF00010	95.7%	38.8%	-
325	TraesCS2D02G218100.1#domain1#PF00010	95.7%	38.8%	-
326	TraesCS4D02G224600.1#domain2#PF00010	95.7%	4.9%	G
327	TraesCS2D02G575600.3#domain2#PF00010	95.7%	20.4%	-
328	TraesCSU02G029400.1#domain2#PF00010	95.7%	22.4%	-
329	TraesCS4B02G397400.1#domain2#PF00010	95.7%	22.4%	-
330	TraesCS5A02G558500.1#domain2#PF00010	95.7%	20.4%	-
331	TraesCS5A02G489500.1#domain2#PF00010	95.7%	20.4%	-
332	TraesCS4B02G397300.1#domain2#PF00010	95.7%	18.4%	-
333	TraesCS2D02G406900.1#domain2#PF00010	95.7%	22.0%	-
334	TraesCS2B02G428000.1#domain2#PF00010	95.7%	22.0%	-
335	TraesCS2A02G409600.2#domain2#PF00010	95.7%	22.0%	-
336	TraesCSU02G228400.1#domain1#PF00010	95.7%	22.0%	-
337	TraesCSU02G237100.1#domain1#PF00010	95.7%	22.0%	-

338	TraesCS2A02G409400.1#domain2#PF00010	95.7%	22.0%	-
339	TraesCSU02G116000.1#domain2#PF00010	95.7%	26.5%	-
340	TraesCS4B02G397800.1#domain2#PF00010	95.7%	26.5%	-
341	TraesCS1B02G112900.1#domain2#PF00010	97.9%	20.0%	-
342	TraesCS1B02G113100.4#domain2#PF00010	95.7%	27.1%	-
343	TraesCS1A02G102400.2#domain2#PF00010	95.7%	27.1%	-
344	TraesCS6B02G095600.1#domain1#PF00010	91.5%	39.1%	-
345	TraesCS6D02G069300.1#domain1#PF00010	91.5%	41.3%	-
346	TraesCS6A02G071200.1#domain1#PF00010	91.5%	41.3%	-
347	TraesCS6B02G095700.1#domain1#PF00010	91.5%	39.1%	-
348	TraesCS6D02G069400.1#domain1#PF00010	91.5%	39.1%	-
349	TraesCS6A02G071300.1#domain1#PF00010	91.5%	39.1%	-
350	TraesCS4B02G051500.2#domain1#PF00010	89.4%	33.3%	-
351	TraesCS4D02G051600.2#domain1#PF00010	89.4%	33.3%	-
352	TraesCS4A02G263300.2#domain1#PF00010	89.4%	33.3%	-
353	TraesCS3B02G476000.1#domain1#PF00010	89.4%	31.1%	-
354	TraesCS3D02G434900.1#domain1#PF00010	89.4%	31.1%	-
355	TraesCS3A02G442200.1#domain1#PF00010	89.4%	31.1%	-
356	TraesCS7A02G311800.1#domain1#PF00010	89.4%	33.3%	-
357	TraesCS7B02G211600.1#domain1#PF00010	89.4%	33.3%	-
358	TraesCS7D02G308300.1#domain1#PF00010	89.4%	33.3%	-
359	TraesCS3A02G057900.1#domain1#PF00010	89.4%	31.1%	-
360	TraesCS3D02G058100.1#domain1#PF00010	89.4%	28.9%	-
361	TraesCS7A02G543300.1#domain1#PF00010	89.4%	31.1%	-
362	TraesCS7D02G529200.1#domain1#PF00010	89.4%	31.1%	-
363	TraesCS7B02G465400.1#domain1#PF00010	89.4%	31.1%	-
364	TraesCSU02G069400.1#domain1#PF00010	91.5%	28.3%	-
365	TraesCSU02G069300.1#domain1#PF00010	91.5%	28.3%	-
366	TraesCS4D02G328800.1#domain1#PF00010	89.4%	26.7%	-
367	TraesCS4B02G331900.1#domain1#PF00010	89.4%	28.9%	-
368	TraesCS4B02G332000.1#domain1#PF00010	93.6%	26.0%	-
369	TraesCS5A02G503400.1#domain1#PF00010	89.4%	28.9%	-
370	TraesCS4D02G328900.1#domain1#PF00010	89.4%	28.9%	-
371	TraesCS4D02G329100.1#domain1#PF00010	89.4%	28.9%	-
372	TraesCS5A02G503300.1#domain1#PF00010	89.4%	28.9%	-
373	TraesCS5A02G503500.1#domain1#PF00010	89.4%	28.9%	-
374	TraesCS4D02G329000.2#domain1#PF00010	89.4%	28.9%	-
	consensus/100%			.
	consensus/90%			.
	consensus/80%			.
	consensus/70%			.

MView 1.67, Copyright © 1997-2020 Nigel P. Brown
