# Supplementary material for: Genome-wide identification and expression analysis of salt-responsive bHLH transcription factors in the wheat (Triticum aestivum) genome
Source: Front Plant Sci. 2026 Apr 23;17:1770759. doi: 10.3389/fpls.2026.1770759 (PMC13151688; doi:10.3389/fpls.2026.1770759)
Supplement: Supplementary Figure 3 — An HTML version of the Interactive co-expression network is provided as a ZIP, which can be downloaded and viewed locally in a web browser. [file DataSheet2.pdf]

```
                         10        20        30        40        50        60
                ....*....|....*....|....*....|....*....|....*....|....*....|
query         1 ~~~~~~~~~~~~~~~~~~~~~~~~~mdgdqaphsnsnqhpppppspsplpereasfnydi 35
gi_1742959      ~~~~~~~~~~~~~~~~~~~~~~~~~~~~~~~~~~~~~~~~~~~~~~~~~~~~~~~~~~~~
gi_15215300   1 ~~~~~~~~~~~~~~~~~~~~manvskkvswsgrdrddeeaapllrrtarpgggtpllnga 40
gi_15240576   1 ~~~~~~~~~~~~~~~~~~~~~~~~~~~~~~~~~~~~~~~~~~~~~~~~~~~mddrhegdh 9
gi_19922112   1 mddrkalitldgessaeddpiilqkeagptskavqdfglhqreepstsalarratlsgnn 60
gi_21321026   1 ~~~~~~~~~~~~~~~~~~~~~~~~~~~~~~~~~~megqsqhraperegshnydiestdgs 26
gi_48374433   1 ~~~~~~~~~~~~~~~~~~~~~~~~~~~~~~~~~~~~~~~~~~~~~~~~~~~~mdgsgagg 8
gi_66818603   1 ~~~~~~~~~~~~~~~~~~~~~~~~~~~~~~~~~~~~~~~~~~~~~~~msnnykagtaing 13
gi_71897153   1 ~~~~~~~~~~~~~~~~~~~~~~~~manvakkvswsgrdprddederagettpllngtgpg 36
                         70        80        90       100       110       120
                ....*....|....*....|....*....|....*....|....*....|....*....|
query        36 esmdgggwrgagryassdallrydddgprepllrkrtmnttsqiaivganvfaiESLDYE 95
gi_1742959    1 ~~~~~~~~~~mlsnhlqngiesdnllwsrvpesddtstdditllnshrdgdggvNSLDYE 50
gi_15215300  41 gpgaarqsprsalfrvghmssvelddelldpdmdpphpfpkeiphnekllslkyESLDYD 100
gi_15240576  10 hdieveggalhgferkisgilddgsvgfrqpllarnrknttsqiaivgantcpiESLDYE 69
gi_19922112  61 hllddsqpllmgnnsvppdipingsagspnrninssepifhlrsrtaaastpnyESLDYE 120
gi_21321026  27 gglwrrngsssgallryndsgggrsgsageplllrkrtmnttsqiaivganvcpiESLDYE 86
gi_48374433   9 pppwsahhngsseallryddggadhgstqqpllrkrtvnttsqiaivganvcpiESLDYE 68
gi_66818603  14 ndylllnidgnkdcdtdngffsgknffggnqtsithrrhhkmttktdrelmshfESLDFN 73
gi_71897153  37 saggarqftpssflrpgqlsnvdlnedireletelprpypneiphnekllslkyESLDYD 96
                        130       140       150       160       170       180
                ....*....|....*....|....*....|....*....|....*....|....*....|
query        96 IVENdlfkqdwrsrk~knqifqyvvlKWALVLLIGLLTGLVGFFNNLAVENIAGFKLVlt 154
gi_1742959   51 VIENyayreeqahrg~klyvgyyvavKWFFSLLIGIGTGLAAVFINLSVENFAGWKFAlt 109
gi_15215300 101 NSENqlfleeerrin~htafrtveikRWVICALIGILTGLVACFIDIVVENLAGLKYRvi 159
gi_15240576  70 IFENdffkqdwrsrk~kieilqytflKWALAFLIGLATGLVGFLNNLGVENIAGFKLLli 128
gi_19922112 121 VCENtlfqdeqrkrlterfslrkdiiRWIIFIQIGIITALIACTIDIIIEELSIRKYTfl 180
gi_21321026  87 VVENdlfkqdwrsrk~kkqifqyivlKWTLVLLIGLLTGLVGFFNNLAVENIAGFKLLlt 145
gi_48374433  69 VVENdlfkqdwrsrk~kkqifqyivlKWSLVLLIGLLTGFVGFFNNLAVENIAGFKLLlt 127
gi_66818603  74 AIDNiihrkytfekk~kyqkilktlgKWVICTLIGVVVGLVCYCLKESVDQLQSLKLTqv 132
gi_71897153  97 NSENqlfleeerrin~haafrtveikRWVICAMIGILTGLVACFIDIVVENLAGLKYRvv 155
                        190       200       210       220       230       240
                ....*....|....*....|....*....|....*....|....*....|....*....|
query       155 gdlm~~~~~~~~~lqkryfTAFLAYGGCNLVLGATAAALCayIAPAAAGSGIPEVKAYLN 205
gi_1742959  110 faii~~~~~~~~~~qksyfAGFIVYLLINLVLVFSSAYIItqFAPAAAGSGIPEIKGYLN 159
gi_15215300 160 kgnidk~~~~~ftekgglsFSLLLWATLNAAFVLVGSVIVafIEPVAAGSGIPQIKCFLN 214
gi_15240576 129 gnlm~~~~~~~~~lkekyfQAFFAFAGCNLILATAAASLCafIAPAAAGSGIPEVKAYLN 179
gi_19922112 181 ynsvkenvplsdasdrdllIPYLYWLLFSIVPVAFGAAMVtyIEPITAGSGIPQVKSYLN 240
gi_21321026 146 gnlml~~~~~~~kgkcrylTAFFAYGGCNLVLAAAAAAICayIAPAAAGSGIPEVKAYLN 198
gi_48374433 128 sdlm~~~~~~~~~lkgryiRAFFVYGGCNLVLAAAAAAICayIAPAAAGSGIPEVKAYLN 178
gi_66818603 133 kkfys~~~~~~~~testifIPFLVYLGFNLCYGLISGLLVciFGPMSSSSGLPEVKGYLN 184
gi_71897153 156 kdnidk~~~~~ftekgglsFSLLLWATLNASVVMVGSVIVafIEPVAAGSGIPQIKCYLN 210
                        250       260       270       280       290       300
                ....*....|....*....|....*....|....*....|....*....|....*....|
```

G x GIPE

Serine

GK x GP xx H — Glutamine (E)

```
query       206 GVdaYSILAPSTLFVKIFGSILGVSGGFvLGKEGPMVHTGACIANLLGQGGSRkyhltwn 265
gi_1742959  160 GIdiPGTLLFRTLIGKIFGSIGSVGGGLaLGKEGPLVHTGACIASLLGQGGSTkyhlnsr 219
gi_15215300 215 GVkiPHVVRLKTLVIKVSGVILSVVGGLaVGKEGPMIHSGSVIAAGISQGRSTslkrdfk 274
gi_15240576 180 GIdaYSILAPSTLFVKIFGSIFGVAAGFvVGKEGPMVHTGACIANLLGQGGSKkyrltwk 239
gi_19922112 241 GVkiPRIVRIKTLAVKAIGVITSVVGGLaGGKEGPMIHAGAVVAAGISQGKSTtflkdfr 300
gi_21321026 199 GVdaYSILAPSTLFVKIFGSILGVSAGFvLGKEGPMVHTGACIANLLGQGGSRkyrltcn 258
gi_48374433 179 GVdaYSILAPSTLFVKIFGSILGVSAGFvLGKEGPMVHTGACIANLLGQGGSRkyhltcn 238
gi_66818603 185 GIriSKAFNLKTVLGKLVSLIFSFSSGLvLGPEGPMFHIGAGIGSSMSQFKSKtlkfhlk 244
gi_71897153 211 GVkiPHVVRLKTLVIKVCGVILSVVGGLaVGKEGPMIHSGAVIAAGISQGRSTslkrdfk 270
```

Valine (V)

P xx G x LF

```
                          310       320       330       340       350       360
                    ....*....|....*....|....*....|....*....|....*....|....*....|
query       266 wlkyFKNDRDRRDLITCGAAAGVAAAFRAPVGGVLFALEEAASWWRSALLWRTFFTTAVV 325
gi_1742959  220 wpqlFKSDRDRRDLVTCGCAAGVAAAFRAPVGGVLFALEEVTSWWRSQLMWRVFFTSAIV 279
gi_15215300 275 ifeyFRRDTEKRDFVSAGAAAGVSAAFGAPVGGVLFSLEEGASFWNQFLTWRIFFASMIS 334
gi_15240576 240 wlrfFKNDRDRRDLITCGAAAGVAAAFRAPVGGVLFALEEAASWWRNALLWRTFFTTAVV 299
gi_19922112 301 ifkaFRDDHEKRDFVLGGGAAGVSAAFGAPIGGMLFSLEEAASFWNQNLIWRTLVASIIS 360
gi_21321026 259 wlryFKNDRDRRDLITCGSAAGVAAAFRAPVGGVLFALEEAASWWRSALLWRAFFTTAVV 318
gi_48374433 239 wlryFKNDRDRRDLITCGSAAGVAAAFRAPVGGVLFALEEAASWWRSALLWRTFFTTAVV 298
gi_66818603 245 sfwiFQNDSDKRDFISCGAAAGIAAAFGAPIGGVLFCLEEGSSFWSRQLTWRTFFSCLIA 304
gi_71897153 271 ifeyFRRDTEKRDFVSAGAAAGVSAAFGAPVGGVLFSLEEGASFWNQFLTWRIFFASMIS 330

                          370       380       390       400       410       420
                    ....*....|....*....|....*....|....*....|....*....|....*....|
query       326 AVVLRAliefcrkgkcglfgqgglimfdlssnvpsYGTQDLIAIIILGVIGGVFGGLFNF 385
gi_1742959  280 AVVVRTamgwcksgicghfggggfiiwdvsdgqddYYFKELLPMAVIGVIGGLLGALFNQ 339
gi_15215300 335 TFTLNFvlsiyhgnmwdlsspglinfgrfdsekmaYTIHEIPVFIAMGVVGGVLGAVFNA 394
gi_15240576 300 AVVLRSliefcrsgrcglfgkgglimfdvnsgpvlYSTPDLLAIVFLGVIGGVLGSLYNY 359
gi_19922112 361 VFTLNIvlsayhglndftft~~glfnlgkfdtplkFDYFELPIFMILGVTGGLLGAAWNS 418
gi_21321026 319 AVVLRSliefcrsgkcglfgqgglimfdlsstvatYSTPDLIAIIILGIIGGIFGGLFNF 378
gi_48374433 299 AVVLRGliefcrsgkcglfgqgglimfdlsstvavYSTPDLIAIIVLGIIGGIFGGLFNY 358
gi_66818603 305 TMTANLflqgftqqihdy~~~~~gvltfgvsksylYTYTELIPFMIMGIIGGLLGAIFVH 359
gi_71897153 331 TFTLNSvlsvyhgnawdlsspglinfgrfdsekmgYTIQEIPIFIFMGVVGGILGALFNA 390

                          430       440       450       460       470       480
                    ....*....|....*....|....*....|....*....|....*....|....*....|
query       386 LLDRILRVYSiiner~gapsKILLTITISIITSACSYglpwlaacspcpvgsmeecptig 444
gi_1742959  340 LTLYMTSWRRnslhkkgnrvKIIEACIISCITSAISFglpllrkcspcpesvpdsgiecp 399
gi_15215300 395 LNYWLTMFRIryih~~rpclQVIEAVLVAAVTATVAFvliyssrdcqplqggsmsyplql 452
gi_15240576 360 LVDKVLRTYSiinek~gprfKIMLVMAVSILSSCCAFglpwlsqctpcpigieegkcpsv 418
gi_19922112 419 LNTKINKFRKrfip~~wkigKVLEAVVVAMMGVTLACLmiyfindcrplgndptnnpvql 476
gi_21321026 379 LLDKVLRVYSiiner~gapfKILLTITISIITSMCSYglpwlaactpcpvdaveqcptig 437
gi_48374433 359 LLDKILRVYSiiner~gapfKILLTITISIITSMCSYglpwlaactpcpvdaveqcptvg 417
gi_66818603 360 VNVRVNHWRKklfanksklyKMIEVCVIVILSSVVCFfpalladcrpisgisgltpgtcd 419
gi_71897153 391 LNYWLTMFRIryih~~rpclQVIEAMLVAAVTAAVGFvmiycsrdcqpiqgssvayplql 448

                          490       500       510       520       530       540
                    ....*....|....*....|....*....|....*....|....*....|....*....|
query       445 rsgnfksfqc~~~~~ppghynglaslffntnddairnlfsrgtenefhMSSLFVFFIAIY 499
gi_1742959  400 rppgmygnyvnffcktdneyndlatiffntqddairnlfsaktmrefsAQSLLTFLAMFY 459
```

```
gi_15215300 453 fcadge~~~~~~~~~~~~~~ynsmaaaffntpeksvvslfhdppgsynPLTLGLFTLVYF 498
gi_15240576 419 grssiyksfqc~~~~ppnhyndlsslllntnddairnlftsrsenefhISTLAIFFVAVY 474
gi_19922112 477 fcedne~~~~~~~~~~~~~~ynavaalwfqtpeatvrslfhdppgshkILTLALFTVVYY 522
gi_21321026 438 rsgnfknfqc~~~~~ppghyndlaslffntnddairnlfsngtesefhMSTLFIFFTAVY 492
gi_48374433 418 rsgnyknfqc~~~~~ppghynglaslffntnddairnlfsngtstefqMSSLFIFFTAIY 472
gi_66818603 420 pgddstlilnqf~~ncaegyynpmatltlttlenslqivfsrstniftAQTLLTFSIFYY 477
gi_71897153 449 fcadge~~~~~~~~~~~~~~ynsmataffntpeksvvnlfhdppgsynPMTLGMFTLMYF 494

                          550       560       570       580       590       600
                 ....*....|....*....|....*....|....*....|....*....|....*....|
query       500 CLGLVTYGiAVPSGLFIPVILAGATYGRIVGTLlgpm~~~~sdiDPGLFALLGAASFLGG 555
gi_1742959  460 TLAVVTFGtAVPAGQFVPGIMIGSTYGRLVGMFvvrfy~kklniEEGTYALLGAASFLGG 518
gi_15215300 499 FLACWTYGlTVSAGVFIPSLLIGAAWGRLFGISlsyltgaaiwaDPGKYALMGAAAQLGG 558
gi_15240576 475 CLGIITYGiAIPSGLFIPVILAGASYGRLVGRLlgpv~~~~sqlDVGLFSLLGAASFLGG 530
gi_19922112 523 VLSCATFGlNVSLGVFIPTALVGAAWGRLLAMLtyyvfpqaeflHPGKYALIGAAANLGG 582
gi_21321026 493 CLGILTYGvAVPSGLFIPVILAGATYGRIVGTLlgsi~~~~sdlDPGLFALLGAASFLGG 548
gi_48374433 473 CLGLVTYGiAVPSGLFIPVILAGATYGRIVGTLlgsi~~~~sdlDPGLFALLGAASFLGG 528
gi_66818603 478 VLTIITSGlYVASGIFIPMMLIGSSWGRLIGIFlsky~~~ftsiDPSIYALIGAASMMAG 534
gi_71897153 495 FLACWTYGlTVSAGVFIPSLLIGAAWGRLFGISlsylskgsiwaDPGKYALMGAAAQLGG 554

                          610       620       630       640       650       660
                 ....*....|....*....|....*....|....*....|....*....|....*....|
query       556 TmRMTVSVCVILLELTNe~LHMLPLVMLVLLISKTIADCFNKGVYDqivvmkGLPFMeah 614
gi_1742959  519 SmRMTVSLCVIMVEITNn~LKLLPLIMLVLLISKAVGDAFNEGLYEvqarlkGIPLLesr 577
gi_15215300 559 IvRMTLSLTVIMMEATSn~VTYGFPIMLVLMTAKIVGDVFIEGLYDmhiqlqSVPFLhwe 617
gi_15240576 531 TmRMTVSLCVILLELTNn~LLMLPLVMLVLLISKTVADCFNRGVYDqivtmkGLPYMedh 589
gi_19922112 583 VlRMTISLSVILMETTGveTSFFFPLIIALISAKWVGDYFNEGIYDtqiqvnHVPMLtwe 642
gi_21321026 549 TmRMTVSVCVILLELTNd~LAMLPLVMLVLLISKTIADNFNKGVYDqivvmkGLPYMeah 607
gi_48374433 529 TmRMTVSVCVILLELTNd~LPMLPLVMLVLLISKTIADSFNKGVYDqivvmkGLPYMeah 587
gi_66818603 535 SlRMTISLVVIIVELTEt~TQYLLPVILSVMVGKWCGDIFNESIYEhlielkHIPYLnsq 593
gi_71897153 555 IvRMTLSLTVIMMEATGn~VTYGFPIMLVLMTAKIVGDYFVEGLYDmhiqlqSVPFLhwe 613

                          670       680       690       700       710       720
                 ....*....|....*....|....*....|....*....|....*....|....*....|
query       615 aepymrhlvasdvvsgplisfsgvekvgnivhalritghngfpvvdeppvseapelvglv 674
gi_1742959  578 pkyhmrqmiakeacqsqkvislprvirvadvasilgsnkhngfpvidhtrsgetlviglv 637
gi_15215300 618 apvtshsltarevmstpvtclrrrekvgvivdvlsdtasnhngfpvvehaddtqparlqg 677
gi_15240576 590 aepymrnlvakdvvsgalisfsrvekvgviwqalkmtrhngfpvideppfteaselcgia 649
gi_19922112 643 plpqykglkareilskpvicikirdsanyiyemlkkcdhngfpvvddvcgdrrsegrvcg 702
gi_21321026 608 aepymrhlvagdvvsgplitfsgvekvgnivhalrftghngfpvvdeppltеapelvglv 667
gi_48374433 588 aepymrhlvagdvvsgplitfsgvekvgnivhalrltghngfpvideppitetpelvglv 647
gi_66818603 594 ppnhlrkktvaeamstdvktlpeivkvktaltiletcphngfpvvmlpqlhsnsnlndsn 653
gi_71897153 614 apvtshsltarevmstpvtclrriervgtvvdilsdtssnhngfpvvesnpnttqvaglr 673

                          730       740       750       760       770       780
                 ....*....|....*....|....*....|....*....|....*....|....*....|
query       675 lrshvlvllsgrnfmkekvktsgsfvlrrfgafdfakpgsgkgmkiedldfteeememyv 734
gi_1742959  638 lrshllvllqskvdfqhsplpcdpsarnirhsfsefakpvsskglciedihltsddlemy 697
gi_15215300 678 lilrsqlivllkhkvfversnlglvqrrlrlkdfrdayprfppiqsihvsqderectmdl 737
gi_15240576 650 lrshllvllqgkkfskqrttfgsqilrsckardfgkaglgkglkiedldlseeememyvd 709
```

```
gi 19922112 703 iilrsqlivillkslyvenkrfwlpetsiqtfrdlyprfpsiksvrkldekinytvdlsm 762
gi 21321026 668 trshllvllngkmfmkdqlktsgsfvlqrfgafdfakpgsgkglkiqdldftdeememy~ 726
gi 48374433 648 trshllvllnsknfmkgrvktsgsfvlrrfgafdfakpgsgkglkiedldftdeemdmyv 707
gi 66818603 654 qlslslnlnnnnnnnnnnnnnnnnnnnnnnnnnnnnilcglilrsqlsvllrrkifnspe 713
gi 71897153 674 glilrsqlivllkhkvfveranlnlvqrrlklkdfrdayprfppiqsihvsqderecmid 733

                       790       800       810       820       830       840
                ....*....|....*....|....*....|....*....|....*....|....*....|
query       735 dlhpitntspytvvetmslakaavlfralglrhllvvpktpgrfpivgiltrhdlmpehi 794
gi 1742959  698 idlapflnpspyvvpedmsltkvynlfrqlglrhlfvvprpsrviglitrkdllieenge 757
gi 15215300 738 sefmnpspytvpqeaslprvfklfralglrhlvvvdnrnqvvglvtrkdlaryrlgkrgl 797
gi 15240576 710 lhpitntspytvletlslakaailfrqlglrhlcvvpktpgrppivgiltrhdfmpehvl 769
gi 19922112 763 fmnpspirvnphdsvprifqifralglrhllvinhenriagiitrrdfiyk~~~~~~~~~ 813
gi 21321026     ~~~~~~~~~~~~~~~~~~~~~~~~~~~~~~~~~~~~~~~~~~~~~~~~~~~~~~~~~~~~
gi 48374433 708 dlhpitntspytvvetmslakaailfrelglrhllvvpktpdrppivgiltrhdfmpehi 767
gi 66818603 714 dlknidfisdkgynlpidhtefsqelaskippihelsklitkedldmyidlrpymnfavv 773
gi 71897153 734 lsefmnpspytvpreaslprvfklfralglrhlvvvnnhnevvgmvtrkdlaryrlgkeg 793

                       850       860       870       880
                ....*....|....*....|....*....|....*....|..
query       795 hglfpnlrksh~~~~~~~~~~~~~~~~~~~~~~~~~~~~~~~ 805
gi 1742959  758 ssavelqqstsvrgrysdtatrmdaarpllddllg~~~~~~~ 792
gi 15215300 798 eelslaqt~~~~~~~~~~~~~~~~~~~~~~~~~~~~~~~~~~ 805
gi 15240576 770 glyphidplk~~~~~~~~~~~~~~~~~~~~~~~~~~~~~~~~ 779
gi 19922112     ~~~~~~~~~~~~~~~~~~~~~~~~~~~~~~~~~~~~~~~~~~
gi 21321026     ~~~~~~~~~~~~~~~~~~~~~~~~~~~~~~~~~~~~~~~~~~
gi 48374433 768 hslfpnlnphkyhsasmag~~~~~~~~~~~~~~~~~~~~~~~ 786
gi 66818603 774 siknysslseayqifrqvglrhmvvinvfnnvvgmltrkdlc 815
gi 71897153 794 leelslaqt~~~~~~~~~~~~~~~~~~~~~~~~~~~~~~~~~ 802
```
